# Automatic construction of molecular similarity networks for visual graph mining in chemical space of bioactive peptides: an unsupervised learning approach

**DOI:** 10.1038/s41598-020-75029-1

**Published:** 2020-10-22

**Authors:** Longendri Aguilera-Mendoza, Yovani Marrero-Ponce, César R. García-Jacas, Edgar Chavez, Jesus A. Beltran, Hugo A. Guillen-Ramirez, Carlos A. Brizuela

**Affiliations:** 1 Departamento de Ciencias de la Computación, Centro de Investigación Científica y de Educación Superior de Ensenada (CICESE), Baja California, 22860 Mexico; 2 Universidad San Francisco de Quito, Grupo de Medicina Molecular y Traslacional (MeM&T), Escuela de Medicina, Colegio de Ciencias de la Salud (COCSA), Av. Interoceánica Km 12 1/2 y Av. Florencia, 17-1200-841 Quito, Ecuador; 3 Grupo GINUMED, Corporacion Universitaria Rafael Nuñez. Facultad de Salud, Programa de Medicina, Cartagena, Colombia; 4 Unidad de Investigación de Diseño de Fármacos y Conectividad Molecular, Departamento de Química Física, Facultad de Farmacia, Universitat de València, Valencia, Spain; 5 Cátedras Conacyt - Departamento de Ciencias de la Computación, Centro de Investigación Científica y de Educación Superior de Ensenada (CICESE), Ensenada, Baja California, Mexico; 6 Department of Informatics, University of California, Irvine, Irvine, CA USA; 7 Department of BioMedical Research (DBMR), University of Bern, Bern, 3008 Switzerland; 8 Department of Medical Oncology, Inselspital, University Hospital and University of Bern, 3010 Bern, Switzerland

**Keywords:** Data mining, Protein analysis, Computational biology and bioinformatics

## Abstract

The increasing interest in bioactive peptides with therapeutic potentials has been reflected in a large variety of biological databases published over the last years. However, the knowledge discovery process from these heterogeneous data sources is a nontrivial task, becoming the essence of our research endeavor. Therefore, we devise a unified data model based on molecular similarity networks for representing a chemical reference space of bioactive peptides, having an implicit knowledge that is currently not explicitly accessed in existing biological databases. Indeed, our main contribution is a novel workflow for the automatic construction of such similarity networks, enabling visual graph mining techniques to uncover new insights from the “ocean” of known bioactive peptides. The workflow presented here relies on the following sequential steps: (i) calculation of molecular descriptors by applying statistical and aggregation operators on amino acid property vectors; (ii) a two-stage unsupervised feature selection method to identify an optimized subset of descriptors using the concepts of entropy and mutual information; (iii) generation of sparse networks where nodes represent bioactive peptides, and edges between two nodes denote their pairwise similarity/distance relationships in the defined descriptor space; and (iv) exploratory analysis using visual inspection in combination with clustering and network science techniques. For practical purposes, the proposed workflow has been implemented in our visual analytics software tool (http://mobiosd-hub.com/starpep/), to assist researchers in extracting useful information from an integrated collection of 45120 bioactive peptides, which is one of the largest and most diverse data in its field. Finally, we illustrate the applicability of the proposed workflow for discovering central nodes in molecular similarity networks that may represent a biologically relevant chemical space known to date.

## Introduction

During the last years, a growing interest has emerged for the development of Bioactive Peptides (BPs) as potential drugs impacting on human health^1,2^. As a consequence, a lot of BPs and their biological annotations have been collected from the literature into a large variety of biological databases, providing a valuable ground for knowledge discovery. Naturally, the domain experts in the field of biomedical research need to mine knowledge hidden in those heterogeneous data sources. However, to exploit the available data, most of the previous reports^3–6^ are primarily focused on the field of supervised machine learning for building predictive models from a set of training instances. These supervised based models are very suitable to predict the biological activities for new molecules whose functions to be predicted are unknown. By contrast, in unsupervised methods, the input data are not labeled with the corresponding outcome, and there is no response to supervise the learning process. So, in this other field of machine learning, the goal is finding interpretable associations and patterns among available data elements.

In this report, we focus on employing unsupervised learning methods for turning existing data of BPs into information not appreciated before. As a foundation for this task, we already have introduced two valuable resources: starPepDB and starPep toolbox^7^. StarPepDB is an integrated graph database of existing individual databases for a better understanding of the universe of BPs. This comprehensive graph database is one of the largest and most diverse data in its field. It is comprised mainly of 45,120 nodes representing peptide compounds, while additional nodes are connected to these primary nodes for describing metadata, i.e., biological annotations. Moreover, the software named starPep toolbox helps researchers to understand the integrated data through visual network analysis.

Now we continue to extend the implemented visual analysis process to let the end-users gain insight into a graph-based representation of chemical space for studying BPs. Whereas the term chemical space refers to the ensemble of all possible molecules, either natural or synthetically accessible product^8,9^, a realistic representation focuses on confined regions of such theoretical space. Still, chemical space is so vast that its complete enumeration and visual inspection goes beyond human comprehension. Nonetheless, not all likely compounds are biologically active, and the biologically relevant chemical space is where the bioactive compounds reside^8^. Thus, the analysis and visualization of chemical space covered by known bioactive peptides (those reported thus far) may play an essential role in decision-making when searching for new peptide-based drugs^10,11^.

To visualize a chemical space, one of the most common approaches has been a coordinate-based map^12^, where a set of numerical features (descriptors) characterizes each compound. That is, mathematically, for each chemical compound corresponds a point in a multi-dimensional descriptor space that requires a dimensionality reduction technique for a proper visualization in 2*D* or 3*D* maps. For instance, Principal Component Analysis^13^ (PCA) is a popular technique that seeks a small number of principal components expressed as a linear combination of original features, explaining as much of data variability as possible. In this way, the 2D (3D) map may project the information contained in two (three) of the first principal components of descriptor space, ending in a loss of information.

As an alternative to the coordinate-based map, the so-called Chemical Space Networks (CSNs)^14–16^ have been proposed as coordinate-free representations for analyzing and visualizing the chemical space without reducing dimensionality. In such CSNs, nodes represent molecular entities while an edge may indicate a similarity relationship between two compounds. A key point of these networks relies on the potential application of graph-based algorithms, which can be used in combination with interactive visualization to enable the domain-expert in the field of Biomedical research to find previously unknown and useful information^17,18^.

Note that the interactive visualization aims to incorporate the human factor in a process that has been called the Visual Information Seeking Mantra^19^: overview first, zoom and filter, then details-on-demand. For instance, under this mantra, the interactive visual interface firstly provides an overview of the chemical space. Secondly, the user focuses on molecules of interest and filter out uninteresting peptide compounds. Then, to gain a better understanding of the data, the user accesses only the details of the interesting molecules.

As user interaction relies on a visual representation of chemical space, mapping the raw data of BPs into a multi-dimensional descriptor space should be robust and not accidental to derive consistent conclusions. A grand challenge is to find the most appropriate chemical space representation since there are many different ways of coding molecules to denote the descriptor space, and it is unknown which set of MDs is the best one. So, the success of the visual exploration approach depends on two main aspects: (i) a particular choice of molecular descriptors (MDs), and (ii) proper visualization techniques to display information related to the chosen MDs. Nonetheless, even in cases where the chemical space visualization is unclear, we argue that the interactive exploration, in combination with suitable algorithms may help humans to understand and reason about the chemical data under consideration.

With the study presented here, we tackle the automatic construction of molecular similarity networks for navigating and mining a chemical reference space from our comprehensive collection of BPs. In such molecular similarity networks, each node representing BPs is characterized by a set of molecular descriptors, and edges between nodes denote their pairwise similarity/distance relationships. Once this meaningful network has been created, it can provide insight into drug design for decision-makers^20,21^.

## Results

**Figure 1:**
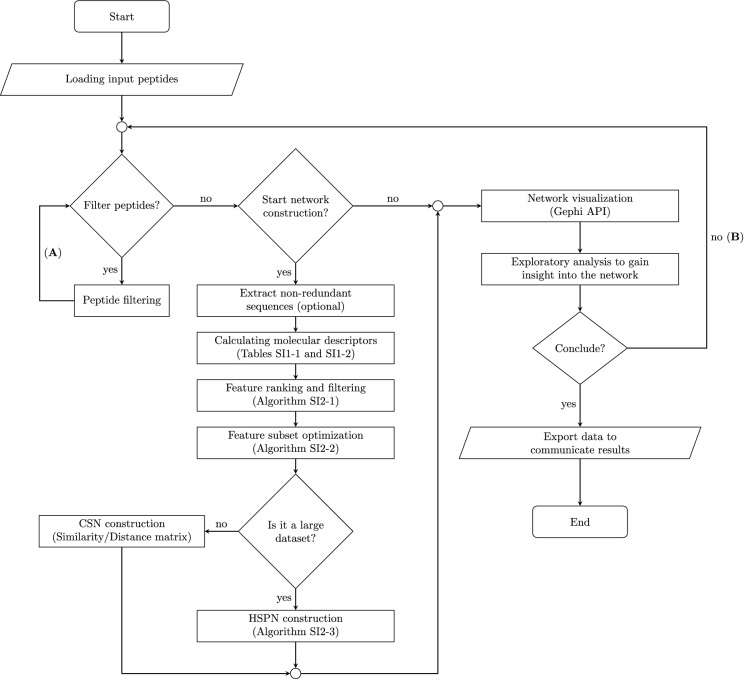
A flow diagram guiding the automatic construction and visual graph mining of similarity networks. The similarity network can be automatically generated for the first time and just reused, or even it could be regenerated after that. Whereas the mining task is made up of two nested loops, in order, from the most internal to the most external: (A) data manipulation loop, and (B) visual feedback loop. This figure has been created using the package TikZ (version 0.9f 2018-11-19) in Latex, available at https://www.ctan.org/pkg/tikz-cd.

The corresponding steps of a proposed workflow (Fig. 1) have been implemented in our visual analytics software starPep toolbox^7^ (http://mobiosd-hub.com/starpep/), intended to be used by researchers. As described next, we experimented with the proposed workflow for assessing and tuning the automatic construction of similarity networks from raw amino acid sequences of BPs. We also illustrate the usage of the proposed workflow for turning known BPs into information that is hidden in the existing biological databases. For instance, we generated molecular similarity networks for finding out central nodes that may represent a biologically relevant chemical space of anticancer peptides.

### Comparative study of molecular descriptors

Various molecular descriptors can be derived from peptide sequences by applying statistical and aggregation operators on amino acid property vectors (SI1-2). Indeed, hundreds or even thousands of these descriptors can be calculated using the starPep toolbox^7^ during workflow execution (Fig. 1). Since many of the descriptors to be calculated are different from those frequently employed in the literature, we performed a comparison with several other peptide descriptor families available in the recent software package iFeature^22^. For this comparative study, 748 peptide sequences (SI1-3) with 30 amino acids were recovered from starPepDB^7^.

On the one hand, the iFeature descriptors on the peptide dataset are comprised of 3340 amino acid composition (AAC) indices, 310 grouped amino acid composition (GAAC) indices, 360 autocorrelation indices, 273 composition-transition-distribution (C/T/D) indices, 100 quasi-sequence-order (Q-S-O) indices, 85 pseudo-amino acid composition (PAAC) indices (85), 430 PseKRAAC indices, 15930 amino acid (AA) indices, 600 BLOSUM62 indices, and 150 Z-scale indices. These external descriptors are given in SI1-4. On the other hand, the starPep descriptors were calculated by selecting all the available amino acid properties (e.g., heat of formation, side chain mass, etc.), all groups of amino acid types (e.g., aliphatic, aromatic, unfolding, etc.), and traditional (excepting those based on GOWAWA and Choquet integral) plus neighborhood (*k* neighbors up to 6) aggregation operators.

After generating peptide descriptors, a Shannon Entropy (SE)-based variability analysis^23^ (VA) was carried out in order to quantify and compare the information content codified by the calculated descriptors. In this way, relevant descriptors can be identified according to the principle that high-entropy values correspond to those descriptors with a good ability to discriminate among structurally different peptides, while low-entropy values are indicative of the opposite^24^. Notice that the discretization scheme adopted for entropy calculation is equal to 748 bins, which is the number of peptides accounted for. Thus, the maximum entropy for each descriptor is equal to 9.55 bits.

The IMMAN software^25^ was used to perform the VA of descriptors calculated by iFeature and starPep toolbox. It should be pointed out that all iFeature descriptor families were joined into a single data set for a proper comparison. Also, a correlation filter was applied for the computed descriptors. Hence, some redundant features were removed using the Spearman correlation-based filter with a threshold equal to 0.95. Consequently, a total of 12018 iFeature descriptors and 8416 starPep descriptors were retained (SI1-5), being these two sets of descriptors the ones used in the VA.

**Figure 2:**
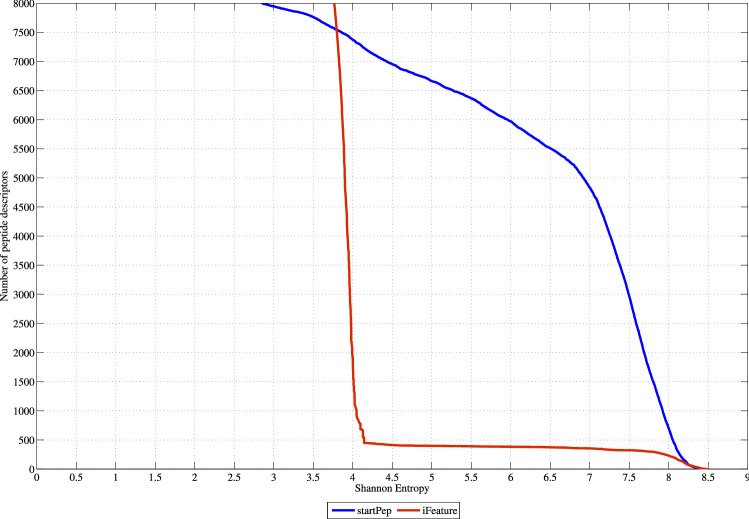
Shannon’s entropy distribution for the iFeature descriptors versus starPep descriptors. This figure has been created using the R software package (version 3.5.1), available at https://cran.r-project.org/.

As a result of the VA, Fig. 2 depicts the SE distribution corresponding to the best 8000 ranked descriptors according to their SE values. As it can be noted, the starPep descriptors present better ability to discriminate among structurally different peptide sequences than the iFeature descriptors, since the formers have better SE distribution than the latter. The analyzed starPep descriptors present an average SE equal to 7.59 bits, whereas the iFeature descriptors have an average SE equal to 4.29 bits. More specifically, if we zoom in to the best 500 ranked descriptors, those from starPep always present SE values greater than 8 bits (83.77% of the maximum entropy), whereas only 231 iFeature descriptors are above the threshold aforementioned. In general, it can be concluded that the starPep descriptors have better information content than several types of peptide descriptor families. Thus, the starPep descriptors are useful for the mathematical characterization of a chemical space of BPs.

**Figure 3:**
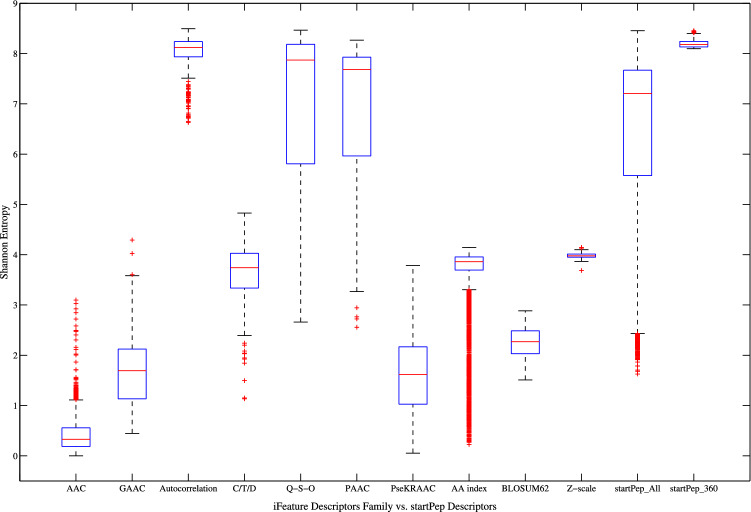
Boxplots showing distributions of Shannon’s entropy for the iFeature descriptor families, as well as the starPep descriptors. This figure has been created using the R software package (version 3.5.1), available at https://cran.r-project.org/.

Moreover, Fig. 3 shows the boxplot graphics corresponding to each iFeature descriptor family (without removing correlated descriptors), as well as the corresponding ones to the starPep descriptors. It can be noted in this figure that the best iFeature descriptor families correspond to autocorrelation, Q-S-O, and PAAC indices, being the former the best of all. If these descriptor families are compared to the starPep descriptors (denoted as starPep_All), it can be observed that the 100 Q-S-O indices and 85 PAAC indices present a similar distribution regarding the 8416 starPep descriptors analyzed, whereas the 360 autocorrelation indices from iFeature present a better distribution. However, if the best 360 ranked descriptors from starPep are analyzed (denoted as starPep_360), then it can be observed that they present better distribution than the 360 autocorrelation indices from iFeature.

As a complement to the previous results, a linear independence analysis was performed by means of the PCA^13^ method. To this end, the iFeature and starPep descriptors with SE values greater than 8 bits were selected from the descriptor sets considered in the VA. Thereby, the input data for PCA is comprised of 700 starPep descriptors and 231 iFeature descriptors (SI1-6). By applying the PCA method on this feature space, orthogonal descriptors are strongly loaded (*p*-value ≥ 0.7) in different components, while collinear descriptors are loaded in the same component. Supplementary Information SI1-7 contains the eigenvalues and the percentages of the explained variance by the 11 principal components (PCs) obtained, which explain approximately 54.46% of the cumulative variance.

From the result table of PCA (SI1-7), we observed that some of the iFeature and starPep descriptors analyzed show collinearity in PC1 (16.64%) and PC4 (4.47%). For instance, in PC1, Q-S-O indices based on the Grantham chemical distance matrix^26^, for lag values from 1 to 13, are collinear with starPep descriptors encoding information related to amino acids favoring alpha-helix and uncharged polar amino acids, which were weighted with the third z-scale property. Moreover, in PC4,  a single Q-S-O index based on the Schneider–Wrede physicochemical distance matrix^27^, for lag equals to 12, codified collinear information to starPep descriptors related to the isoelectric point and hydrophilicity amino acid properties. Additionally, the results obtained also indicate that the starPep descriptors have exclusive loads in the remaining components determined. That is, the iFeature descriptors studied did not present unique loadings in either of the components obtained.

Finally, three important conclusions can be drawn from the study so far: (1) the starPep descriptors seem to codify a degree of structural information captured by the iFeature descriptors, evidenced by the collinearity between the two groups; (2) there is structural information encoded by the starPep descriptors that is linearly independent to the iFeature descriptors; and (3) the iFeature descriptors seem not to codify different information respect to the starPep descriptors. These early outcomes demonstrate the applicability of the theoretical aspects of the starPep descriptors, and therefore, they should be useful in the construction of similarity networks.

### Feature ranking and filtering

In this part of our experiments, we used starPep toolbox^7^ to initially compute 830 molecular descriptors on various datasets with different numbers of instances (*n*). For each dataset, the original descriptors and its corresponding reduced feature subsets were used as experimental data^28^ to assess the early stage of the feature selection process (Table 1). In this preliminary study, the calculated descriptors were ranked according to their entropy values, using the number of bins equal to *n*, and the entropy cutoff value ($\theta _1$) was set to 10% of maximum entropy (i.e., $\theta _1 = 0.1 * \log n$) for removing irrelevant features from input datasets (Algorithm SI2-1). In general, there were at most 25 useless descriptors having information content less than the cutoff value, and they were removed.

After removing irrelevant features, all input datasets approximately kept 800 molecular descriptors sorted by their entropy values. Following that order, redundant features are removed, as described in Algorithm SI2-1. At this step, for the two correlation methods considered (Pearson’s or Spearman’s coefficient), several cutoff values were used to assess their effect at removing redundant variables (Table 1). Besides, Procrustes analysis^29^ was employed to check how much the complete set of descriptors can be reduced while preserving the data structure between the original and reduced descriptor space. On comparing the two descriptor spaces, the Procrustes goodness-of-fit is calculated between the first PCs of both the original and reduced sets of variables. The first 50 PCs were used since they explained at least 80% of the variance in the original data.

**Table 1:** Exploring the effect of changing the correlation method (Pearson’s or Spearman’s coefficient) and cutoff value ($\theta _2$) for assessing the similarity between subsets of candidate features and the original ones. Datasets and descriptors used in this experimental study are publicly available^28^.

Datasets^a,b^	Instances	Correlation threshold ($\theta _2$)				
		Number of filtered descriptors (goodness-of-fit)				
		0.5	0.6	0.7	0.8	0.9
		Pearson				
Overall_NR98	32300	108 (0.36)	156 (0.27)	199 (0.22)	275 (0.15)	374 (0.09)
Overall_NR90	22512	111 (0.35)	160 (0.27)	208 (0.21)	279 (0.15)	372 (0.1)
Overall_NR70	14559	108 (0.37)	150 (0.29)	207 (0.21)	276 (0.14)	382 (0.08)
Overall_NR50	9428	108 (0.37)	155 (0.28)	198 (0.21)	279 (0.14)	378 (0.09)
Overall_NR30	4735	112 (0.37)	155 (0.28)	205 (0.19)	281 (0.14)	385 (0.08)
Antibacterial_NR98	10303	94 (0.35)	147 (0.25)	191 (0.19)	282 (0.13)	379 (0.09)
Antifungal_NR98	4546	101 (0.36)	150 (0.26)	202 (0.2)	272 (0.14)	380 (0.08)
Antiviral_NR98	3849	100 (0.37)	143 (0.28)	186 (0.21)	280 (0.13)	393 (0.08)
Anticancer_NR98	1557	98 (0.35)	132 (0.28)	188 (0.19)	274 (0.12)	395 (0.07)
Antiparasitic_NR98	501	111 (0.28)	153 (0.21)	211 (0.15)	298 (0.1)	402 (0.06)
Averages		105 (0.35)	150 (0.27)	200 (0.2)	280 (0.13)	384 (0.08)
		**Spearman**				
Overall_NR98	32300	94 (0.38)	126 (0.3)	177 (0.23)	235 (0.18)	338(0.09)
Overall_NR90	22512	95 (0.37)	127 (0.31)	174 (0.24)	241 (0.18)	327 (0.1)
Overall_NR70	14559	94 (0.38)	127 (0.32)	175 (0.25)	235 (0.18)	324 (0.1)
Overall_NR50	9428	95 (0.39)	132 (0.31)	174 (0.26)	241 (0.18)	327 (0.1)
Overall_NR30	4735	99 (0.37)	134 (0.29)	188 (0.22)	241 (0.18)	342 (0.1)
Antibacterial_NR98	10303	90 (0.35)	121 (0.29)	170 (0.22)	232 (0.16)	334 (0.1)
Antifungal_NR98	4546	87 (0.39)	122 (0.29)	177 (0.22)	236 (0.16)	338 (0.09)
Antiviral_NR98	3849	99 (0.35)	133 (0.3)	172 (0.22)	241 (0.16)	346 (0.09)
Anticancer_NR98	1557	92 (0.37)	128 (0.27)	172 (0.22)	245 (0.14)	352 (0.08)
Antiparasitic_NR98	501	94 (0.32)	129 (0.24)	179 (0.17)	251 (0.12)	361 (0.07)
Averages		94 (0.37)	128 (0.29)	176 (0.23)	240 (0.16)	339 (0.09)

^a^These datasets were retrieved from the database StarPepDB^7^.

^b^NR stands for Non-Redundant at a given percentage (number) of sequence identity. To carry out the sequence identity comparisons, we used a local alignment algorithm (Smith–Waterman^30^) implemented in BioJava^31^, with the BLOSUM62 substitution matrix.

**Figure 4:**
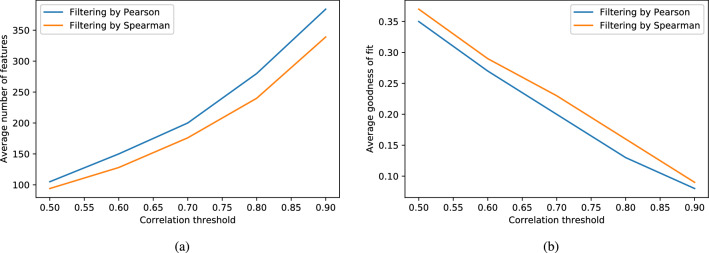
Exploring the effect of changing the similarity threshold: (**a**) the average number of retained features is shown as an increasing function in similarity threshold; and, (**b**) the average goodness-of-fit between the 50 PCs of both the original and reduced features is shown as a decreasing function in similarity threshold. This figure has been created using the Python library Matplotlib (version 3.3.0), available at https://matplotlib.org/users/installing.html.

As can be seen in Table 1 and Fig. 4, the initial amount of calculated descriptors can be drastically reduced, providing some level of redundancy elimination in the resulting set of variables. As it is expected, decreasing the correlation threshold led to reducing the number of filtered features, being the Spearman coefficient, the one that least features retained. Moreover, looking at the Procrustes analysis, a low correlation threshold affects the goodness-of-fit between the original and reduced data. Whereas setting a high correlation threshold results in a better match between the reduced data structure and the original one, which is desired at this stage.

By analyzing Table 1, it can be noted that using Spearman and fixing the correlation threshold to 0.9 still yields a high number (> 300) of filtered descriptors; but a lower-number of descriptors (< 300) is retained when the correlation threshold is equal to 0.80, while the average goodness-of-fit is less than 0.2. It means that the resulting lower-dimensional space represents well enough the data structure of the original descriptor space. Thus, from now on, the Spearman correlation-based filter with a threshold equal to 0.8 will be the one used during the first stage of the feature selection process to compute a candidate set.

### Feature subset optimization

So far, the resulting candidate set is still not enough lower-dimensional for the similarity network construction. Thus, the goal of the second stage is not the data structure preservation of the original descriptor space, but the optimization of a merit function (Eq. 7) to obtain an adequate feature subset. Table 2 shows the results for Algorithm SI2-2, where the best subset found for each dataset is characterized by the number of features and merit score. In general, the best-retained features are between 30 and 50, taking the square root of the number of instances as the number of bins (square-root choice^32^) for computing entropy and mutual information at this stage. Although there is no “correct” answer for the number of bins, the square-root choice led to good results in terms of the size of optimized subsets and merit scores, if compared with other alternatives (data not shown here).

**Table 2:** Performance of feature subset optimization.

Datasets	Instances (*n*)	Number of bins^a^ ($\lfloor \sqrt{n} \rfloor$)	Best subset found		CPU Time^b^ (hh:mm:ss)
			Number of features	Merit scores	
Overall_NR98	32300	179	39	3.985	12:07:03
Overall_NR90	22512	150	40	3.87	05:17:40
Overall_NR70	14559	120	42	3.667	01:27:46
Overall_NR50	9428	97	39	3.474	00:34:04
Overall_NR30	4735	68	33	3.13	00:08:12
Antibacterial_NR98	10303	101	45	3.41	00:38:57
Antifungal_NR98	4546	67	39	3.059	00:07:17
Antiviral_NR98	3849	62	45	3.031	00:05:38
Anticancer_NR98	1557	39	36	2.588	00:00:50
Antiparasitic_NR98	501	22	40	2.11	00:00:05

^a^The number of bins (data intervals) when constructing the histograms for computing the entropy and mutual information.

^b^The feature subset optimization was performed in a Mac Pro Server with 2 x Intel Xeon Processor 2.66 GHz 6-cores, and memory 64 GB.

**Table 3:** Comparison among the best subsets found and top-*k* ranked descriptors.

Datasets	Instances	Best merit scores^a^	Merit scores of top-*k* ranked descriptors^b^				
			*k*				
			20	30	40	50	60
Overall_NR98	32300	3.985	3.92	3.96	3.962	3.958	3.942
Overall_NR90	22512	3.87	3.79	3.827	3.829	3.822	3.807
Overall_NR70	14559	3.667	3.577	3.608	3.616	3.607	3.589
Overall_NR50	9428	3.474	3.368	3.396	3.406	3.403	3.388
Overall_NR30	4735	3.13	3.051	3.057	3.044	3.038	3.035
Antibacterial_NR98	10303	3.41	3.336	3.361	3.364	3.356	3.334
Antifungal_NR98	4546	3.059	2.98	3.018	3.01	3.011	2.993
Antiviral_NR98	3849	3.031	2.956	2.992	3.001	3.001	2.995
Anticancer_NR98	1557	2.588	2.517	2.552	2.55	2.54	2.529
Antiparasitic_NR98	501	2.11	2.042	2.063	2.06	2.065	2.061
Averages		3.232	3.154	3.183	3.184	3.180	3.167

^a^Merit scores of best subsets found.

^*b*^Merit scores of the top-k ranked descriptors from the candidate feature set.

**Figure 5:**
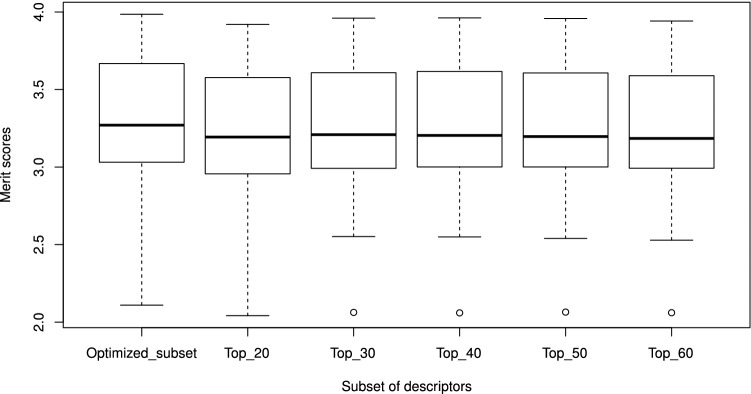
Box plot of merit scores. This figure has been created using the R software package (version 3.5.1), available at https://cran.r-project.org/.

Additionally, Table 3 shows a comparison between the optimized subsets and the top-*k* ranked descriptors (with *k* between 20 and 60) from the full set of candidate features (without being optimized), according to their entropy values in descending order. For this comparison, a Pairwise Wilcoxon Rank Sum Test^33^ with Hochberg^34^ correction was used to evaluate differences among the selected groups of descriptors in Table 3. The statistical test revealed that the optimized subset is significantly better ($p < 0.05$) than the top-*k* descriptors. Also, there are no significant differences between the top 30, top 40, and top 50 descriptors, which are likely to give the second-best scores. Furthermore, Fig. 5 summarizes the distribution of merit scores, and there can be observed atypical merit scores (outliers) for most groups of top-*k* descriptors. The presence of these outliers may suggest that the selection of those top-*k* descriptors may be affected by a small number of instances, such as the Antiparasitic_NR98 dataset (Table 3). Therefore, it can be concluded that the optimized descriptors (SI3-1) are suitable to characterize the chemical space of BPs.

**Figure 6:**
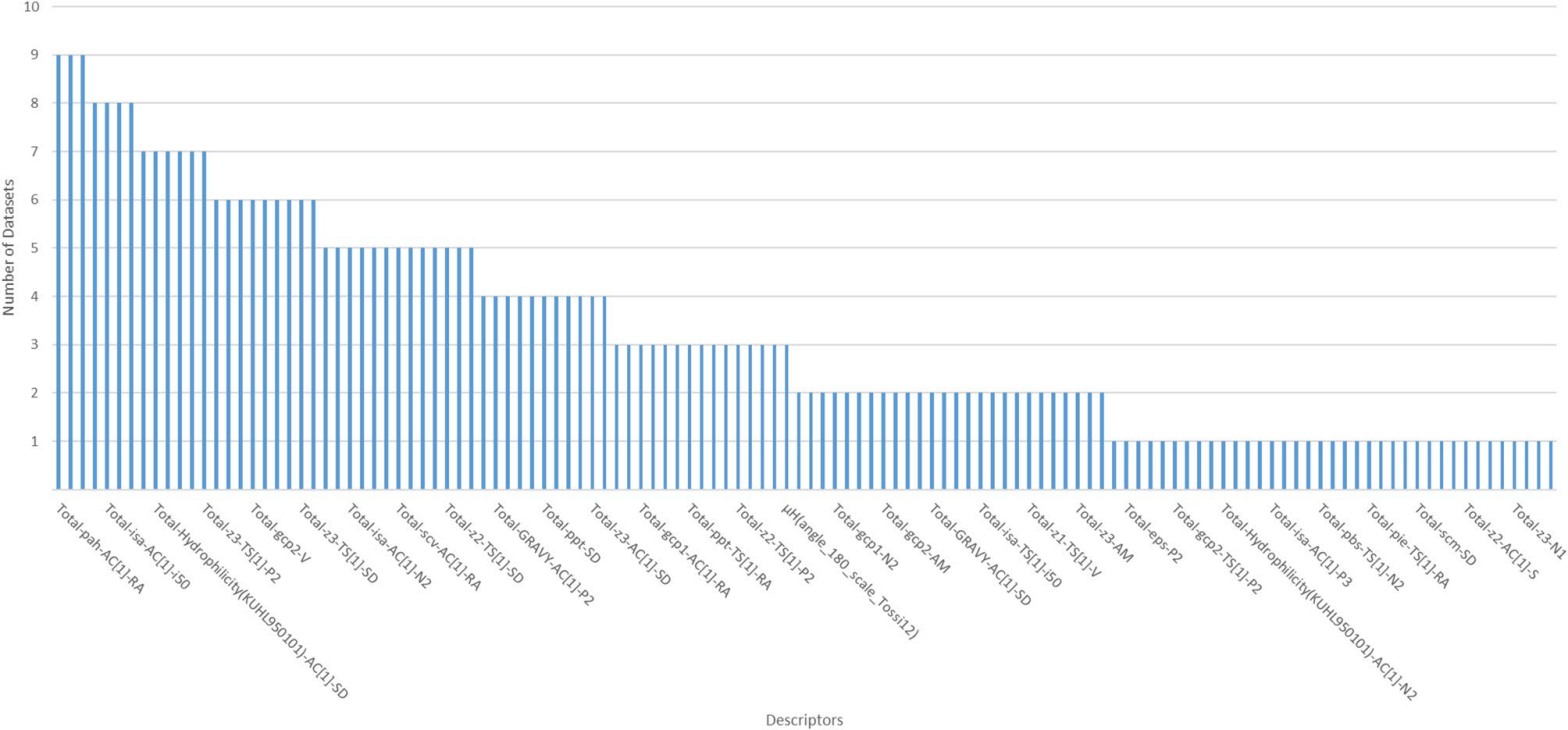
Number of distinct datasets that include a selected descriptor. This figure has been created in Excel 2016, available at https://www.microsoft.com/es-mx/software-download/office.

To illustrate that the optimized descriptors adequately characterize a particular chemical space, we have accounted for the number of distinct datasets that include a selected descriptor. The results are presented in Fig. 6. The x-axis lists descriptors sorted by the most common ones in all datasets. The y-axis represents the number of datasets that include the specified descriptors. It can be noted that there are just three descriptors repeated in nine out of the ten datasets. Besides, the trend in the chart indicates that the majority of descriptors reappear only in a few databases. Indeed, many of them appear solely in a single dataset.

### Generating molecular similarity networks

According to the above results, we used the optimized descriptors (SI3-1) to built molecular similarity networks based on (i) the traditional CSNs^14–16^, or (ii) the sparse HSPNs^35^. Note that whereas the fully connected CSN at threshold $t=0$ is prohibitive for large datasets, an alternative connected network may be successfully generated based on HSPNs (Algorithm SI2-3). The feasibility of building such HSPNs from the experimental datasets is illustrated in Table 4. It can be observed that the generated HSPNs achieve low-density levels in all cases, including large datasets.

**Table 4:** Summary for the alternative HSPNs generated at threshold $t=0$.

Datasets	Nodes	Max	Similarity relationships^b^	
		distance^a^	Edges	Network density
Overall_NR98	32300	29.80	353 213	6.8E-4
Overall_NR90	22512	27.22	242 227	9.6E-4
Overall_NR70	14559	27.03	157 590	0.001
Overall_NR50	9428	27.01	102 226	0.002
Overall_NR30	4735	23.65	46338	0.004
Antibacterial_NR98	10303	27.95	95605	0.002
Antifungal_NR98	4546	24.00	36765	0.004
Antiviral_NR98	3849	24.12	31709	0.004
Anticancer_NR98	1557	21.56	9552	0.008
Antiparasitic_NR98	501	19.13	2560	0.02

^a^The maximum distance between two points in the defined descriptors space.

^b^Similarity relationships at $t=0$. The HSPN at $t = 0$ is a connected subnetwork having a low-density level in all datasets.

**Figure 7:**
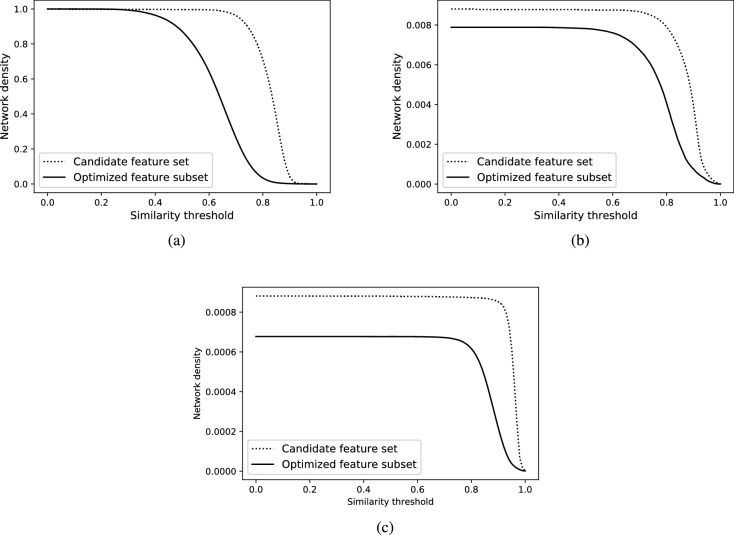
Examining the impact of using the optimized feature subset versus the full set of candidate features for similarity network construction. Density curves were generated at varying the similarity threshold in the following networks: (**a**) CSNs from Anticancer_NR98, (**b**) HSPNs from Anticancer_NR98, and (**c**) HSPNs from Overall_NR98. In contrast to CSNs, the natural density of HSPNs is low at any threshold, even for $t = 0$. This figure has been created using Python Matplotlib (version 3.3.0), available at https://matplotlib.org/users/installing.html.

**Figure 8:**
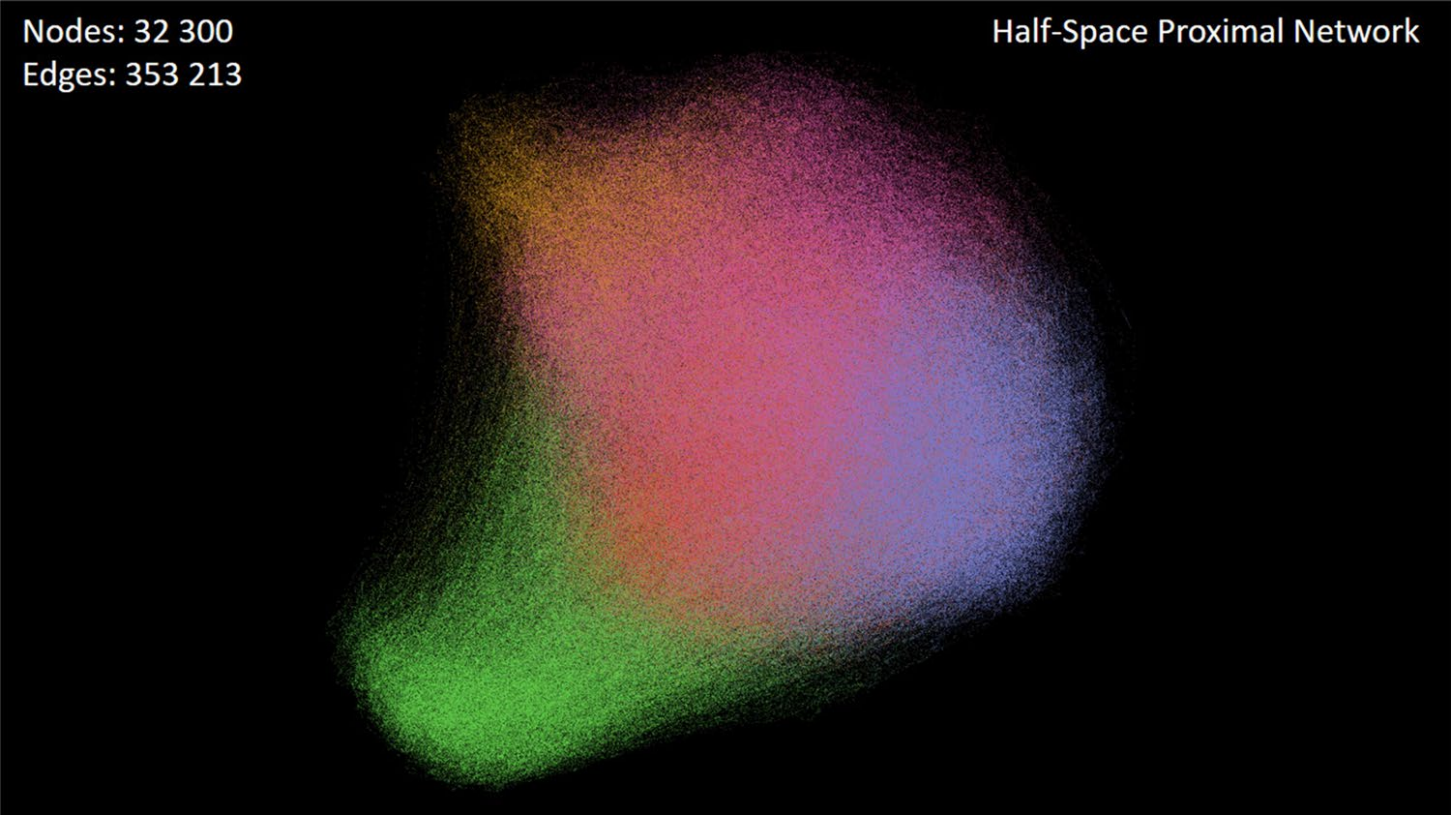
Visualizing the similarity network (HSPN) of a vast set of BPs (Overall_NR98) at threshold $t=0$, using the ForceAtlas2 layout algorithm^36^. This figure has been created using the software starPep toolbox (version 0.8), available at http://mobiosd-hub.com/starpep/, and PowerPoint 2016 available at https://www.microsoft.com/es-mx/software-download/office.

Furthermore, the impact of optimized descriptors can also be seen in the construction of the molecular similarity networks, either CSNs or HSPNs. As depicted in Fig. 7, by interpreting the performance of descriptors as a function of network density, the optimized feature subset yields lower density levels with a smoother appearance than the density curves of the complete candidate feature set. This shows that, even for large datasets such as Overall_NR98 (Fig. 7c), the optimized descriptors are suitable for generating a lower-density similarity network to be analyzed by visual inspection (Fig. 8).

### Visual analysis of chemical space

Visualization and interactive exploration of the generated similarity networks may serve to form a visual image into the mind aimed at facilitating analytical reasoning not possible before. However, understanding networks through visual inspection is not a straightforward process^37^, and thus we propose a systematic network exploration using a combination of clustering and network science techniques. During this exploration process, three aspects of visual inspection are useful for aiding human thinking: positioning, filtering, and customizing the appearance of nodes^7^.

To facilitate the above-mentioned network analysis, the proposed workflow in Fig. 1 has been implemented in the software starPep toolbox (http://mobiosd-hub.com/starpep/), allowing end-user to generate and navigate similarity networks by their own needs and interests. On this basis, as described next, we illustrate the use of the proposed workflow to carry out three case studies for finding and analyzing relevant nodes in molecular similarity networks of BPs.

#### Case study I: navigating a biologically relevant chemical space

**Figure 9:**
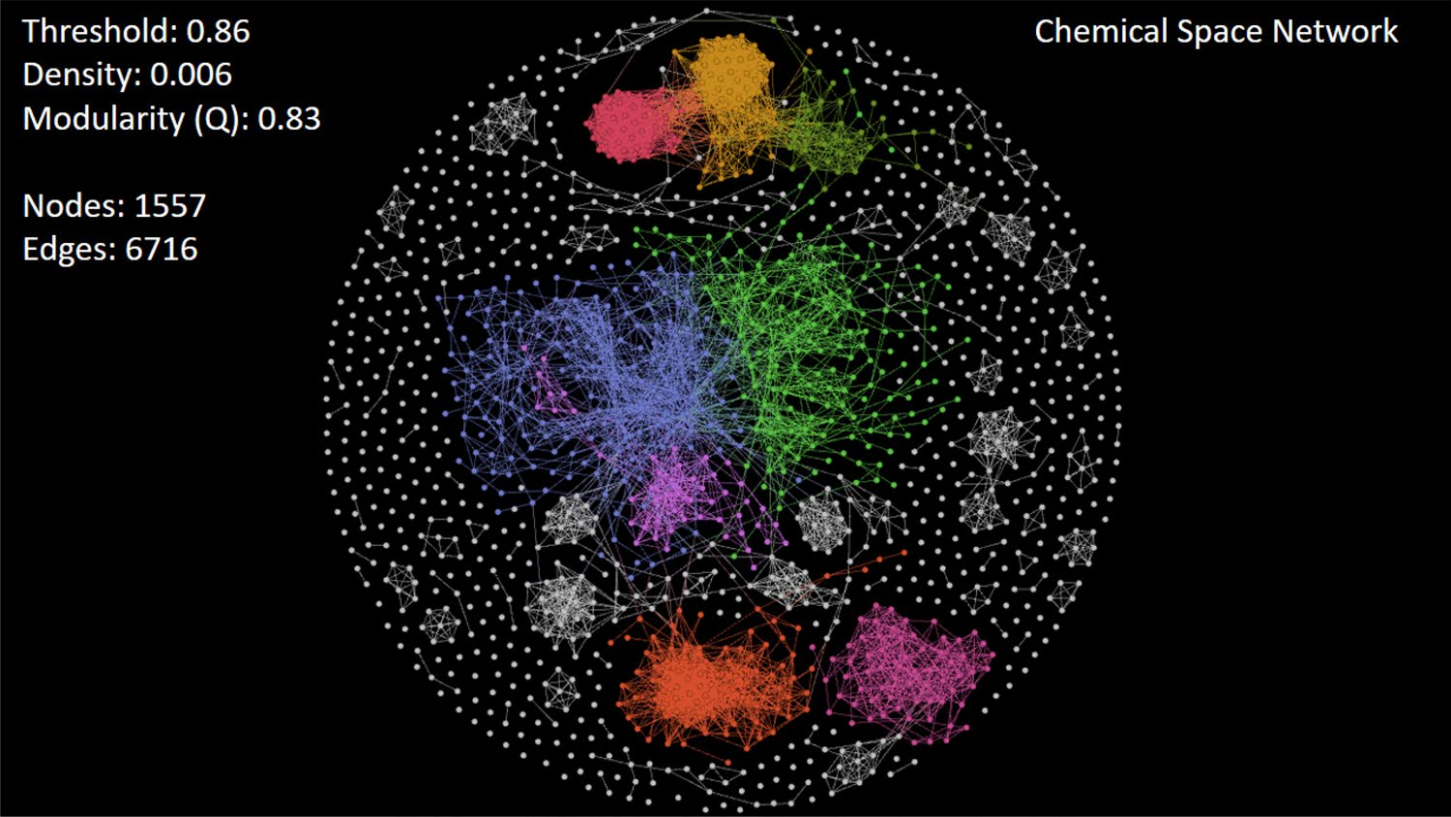
Visualizing the CSN of anticancer peptides (Anticancer_NR98) at similarity threshold of 0.86, using the Fruchterman-Reingold layout algorithm^39^. Nodes are colored according to the leading communities to which they belong. This figure has been created using the software starPep toolbox (version 0.8), available at http://mobiosd-hub.com/starpep/ , and PowerPoint 2016 available at https://www.microsoft.com/es-mx/software-download/office.

The 1557 anticancer peptides (Anticancer_NR98) to be analyzed here are given in SI4-1 (FASTA file). Also, for each peptide discovered to be a relevant node, additional information (metadata^7^) is available in SI4-2 (Excel file). As the dataset Anticancer_NR98 is not large enough, we generate traditional similarity networks (CSNs) from the similarity/distance matrix calculation. To visualize these similarity networks in a meaningful way, we examine a family of force-directed layout algorithms that can be used to spatialize the network and rearrange nodes^38^. These algorithms change the position of nodes by considering that they repulse each other, whereas similarity relationships may attract their attached nodes like springs. Particularly, the Fruchterman-Reingold algorithm^39^ was the most suitable for drawing the network of anticancer dataset (Fig. 9).

*Exploring and mining community structures*. A CSN having an arbitrary low density ($< 1\%$) can be visualized at first sight by setting a large threshold (Fig. 7a). Also, networks become more interpretable through visual inspection if having a community structure^40^. Note that communities of BPs may represent some biologically relevant regions of chemical space where bioactive compounds reside^9^. Hence, we have explored the CSNs by varying the similarity threshold until a well-defined community structure emerged. In this way, a final CSN has been analyzed by adjusting the similarity threshold to 0.86, at network density of 0.006, achieving network modularity (*Q*) of 0.83 (Fig. 9).

Based on our visual exploration, we corroborate what other authors have previously remarked^41^; there is higher network modularity at large similarity threshold. Nonetheless, the interval within which the threshold value should be explored is not fixed, and it depends on the input dataset. We also suggest that the final decision for picking a high threshold value should be made carefully to avoid an edgeless network structure (Fig. 7).

**Figure 10:**
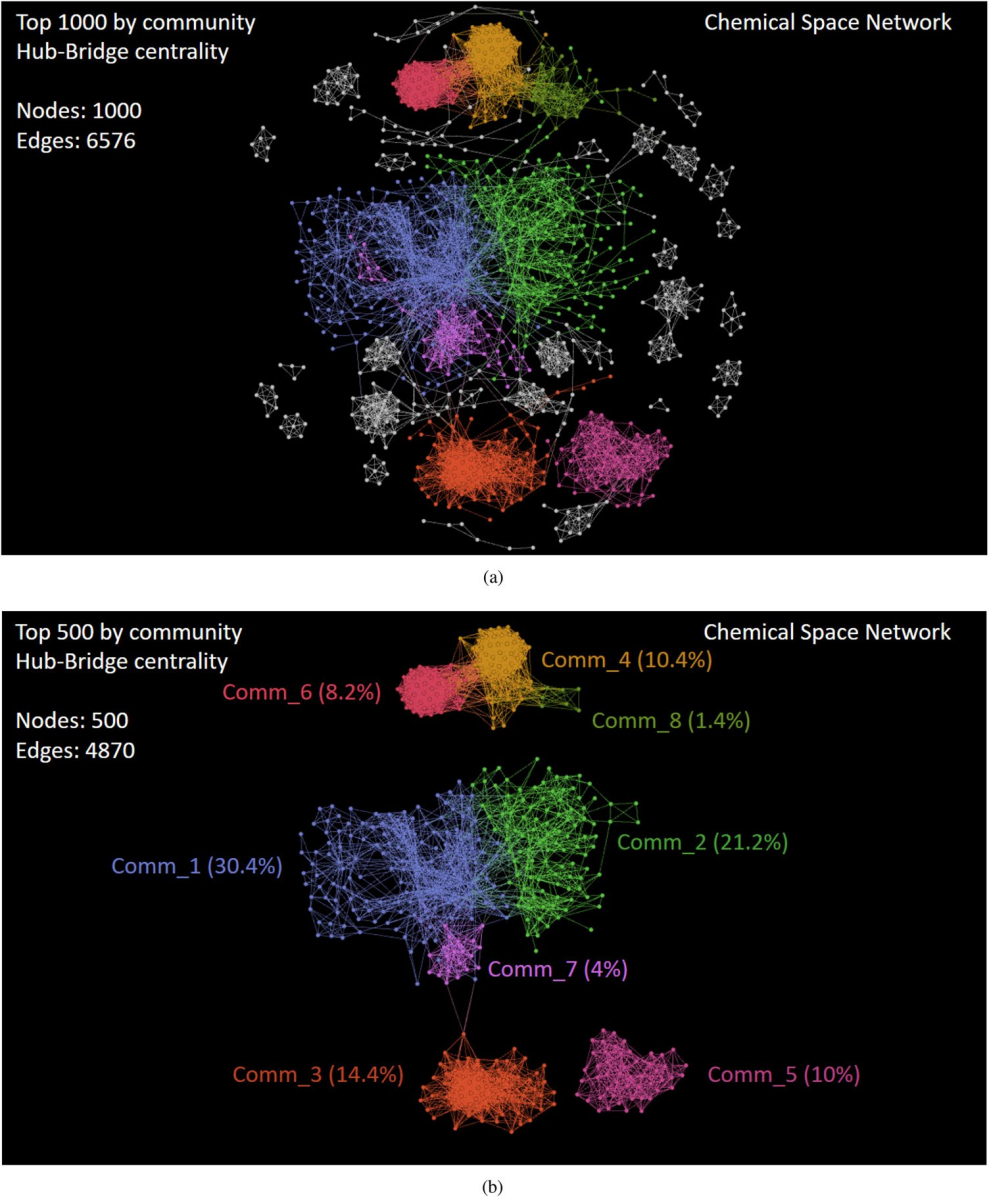
Visualizing the subnetwork of anticancer peptides having the (**a**) Top 1000 and (**b**) Top 500 ranked nodes by the Community Hub-Bridge centrality. This figure has been created using the software starPep toolbox (version 0.8), available at http://mobiosd-hub.com/starpep/, and PowerPoint 2016 available at https://www.microsoft.com/es-mx/software-download/office.

*Local centrality analysis*. Once a community structure is found, we rank nodes in decreasing order according to the community Hub-Bridge centrality measure (Eq. 13) for retaining the top-*k* of the ranked list. Particularly, the top 1000 exposes densely connected groups of nodes like cliques, which are defined to be complete subgraphs (Fig. 10a). These related sequences may be forming families in the chemical space of anticancer peptides, and detecting them may be of use in future works^42,43^.

**Figure 11:**
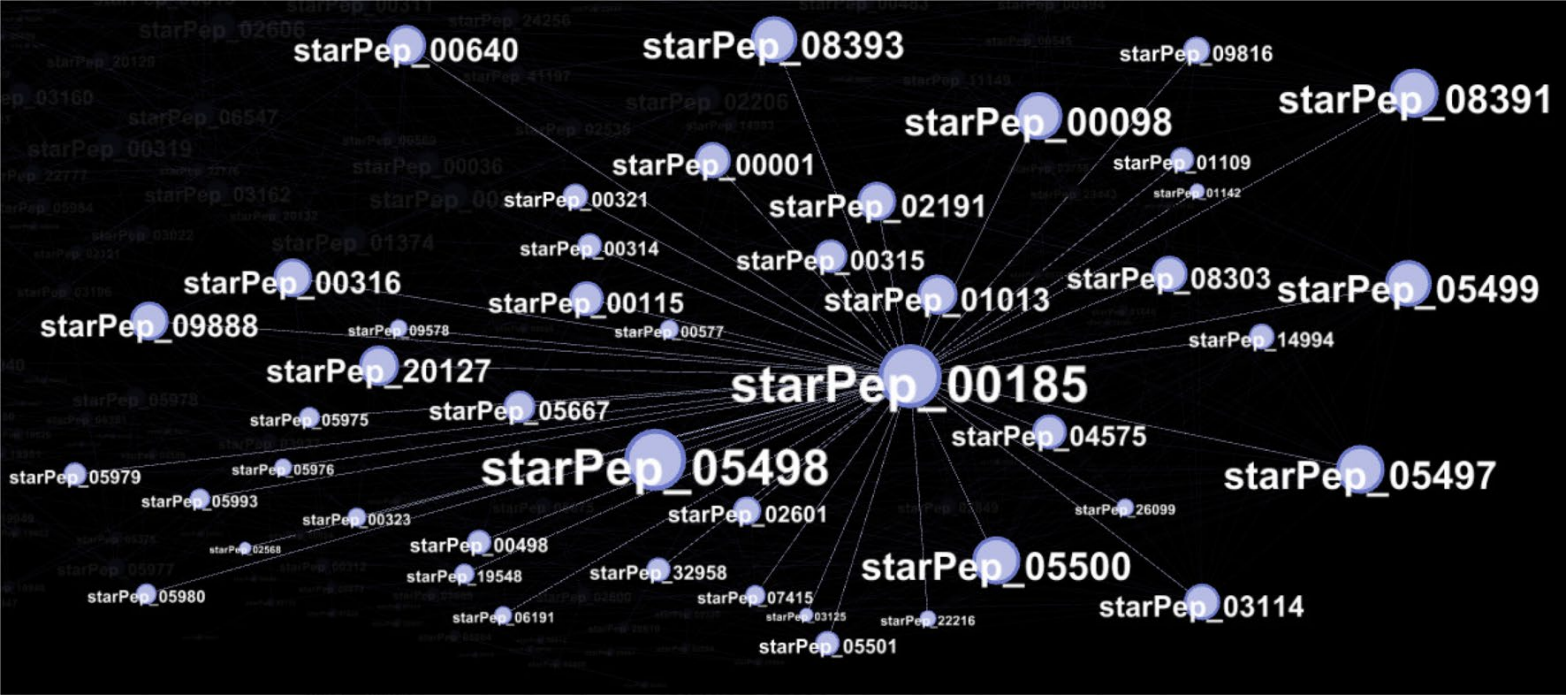
A zoom into the Community 1 for highlighting the most central node (starPep_00185) based on the Hub-Bridge centrality. Nodes are resized according to their local centrality measures. This figure has been created using the software starPep toolbox (version 0.8), available at http://mobiosd-hub.com/starpep/, and PowerPoint 2016 available at https://www.microsoft.com/es-mx/software-download/office.

Of course, nodes inside large communities have higher centrality value and better ranking than those inside smaller groups. Then, we select the top 500 as the representative ones of eight leading communities (Fig. 10b). These local central peptides inside each leading community are given in SI4-3 (FASTA files), and they may be representing sequence fragments or naturally occurring peptides that could be identified as starting points for lead discovery^1,20^. For instance, the peptide starpep_00185 (known as ascaphin-8^44^) is the most central node inside Community 1 (Fig. 11), and some of its peptide neighbors are analogs containing aminoacids substitutions (Table 5). When looking for additional information about this peptide (SI4-2), we noticed that ascaphin-8 is the naturally occurring one, while chemically modified peptide analogs may have greater therapeutic potential^45,46^.

**Table 5:** A family of central peptides inside community 1.

ID^a^	Sequence^b^	Length
starPep_00185	GFKDLLKGAAKALVKTVLF	19
starPep_05498	GFKDLLKGAAKALVKAVLF	19
starPep_05497	GFKDLLKGAAKALKKTVLF	19
starPep_05500	GFKDLLKGAKKALVKTVLF	19
starPep_03114	GFKKLLKGAAKALVKTVLF	19
starPep_05499	GFKDLLKGAAKALVKTVKF	19

^a^ID of the peptides in starPepDB^7^.

^b^Additional information can be found in SI4-2.

Ascaphin-8 is a 19-mer peptide containing a C-terminally α-amidated residue (GFKDLLKGAAKALVKTVLF.NH2). It is one out of eight structurally related antimicrobial peptides, termed ascaphin 1-8, that were originally isolated from the skin secretions of the tailed frog Ascaphus truei^44^. Among the eight purified peptides, ascaphin-8 was the most active compound against a range of pathogenic microorganisms, but it also had limited therapeutic potential due to the greatest hemolytic activity^44^. However, its lysine-substituted analogs showed very low hemolytic activity while displaying potent antibacterial activity against a range of clinical isolates of extended-spectrum β-lactamase producing bacteria^46^. In addition to the broad-spectrum antimicrobial activity, the anticancer property of ascaphin-8 and its analogs has been tested on human hepatoma-derived cells (HepG2), where analogs showed enhanced cytotoxicity to the HepG2 cells and reduced toxicities against mammalian cells when compared with ascaphin-8^45^. Furthermore, other studies^47–49^ revealed that ascaphin-8 may be considered as a starting point for Lead Discovery.

#### Case study II: finding central but non-redundant peptides

As can be observed in Table 5, some neighbor nodes in the interior of communities may be representing a family of similar peptides. Another example of closely related structures can be seen in all 50 members of the Community 5 (see Comm_5 in Fig.10b). The peptides inside this community have the same sequence length of 10 aa. Also, the same amino acid residues compose those 50 peptides, with two amino acids fixed at positions 6 and 10 (see SI4-3 for more sequence details). Therefore, in general, it is expected that there are many nodes with similar centrality values in the network, and it may be better to extract some non-redundant nodes from communities than just selecting the highest-ranked ones.

**Figure 12:**
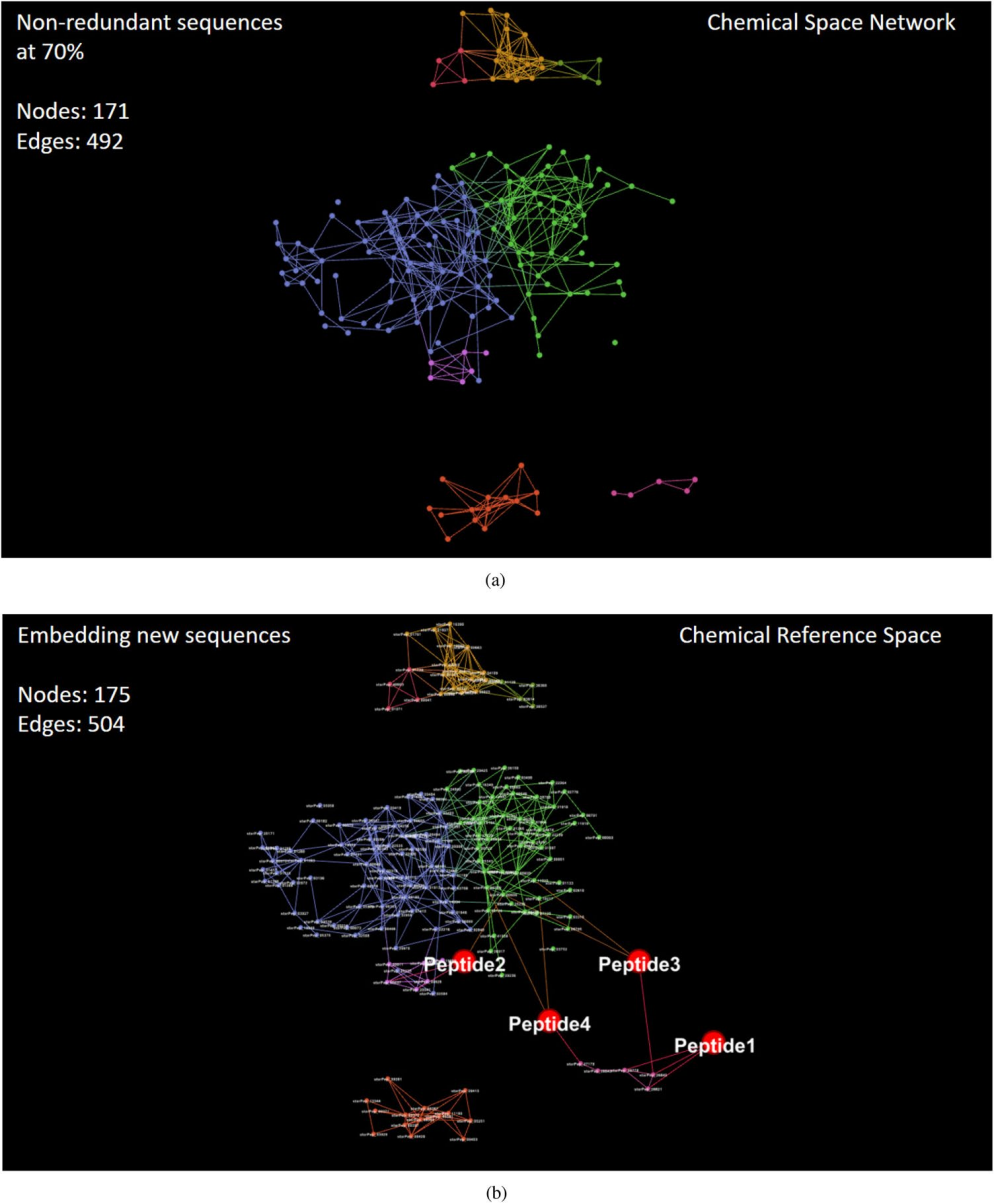
Visualizing the (**a**) subnetwork of anticancer peptides having central but non-redundant structures at 70% of sequence identity, and (**b**) the projection into this network of four in-silico designed anticancer peptides (Peptide1–4)^50^. This figure has been created using the software starPep toolbox (version 0.8), available at http://mobiosd-hub.com/starpep/, and PowerPoint 2016 available at https://www.microsoft.com/es-mx/software-download/office.

Next, to clearly extract central but non-redundant peptides from each community in Fig. 10b, we first sort nodes according to the decreasing order of their local centrality values. Following that order, the redundant sequences are removed at a given percentage of sequence identity. The resulting subnetwork is presented in Fig. 12a. We have used 70% of sequence identity to consider that a particular sequence is related to an already selected central peptide and, as a consequence, removed from the network. For these sequence comparisons, we also applied the Smith–Waterman algorithm^30^ implemented in BioJava^31^, with the BLOSUM62 substitution matrix. Finally, we ranked the non-redundant peptides according to their decreasing values of Harmonic centrality measure (Eq. 12). The sorted list is given in SI4-4 (FASTA file), and the topmost ranked peptides are those having relatively small similarity paths to all other nodes in the network.

#### Case study III: embedding new sequences into a network model

Projecting new sequences into similarity networks of known bioactive peptides may serve to identify the region in a chemical reference space where the new compounds reside. Firstly, the currently selected descriptors are computed for the new peptides to be inserted. Then, each projected peptide is connected to its k nearest neighbors in the defined metric space for further analysis. To illustrate this process, we have embedded four in-silico designed anticancer peptides^50^ in the molecular similarity network under study: Peptide1 (WLFKFLAWKKK), Peptide2 (FPKLLLKFLRLG), Peptide3 (KKFALKLFWWK), and Peptide4 (RLLRRLRIRG).

**Figure 13:**
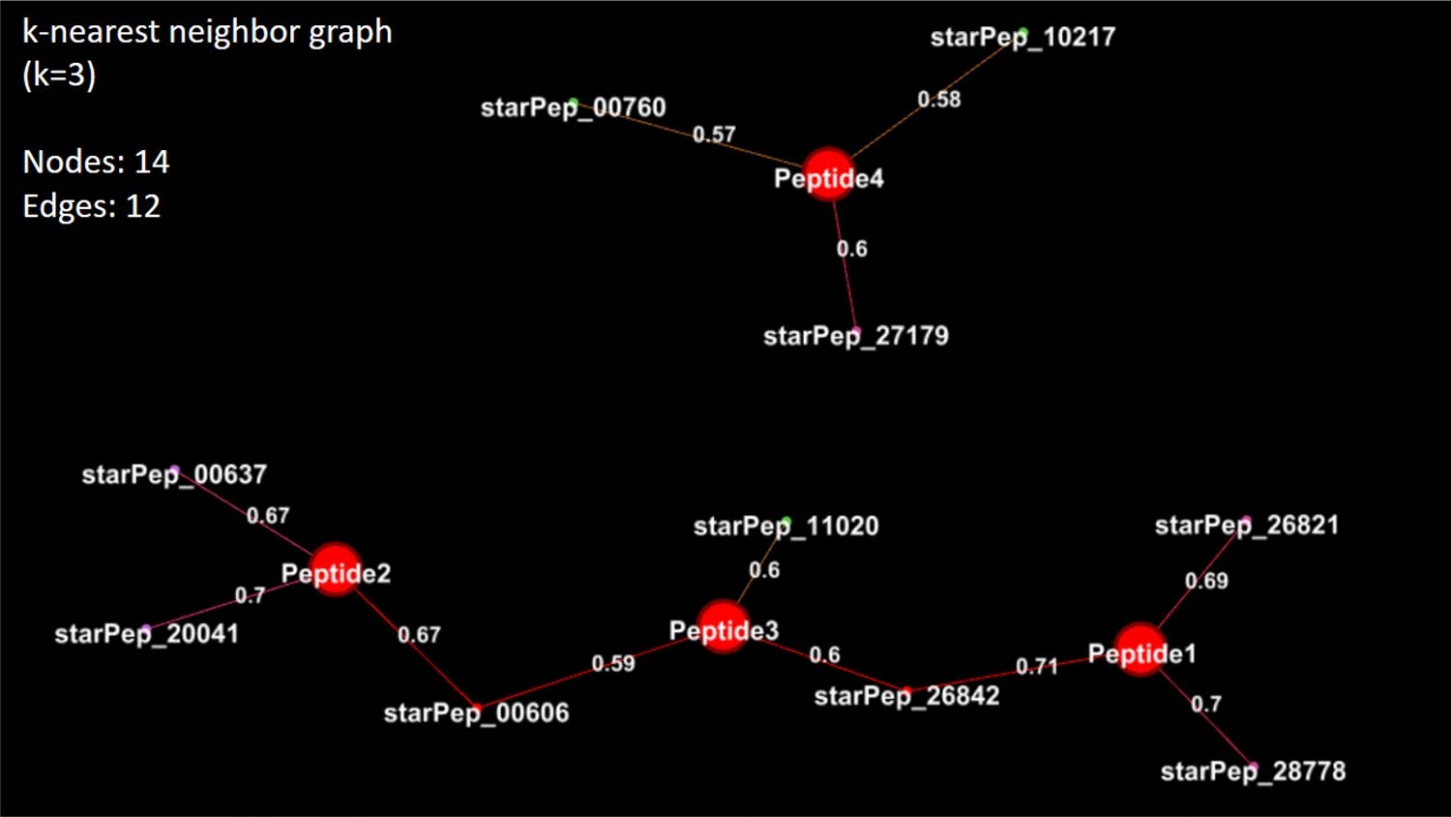
Visualizing the *K*-Nearest Neighbor Graph ($k =3$) of the new compounds of interest (Peptide1–4)^50^ and the recovered peptides (neighbor nodes) from a chemical reference space. Additional information for the recovered peptides can be found in SI4-2. This figure has been created using the software starPep toolbox (version 0.8), available at http://mobiosd-hub.com/starpep/, and PowerPoint 2016 available at https://www.microsoft.com/es-mx/software-download/office.

As can be seen in Fig.  12b, Peptide1, and Peptide2 are occupying different positions in a chemical reference space of anticancer peptides. According to Ref.^50^, these two experimentally verified peptides were the most active peptides, and molecular dynamics simulations suggest that they have different interaction mechanisms with heterogenous POPC/POPS lipid bilayer membranes. Whereas the Peptide1 remains adsorbed to the membrane surface, the Peptide2 has membrane penetration capability^50^.

Furthermore, Fig. 13 reveals the *K*-Nearest Neighbor Graph (*K*-NNG), with $k =3$, based on the Euclidean distances in the defined descriptor space. It can be observed that Peptide3, which is an active compound too, is lying on a similarity path between Peptide1 and Peptide2. Indeed, Peptide3 is more similar to Peptide1 by the length of their similarity path, and less similar to Peptide2, which is expected since Peptide3 and Peptide1 are analogs of the same peptide^50^. However, despite Peptide2 and Peptide4 are having a common origin, Peptide4 is inactive^50^, and it is not connected to the previous ones in the *K*-NNG. Therefore, this retrospective analysis points out that the approach presented here may be useful for getting insight into the chemical space of BPs.

## Conclusion

Here, we have designed and implemented a workflow that transforms automatically raw data of amino acid sequences into a meaningful graph-based representation of chemical space, such as a molecular similarity network. The proposed workflow takes peptide sequences as input to project all these *n* compounds into an *m*-dimensional descriptor space, where both *n* and *m* are natural numbers with $n>> m$. To project each peptide sequence, we calculate molecular descriptors by applying statistical and aggregation operators on amino acid property vectors. Then, using the concepts of entropy and mutual information, an optimized subset of the original features is automatically selected for removing the irrelevant and redundant ones. This allows reducing dimensionality to avoid dealing with high-density networks and increasing efficiency in the definition of descriptor space. We also have conducted experiments for learning and tuning the fully automatic construction of similarity networks from raw data of known bioactive peptides. Our experimental results showed the efficacy of our approach for supporting visual graph mining of the chemical space occupied by a comprehensive collection of bioactive peptides. To illustrate this mining task, we applied a systematic procedure based on network centrality analysis for navigating and mining a biologically relevant chemical space known to date. Therefore, we hope to encourage researchers to use our approach for turning bioactive peptide data into similarity networks into information that could be used in future studies.

## Methods

This section firstly describes how to define a multi-dimensional descriptor space from the amino acid sequences of BPs, which involves molecular descriptors calculation and unsupervised feature selection method. The second part of the section is about how to generate a similarity network from the defined descriptor space, and consequently, how to understand the generated networks through clustering and network science techniques^40^.

### Defining a multi-dimensional descriptor space

The molecular features to be calculated from the peptide sequences may account for physicochemical properties and information about structural fragments or molecular topology^51^. To this end, we extend our in-house Java library^7^ to compute hundreds or thousands of proposed MDs (Tables SI1-1 and SI1-2). The MDs described in Table SI1-1 are legacy descriptors accounting for physicochemical properties, and they were formerly implemented by reusing free software packages to carry out previous studies^52,53^. Unlike the legacy descriptors, new descriptors (those in Table SI1-2) are implemented by applying statistical and aggregation operators on amino acid property vectors, e.g., measures of central tendency, statistical dispersion, OWA operators^54,55^, and fuzzy Choquet integral operators^56,57^. Further, it should be noted that the reasons for using these operators in the calculation of MDs have been demonstrated elsewhere^58–62^.

Let $\mathscr {D}=[x_{ij}]_{n \times m}$ be a descriptor matrix whose rows and columns represent peptide instances and calculated features, respectively, i.e., $x_{ij}$ encodes the numerical value for the *jth* descriptor of the *ith* peptide sequence. Then, denoting the feature *j* as $f_j=\mathscr {D}^{(j)}$, which is the *jth* column of matrix $\mathscr {D}$, an optimization method for feature selection is aiming to identify the best subset of features $F^{*}=\{ f_j \mid j \in I \subseteq \{ 1,2\ldots m\}\}$ among all possible subsets. Nonetheless, finding the optimal subset $F^{*}$ is a hard goal to achieve since the assessment of all possible subsets is not feasible (there are $2^m$ subsets, where *m* is the number of original features), and no efficient algorithm is known that solves this problem. Accordingly, to be able to formulate and solve an optimization problem for subset selection, we first need to introduce some basic definitions for analyzing the relevance and redundancy of features.

#### Basic definitions

**Figure 14:**
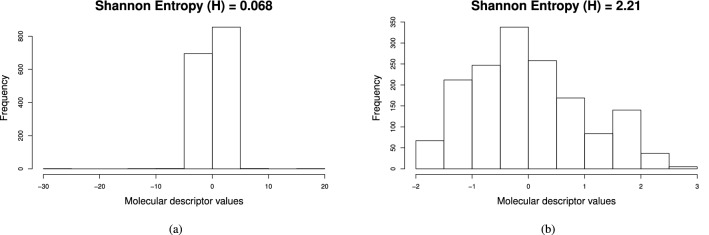
Shannon entropy calculated for two molecular descriptors using the bining scheme of histograms. (**a**) Shannon entropy (H) = 0.068; and, (**b**) Shannon entropy (H) = 2.21. This figure has been created using the R software package (version 3.5.1), available at https://cran.r-project.org/.

*Shannon’s entropy*^63^. Entropy can be considered as one criterion for measuring relevance in the field of unsupervised feature selection^64,65^. A particular calculation of entropy has been proposed in Ref.^23^ to capture the information content of descriptor distributions represented in histograms. For instance, Fig. 14 shows the histograms generated for two molecular descriptors and their information contents. The histograms were constructed by dividing the data range of each variable into the same number of data intervals (bins). Using this binning scheme is possible to transform continuous features into discrete variables that count the number of feature values per bins. Note that the definition of entropy for continuous variables is hard to compute^66^. On this basis, a discretization mechanism can be applied to compute the entropy measure as follows.

Suppose that for each feature, its range is divided into the same number of bins $n_b$. Then, new data representation is generated looking at the peptides that adopt a value located at bin *i* for each feature. Let $S_{i}^{j}$ be the set of peptides whose value fall in the bin *i* for feature *j*, the frequentist probability $p^{j}(i)$ of finding a value within a specific data range *i* for feature *j* is calculated by dividing the data count $|S_{i}^{j}|$ by the summed count of all bins:

1 $$\begin{aligned} p^{j}(i) = \frac{|S_{i}^{j}|}{\sum _{i=1}^{n} |S_{i}^{j}|} \end{aligned}$$

This frequentist probability $p^{j}(i)$ is used to compute the entropy *H* of feature *j*:

2 $$\begin{aligned} H(f_j) = -\sum _{i=1}^{n_b} p^{j}(i) \log p^{j}(i) \end{aligned}$$

As an upper bound, the maximum entropy $H_{max}=\log n_b$ is reached when all data intervals are equally populated, i.e., each bin is occupied by the same amount of peptides. By contrast, the minimum entropy ($H_{min}=0$) is reached if all feature values fall within a single bin *i*. In general, broad histograms result in higher entropy values than narrow histograms, as can be seen in Fig. 14. Therefore, a descriptor with high entropy near to the maximum value means uniform distribution of feature values and should be retained. Conversely, a descriptor with low entropy near to the minimum value is having low information content and should be removed.

**Figure 15:**
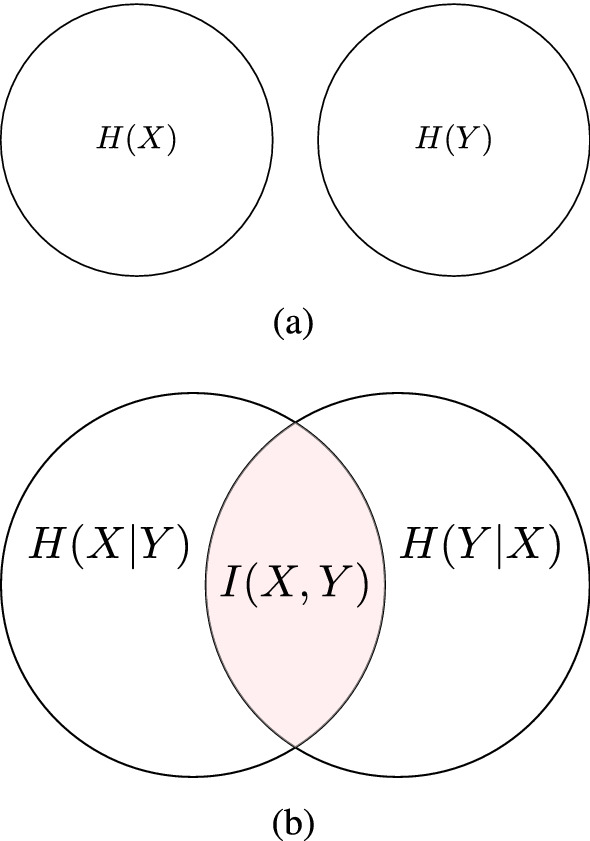
This figure depicts the relationship between entropy $H(\cdot )$ and mutual information $I(\cdot ,\cdot )$ for two variables *X* and *Y*. *I*(*X*, *Y*) measures the amount of information content that one variable contains about another. (a) *I*(*X*, *Y*) is equal to zero if and only if *X* and *Y* are statistically independent; and, (b) $I(X,Y) = H(X) - H(X|Y) = H(Y) - H(Y|X)$, which corresponds to the reduction in the entropy of one variable due to the knowledge of the other. Hence, the *I*(*X*, *Y*) can take values in the interval: $0 \le I(X,Y) \le \min \{H(X), H(Y)\}$; the larger the value of *I*(*X*, *Y*) is, the more the two variables are related. This figure has been created using the package TikZ (version 0.9f 2018-11-19) in Latex, available at https://www.ctan.org/pkg/tikz-cd.

*Mutual information*^66^. Considering two discrete random variables *X* and *Y* with a joint probability *p*(*x*, *y*), the mutual information *I*(*X*, *Y*) is a measure of the dependency between them, and can be used as redundancy criterion between two molecular descriptors (Fig. 15)^67^. Let $S_{i}^{j} \cap S_{l}^{k}$ be the intersection set containing those peptides whose values fall in the bins *i* and *l*, for features *j* and *k*, respectively. The joint probability $p^{j,k}(i,l)$ of observing at the same time two values falling in the bin *i* and *l* is estimated as:

3 $$\begin{aligned} p^{j,k}(i,l) = \frac{|S_{i}^{j} \cap S_{l}^{k}|}{\sum _{r} \sum _{s} |S_{r}^{j} \cap S_{s}^{k}| } \end{aligned}$$

Then, using the marginal and joint probabilities defined in Eqs. 1 and 3, respectively, we computed the mutual information between two features:

4 $$\begin{aligned} I(f_j, f_k) = \sum _{i=1}^{n_b}\sum _{l=1}^{n_b}{p^{j,k}(i,l)\log \frac{p^{j,k}(i,l)}{p^{j}(i) p^{j}(l)}} \end{aligned}$$

In addition to the mutual information criterion, a correlation coefficient is another measure for assessing the redundancy between variables^68^.

*Correlation-based measures*^69^. Pearson correlation coefficient ($\rho$) is a well-known criterion for measuring the linear association of a pair of variables *X* and *Y*^69^. This correlation coefficient as a criterion of redundancy between two features is given by

5 $$\begin{aligned} \rho (f_j, f_k) = \frac{\sum _{i=1}^{n}(x_{ij}-\bar{x_{j}})(x_{ik}-\bar{x_{k}})}{\sqrt{\sum _{i=1}^{n}(x_{ij}-\bar{x_{j}})^{2}}\sqrt{\sum _{i=1}^{n}(x_{ik}-\bar{x_{k}})^{2}}} \end{aligned}$$

where $x_{ij}$ and $\bar{x_{j}}$ indicate, respectively, the *i*th value and the mean of the feature $f_j$. We used the absolute value $| \rho (f_j, f_k) |$ since the sign of the coefficient only indicates that, in case of positive sign, both feature values tend to increase together or, in case of negative sign, the values of one feature increase as the values of the other feature decrease. If two features have an exact linear dependency relationship, $|\rho (f_j, f_k) |$ is 1; if two features are totally independent, $| \rho (f_j, f_k) |$ is 0; whereas larger values in the range [0, 1] indicate higher linear correlation. However, this correlation coefficient is not suitable to capture the dependency between two variables that is not linear in nature.

In addition to the Pearson correlation coefficient, Spearman’s rank correlation coefficient ($r_s$) measures the monotonic relationship (whether linear or not) for a pair of variables *X* and *Y*^69^. This correlation coefficient $r_s$ relies on the rank order of values for each variable instead of the value itself, i.e., it assesses the similarity between the two rankings *rank*(*X*) and *rank*(*Y*) rather than comparing raw values of *X* and *Y*, where *rank*() denotes the data transformation in which original values are replaced by their position when the data are sorted. The formula for Spearman’s coefficient is based on Pearson’s correlation ($\rho$) with the distinction of being computed on ranks:

6 $$\begin{aligned} r_s(f_j, f_k) = \rho (rank(f_j), rank(f_k)) \end{aligned}$$

We also used the absolute value of the monotonic correlation coefficient $|r_s(f_j, f_k)|$ as a criterion of similarity between two features. If one feature is a perfect monotone function of another, then $|r_s(f_j, f_k)|$ is 1; if they are totally uncorrelated, $|r_s(f_j, f_k)|$ is 0; otherwise, $|r_s(f_j, f_k)|$ lies in the range [0,1] and it is high when the values of two features have a rank correlation, and low when values have a dissimilar rank.

#### Unsupervised feature selection

Here we present a two-stage procedure for solving a feature subset selection problem. In the first stage, a candidate feature subset is selected by removing irrelevant and redundant features from the original set (Algorithm SI2-1). At this stage, descriptors are ranked in descending order of their entropy values to choose the top-ranked ones or those having entropy values greater than a given threshold. In this way, relevant features are progressively selected as long as they are not correlated to any of the already selected ones. Moreover, an assessment helped to decide whether two features are correlated or not, since either Pearson’s or Spearman’s coefficient may be used with different correlation thresholds.

The effect of changing both the correlation method and cutoff value was analyzed in an experimental study. We used a Procrustes analysis^29^ to quantitatively assess the quality of retained features in the candidate subset for representing the original ones^70,71^. This analysis consists of measuring proximities between the original and reduced descriptor spaces describing the same set of peptides. So the optimal transformation should be applied to one feature space based on scaling, rotations, and reflections, to minimize the sum of squared errors as a measure of fit (goodness-of-fit criterion). Since the original feature space and the reduced space are having different dimensions, they can be properly comparable by using the same amount of principal components^13^. Therefore, the goodness-of-fit criterion equals to 0 may indicate that the two descriptor spaces coincide. On the contrary, a goodness-of-fit value equals to 1 means that the two descriptor spaces are utterly dissimilar.

In the second stage, the aim is to find the best subset containing the most informative and less redundant features from the candidate set. Indeed, the number of previously selected features can be further reduced by finding a solution to an optimization problem. We define the problem to solve as

7 $$\mathop {{\text{Maximize}}}\limits_{F \in \Omega } \;\;\Phi \left( F \right) = \frac{1}{{\left| F \right|}}\sum\limits_{{f_j} \in F} {H\left( {{f_j}} \right)} - \frac{1}{{{{\left| F \right|}^2}}}\sum\limits_{{f_{j,}}{f_k} \in F} {I\left( {{f_j},{f_k}} \right)}$$

where $\Phi (F)$ is the objective function, and *F* is a subset of features over the search space $\Omega$ of all possible subsets from the candidate feature set. By looking more deeply into the objective function, one can see that $\Phi (F)$ is defined as a subtraction between the average of entropy values and the average of normalized mutual information as a penalization term. Maximizing this subtraction expression is a simple form of maximizing the first term as a measure of relevance, while minimizing the second term as a measure of redundancy^67^.

Our definition of $\Phi (F)$ is based on a previous work^67^, where the class label information is available. In our case of unsupervised feature selection, no class labels are available. Thus, we modify the previous definition in Ref.^67^ to satisfy our needs. Additionally, we have incorporated the ranking and filtering stage for an early removing of irrelevant and redundant features. In that early stage, both the features with low entropy values affecting the average in the first term of $\Phi (F)$, and those with high redundancy in the second term of the subtraction are removed.

Despite an early removal of useless and correlated features, an exhaustive search over all possible subsets of the candidate feature set is computationally unaffordable. Thus, it is possible to use a heuristic search strategy that may give good results, although there is not guarantee of finding the optimal subset. We adopt a greedy hill-climbing procedure (Algorithm SI2-2) that starts with the full set of candidate features and eliminates them progressively (backward elimination^64^). This procedure traverses the search space by considering all possible single feature deletions at a given set, and picking the subset with the highest evaluation according to the objective function $\Phi (F)$.

### Translating a descriptor space into similarity networks

After calculating and selecting MDs, the workflow stage is the translation of the molecular descriptor space into similarity networks. Assuming no distinction between a node and the peptide it represents, the similarity network is constituted by a set of nodes that are characterized by feature vectors (descriptors) in a metric space. Then, the Euclidean distance *d*(*u*, *v*) between two nodes *u*, *v* can be transformed into a pairwise similarity measure $sim(u,v) \in [0,1]$ by using the following formula:

8 $$\begin{aligned} sim(u,v) = 1 - \frac{d(u,v)}{\max _{p,q \in V}d(p,q)} \end{aligned}$$

As it was suggested in Ref.^41^, the definition of *sim*(*u*, *v*) is suitable to construct a similarity network: there is an edge $<u,v> \in E$ between nodes *u* and *v* if their pairwise similarity value is equal or greater than a given threshold *t*, i.e., if $sim(u,v) \ge t$. In our case, we considered these networks as weighted graphs. That is, for a given threshold *t* the similarity matrix $\mathscr {S_M}=[s_{ij}]_{n \times n}$ that stores the similarity values ($0 \le s_{ij} \le 1$) of every pair of peptides becomes an adjacency matrix $\mathscr {A}=[a_{ij}]_{n \times n}$ whose values are given by

9 $$\begin{aligned} a_{ij} = \left\{ \begin{array}{c l} s_{ij} &{} \text{ if } i \ne j, s_{ij} \ge t \\ 0 &{} otherwise. \end{array} \right. \end{aligned}$$

**Figure 16:**
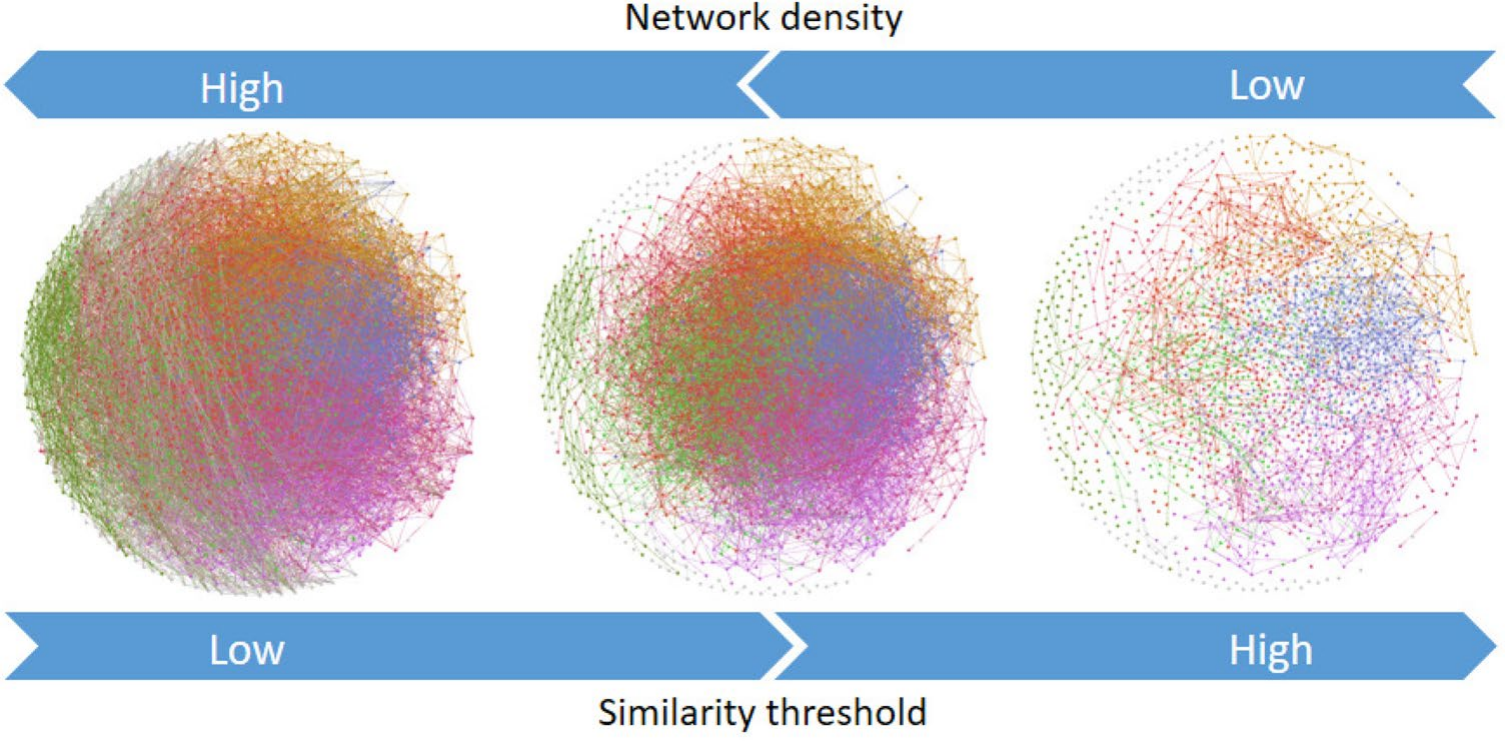
Network density at varying similarity thresholds. This figure has been created using the software starPep toolbox (version 0.8), available at http://mobiosd-hub.com/starpep/, and PowerPoint 2016 available at https://www.microsoft.com/es-mx/software-download/office.

Keeping in mind that the perception of similarity is in the eyes of the beholder^72^, we considered that the threshold $t \in [0,1]$ is a user-defined parameter to be explored. Besides, a predefined constant value for *t* is not useful in practice because the distribution of similarity values may strongly vary depending on the input dataset. Note that when the threshold *t* is modified, the number of edges might change, whereas the number of nodes remains unchanged. Thus, some network properties can be altered as a function of the parameter *t*, e.g., network density (Fig. 16).

Network density is defined as the ratio between the number of edges and the total number of possible relationships:

10 $$\begin{aligned} network\_density = \frac{2m}{n (n-1)} \end{aligned}$$

where $m=|E|$, and $n=|V|$. In the extreme case where threshold $t=0$, all compounds are considered similar, and the complete network with $n (n-1)/2$ edges would be drawn ($network\_density=1$). On the contrary, when $t=1$, a minimally connected network is realized ($network\_density \text{ is } \text{ almost, } \text{ if } \text{ not } 0$). In general, when *t* increases the network density tends to decrease (Fig. 16).

Of course, the task of understanding what networks are telling us depends on a chosen value of *t*. It may become more complicated at low threshold values yielding densely connected nodes, since a picture with too many lines will be difficult for human eye perception. Networks cannot be readily interpretable at high levels of their density values. Moreover, large networks with thousands of nodes and millions of possible relationships may require high memory usage. It may cause out of memory errors when computing and loading the graph into the RAM of personal computers.

A straightforward way of achieving low levels of density is using a high value of threshold *t* (Fig. 16). At large values of *t*, the similarity networks can be readily interpretable^41^. Nonetheless, increasing too much this value may give rise to edgeless networks, either disconnecting many nodes or isolating groups of them. Therefore, the parameter *t* should be handled carefully to achieve interpretable graphs without losing information from the network topology. Setting an inappropriate value for the threshold *t* may be yielding networks where the topological information is hidden^15^.

#### Half-space proximal networks

When constructing a similarity network for a large dataset of BPs (ten of thousands of them), the amount of RAM required is very high to store the matrix $\mathscr {S_M}=[s_{ij}]_{n \times n}$ to be pruned. Hence, what is needed here is the creation of a sparse network with similarity/distance properties near to that of the complete graph, but where only a small fraction of the possible maximum number of links among nodes are used. One such network is achieved by the Half-Space Proximal (HSP) test^35^, which is a simple algorithm that may be applied to build similarity networks with lower-density levels than CSNs. The HSP test extracts a connected network over a set of points in a metric space, in our case, the multi-dimensional descriptor space, and it works as follows.

**Figure 17:**
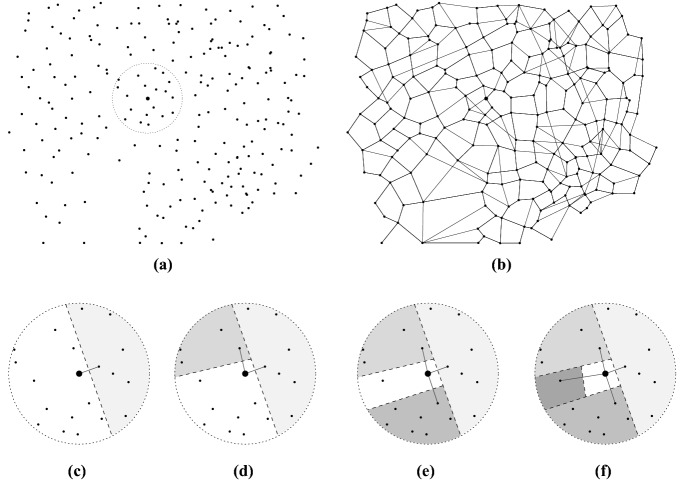
Applying the HSP test for extracting neighbors of a given point: (**a**) Initial configuration; (**b**) Final configuration; (**c**) 1st neighbor; (**d**) 2nd neighbor; (**e**) 3rd neighbor; and, (**f**) 4th neighbor. The above image is a modified version of Fig. 1 in Ref.^35^. This figure has been created using the software IPE (version 7.2.20), available at http://ipe.otfried.org/.

Assuming for simplicity a two-dimensional (2D) space, Fig. 17 illustrates how the HSP test is applied to an arbitrary set of points. For an initial point *u*, its nearest neighbor *v* is taken to add an edge between them. Then, the 2D space is divided into two half-planes by an imaginary perpendicular line passing through the midpoint of the edge connecting *u* and *v*. Notice in Fig. 17 that one half-plane is the region of points (the shaded ones) closer to *v* than to *u*, and this region is called the forbidden area for the point *u*. Next, from the remaining candidate points that do not belong to the forbidden area, the nearest point is taken to be the new neighbor of *u*. These steps are repeated until the candidate set of points is empty.

The procedure described above can be independently applied to each point in the metric space. Thus, we made a parallel implementation of the HSP test (Algorithm SI2-3). The resulting relationships between nodes and their HSP neighbors comprise the HSP network called in short as HSPNs. These HSPNs $G^{'}=(V, E^{'}, w)$ are subgraphs of the CSNs, denoted by $G=(V, E)$, where $E^{'} \subset E$ for an arbitrary threshold *t*. We also consider $G^{'}$ as a weighted network, where the weight function $w: E^{'} \rightarrow \left[ 0,1 \right]$ is indicating the similarity values (Eq. 8) of connected peptides.

### Understanding the generated similarity networks

So far, we have emphasized on how to build a molecular descriptor space and meaningful network representations describing similarity relationships between peptides. Now, we will suggest how network science techniques can be used to get a deeper insight into the generated similarity networks. In practice, we implement some functionalities based on the open source project Gephi^73^. The main idea we pursue is that researchers may perceive the graph structure and seek nodes occupying important roles within a network of interest.

#### Detecting communities

Clustering is a fundamental technique in unsupervised learning for finding and understanding the hidden structure in the data. This technique consists of separating data elements into several groups, such that elements in the same group are similar and elements in different groups are dissimilar to each other. The resulting groups are called clusters or communities in the case of networks^40^. As such, the usage of clustering to identify densely connected nodes has been known as community detection^74^, and the detected communities may represent various groups of compounds having different chemical properties in the context of a molecular similarity network.

One effective approach for network community detection has been to maximize a quality function called modularity^75^, which is useful for evaluating the goodness of a partition into communities. This modularity measure is the fraction of edges connecting nodes in the same community minus the expected value of the same quantity in a network with identical community structure and degrees of vertices, but where edges are placed randomly. Formally, modularity (*Q*) for a weighted network has been defined as^76^:

11 $$\begin{aligned} Q = \frac{1}{2m}\sum _{ij}{\left( a_{ij}-\frac{k_ik_j}{2m}\right) } \delta (c_i,c_j) \end{aligned}$$

where $a_{ij}$ is the weight of the edge (in our case the similarity value) between nodes *i* and *j* or 0 if there is no such connection, $m=\frac{1}{2}\sum _{ij}a_{ij}$ is the total sum of weights in the whole network, $k_i=\sum _j{a_{ij}}$ is the sum of weights of the edges attached to node *i*, $c_i$ is the community to which node *i* is assigned, and $\delta (c_i,c_j)$ is 1 if both vertex *i* and *j* are members of the same community ($c_i = c_j$) or 0 otherwise.

The modularity *Q* (Eq. 11) can be either positive or negative, and its maximum value is 1^75^. For maximizing *Q* over possible network divisions, we used the Louvain Clustering algorithm^77^ implemented in Gephi. This algorithm has achieved good results in terms of accuracy and computing time if compared with other available methods in the literature^77,78^. It initially assigns a different community to each node of the network. Then, for each node *u*, it evaluates the gain of modularity that would take place by moving node *u* to the community of its neighbors. The movement of placing node *u* in the community of node *v* is done if the gain achieves the highest positive contribution to the modularity. If no positive gain contribution is achieved, the node *u* stays in its original community. This first phase is repeated for all nodes until no further improvement is possible. Later, in a second phase, the algorithm builds a new network whose nodes are the communities found in the previous phase. Once again, the first phase is reapplied to the new network and stops when there is not a movement that can improve the modularity.

#### Ranking nodes based on centrality measures

Centrality is a concept that has been widely used in another field of sciences, such as social network analysis, to identify influential nodes in a network under study^40,79,80^. Formally, a centrality measure *C*(*u*) is a function that assigns a non-negative real number to each node *u* in the network^40^. Then, a basic analytic task lies in finding those nodes that are more likely to be of interest for drug discovery according to the values of *C*(*u*)^20^. To this end, one may focus on quantifying either a global or local measure of how important a node is in a given network^80^.

Global centrality measures consider the whole network, and of course, they are more costly to compute than local measures using only neighborhood information^80^. For instance, Harmonic Centrality^81^ is one distance-based centrality measure deemed to be global. This centrality for a node *i* is defined as

12 $$\begin{aligned} C_{H}(i) = \sum _{j \ne i} \frac{1}{d_g(i,j)} \end{aligned}$$

where the geodesic distance $d_g(i,j)$ is the length of the shortest path from *i* to *j*. At considering that the shortest path length is calculated using Dijkstra’s algorithm, by convention, $d_g(i,j)=\infty$ and $1/\infty =0$ if there is no such path when dealing with unreachable nodes in disconnected networks. That means that the worst-case complexity is quadratic in the number of nodes, which may be inefficient for many large networks^82^.

In contrast to global measures, local centrality measures are only based on information around nodes^80^, and they are shown to be useful by exploiting the community structure. Indeed, it is reasonable to find network communities to identify which are the most influential nodes by analyzing the detected communities. Although sometimes another strategy is required to capture information based on locally available network structures^80^. Nodes may play a role within a network depending on their positions in the community to which they belong. For instance, nodes are local hubs if they are connecting many internal nodes in their own communities, while those at the boundary may act as bridges between groups. On the one hand, local hubs may represent local central molecules in the chemical space of BPs^20^. On the other hand, nodes connecting communities may represent intermediate structures between groups of similar peptides^20,83^.

By considering a network with community structure, the number of both intra- and inter-community links attached to a node can be useful to identify those nodes acting as hubs or bridges. So, the internal and external connectivity strength for each node *i* in its community $c_i$ is measured by using the formula:

- Internal strength: $$k_i^{int} = \sum _{j \in c_i}{a_{i,j}}$$
- External strength: $$k_i^{ext} = \sum _{j \notin c_i}{a_{i,j}}$$

where $a_{i,j}$ contains the weight of the edge (*i*, *j*), i.e., the similarity value between nodes *i* and *j*, or 0 if there is no such edge.

It should be noted that, for an unweighted network, the value of $a_{i,j}$ would be 1 if nodes *i* and *j* are adjacent, or 0 otherwise. Thus, in the unweighted counterpart, the internal strength $k_i^{int}$ would be analogous to the number of neighbors of node *i* that belong to the same community $c_i$, whereas the external strength $k_i^{ext}$ would be the number of neighbors that do not belong to the same community. Following this analogy, the total strength $k_i^{int} + k_i^{ext}$ would be the degree of node *i*.

Based on the above idea, both $k_i^{int}$ and $k_i^{ext}$ can also be combined to compute a recently proposed centrality measure, called Community Hub-Bridge centrality^84^:

13 $$\begin{aligned} C_{HB}(i) = k_i^{int} * card(c_i) + k_i^{ext} * nnc(i) \end{aligned}$$

where $card(c_i)$ is the size of the community to which the node *i* belongs, and *nnc*(*i*) is the number of neighboring communities that a node *i* can reach by its inter-community links. This centrality measure is intended to identify preferentially nodes acting as hubs inside large communities and bridges targetting various communities.

## Supplementary information

Supplementary Legends.

Supplementary Information 1.

Supplementary Information 2.

Supplementary Information 3.

Supplementary Information 4.

Supplementary Information 5.

Supplementary Information 6.

Supplementary Information 7.

Supplementary Information 8.

Supplementary Information 9.
