# Supplementary material for: Automatic construction of molecular similarity networks for visual graph mining in chemical space of bioactive peptides: an unsupervised learning approach
Source: Sci Rep. 2020 Oct 22;10:18074. doi: 10.1038/s41598-020-75029-1 (PMC7583304; doi:10.1038/s41598-020-75029-1)
Supplement: Supplementary file 3 — Supplementary Information 2. [file 41598_2020_75029_MOESM3_ESM.docx]

**Supporting Information:**

Automatic construction of similarity networks for visual graph mining in chemical space of bioactive peptides: an unsupervised learning approach

Longendri Aguilera-Mendoza, Yovani Marrero-Ponce*, César R. García-Jacas, Edgar Chavez, Jesus A. Beltran, Hugo A. Guillen-Ramirez, Carlos A. Brizuela*.

Corresponding authors *: Y. Marrero-Ponce: ymarrero@usfq.edu.ec or ymarrero77@yahoo.es; Carlos A. Brizuela: cbrizuel@cicese.edu.mx

**Contents**: Peptide sequences (FASTA format) that were used for calculating and comparison of the proposed molecular descriptors.

>starPep_00021

ACYCRIPACIAGERRYGTCIYQGRLWAFCC

>starPep_00022

ALWKNMLKGIGKLAGKAALGAVKKLVGAES

>starPep_00048

DCYCRIPACIAGERRYGTCIYQGRLWAFCC

>starPep_00070

GIPCGESCVWIPCISAALGCSCKNKVCYRN

>starPep_00140

GWKDWAKKAGGWLKKKGPGMAKAALKAAMQ

>starPep_00141

GWKDWLNKGKEWLKKKGPGIMKAALKAATQ

>starPep_00160

ACYCRIPACLAGERRYGTCFYMGRVWAFCC

>starPep_00163

ALWKNMLKGIGKLAGQAALGAVKTLVGAES

>starPep_00216

ILQKAVLDCLKAAGSSLSKAAITAIYNKIT

>starPep_00293

GFPCGESCVFIPCISAAIGCSCKNKVCYRN

>starPep_00306

GIPCGESCVFIPCLTTVAGCSCKNKVCYRN

>starPep_00307

GIPCGESCVWIPCISSAIGCSCKSKVCYRN

>starPep_00331

GLKDWVKIAGGWLKKKGPGILKAAMAAATQ

>starPep_00348

GWKDWLKKGKEWLKAKGPGIVKAALQAATQ

>starPep_00358

KIPCGESCVWIPCLTSVFNCKCENKVCYHD

>starPep_00398

ACYCRIPACFAGERRYGTCFYLGRVWAFCC

>starPep_00406

ASIIKTTIKVSKAVCKTLTCICTGSCSNCK

>starPep_00410

AYPGNGVHCGKYSCTVDKQTAIGNIGNNAA

>starPep_00440

GAIKDALKGAAKTVAVELLKKAQCKLEKTC

>starPep_00463

GIPCGESCVFIPCITSVAGCSCKSKVCYRN

>starPep_00558

VGECVRGRCPSGMCCSQFGYCGKGPKYCGR

>starPep_00645

FVYGNGVTSILVQAQFLVNGQRRFFYTPDK

>starPep_00683

GIPCAESCVWIPCTVTALVGCSCSDKVCYN

>starPep_00684

GIRCPKSWKCKAFKQRVLKRLLAMLRQHAF

>starPep_00736

GNAACVIGCIGSCVISEGIGSLVGTAFTLG

>starPep_00752

GVPCGESCVFIPCITGVIGCSCSSNVCYLN

>starPep_00795

KFFRKLKKSVKKRAKEFFKKPRVIGVSIPF

>starPep_00797

KIPCGESCVWIPCVTSIFNCKCENKVCYHD

>starPep_00806

KSKEKIGKEFKRIVQRIKDFLRNLVPRTES

>starPep_00902

AGECVQGRCPSGMCCSQFGYCGRGPKYCGR

>starPep_00912

ANFEIVNNCPYTVWAAASPGGGRRLDRGQT

>starPep_00987

GAFGNFLKGVAKKAGLKILSIAQCKLFGTC

>starPep_00988

GAFGNFLKGVAKKAGLKILSIAQCKLSGTC

>starPep_01027

GIPCAESCVWIPCTVTALIGCGCSNKVCYN

>starPep_01028

GIPCAESCVYIPCTVTALLGCSCSNRVCYN

>starPep_01029

GIPCGESCVFIPCITAAIGCSCKSKVCYRN

>starPep_01030

GIPCGESCVFIPCITGAIGCSCKSKVCYRN

>starPep_01031

GIPCGESCVWIPCISAAIGCSCKSKVCYRN

>starPep_01072

GLPVCGETCFTGTCYTNGCTCDPWPVCTRN

>starPep_01094

GVITDALKGAAKTVAAELLRKAHCKLTNSC

>starPep_01223

ACLPNSCVSKGCCCGBSGYWCRQCGIKYTC

>starPep_01227

AEVAPAPAAAAPAKAPKKKAAAKPKKAGPS

>starPep_01232

ALWKTIIKGAGKMIGSLAKNLLGSQAQPES

>starPep_01253

CSTNTFSLSDYWGNKGNWCTATHECMSWCK

>starPep_01254

CSTNTFSLSDYWGNNGAWCTLTHECMAWCK

>starPep_01323

GAFGDLLKGVAKEAGMKLLNMAQCKLSGKC

>starPep_01327

GEFLKCGESCVQGECYTPGCSCDWPICKKN

>starPep_01369

GIPCAESCVWIPCTVTALLGCSCSNNVCYN

>starPep_01370

GIPCGESCVWIPCLTSAIGCSCKSKVCYRN

>starPep_01378

GLFKTLIKGAGKMLGHVAKQFLGSQGQPES

>starPep_01405

GLPTCGETCFGGTCNTPGCTCDPWPVCTHN

>starPep_01408

GLPVCGETCFGGTCNTPGCSCETWPVCSRN

>starPep_01409

GLPVCGETCFGGTCNTPGCTCDPWPVCTRN

>starPep_01435

GTRCGETCFVLPCWSAKFGCYCQKGFCYRN

>starPep_01443

GWFKKAWRKVKNAGRRVLKGVGIHYGVGLI

>starPep_01444

GWFKKTFHKVSHAVKSGIHAGQRGCSALGF

>starPep_01472

IRNSLTCRFNFGICLPKRCPGRMRQIGTCF

>starPep_01519

MKTILRFVAGYDIASHKKKTGGYPWERGKA

>starPep_01601

SLGPAIKATRQVCPKATRFVTVSCKKSDCQ

>starPep_01611

SWFSRTVHNVGNAVRKGIHAGQGVCSGLGL

>starPep_01639

ACYCRIPACLAGERRYGTCFYLGRVWAFCC

>starPep_01640

ACYCRIPACLAGERRYGTCFYRRRVWAFCC

>starPep_01775

GIPCAESCVWIPCTVTALLGCSCSNKVCYN

>starPep_01776

GIPCGESCVFIPCISSVIGCSCSSKVCYRN

>starPep_01777

GIPCGESCVWIPCITSAIGCSCKSKVCYRN

>starPep_01778

GIPCGESCVWIPCLTSAVGCSCKSKVCYRN

>starPep_01779

GIPCGESCVYIPCLTSAIGCSCKSKVCYRN

>starPep_01780

GIPCGESCVYIPCLTSAVGCSCKSKVCYRN

>starPep_01800

GLPVCGETCFGGTCNTPGCACDPWPVCTRD

>starPep_01801

GLPVCGETCFGGTCNTPGCICDPWPVCTRN

>starPep_01802

GLPVCGETCFGGTCNTPGCSCDPWPMCSRN

>starPep_01819

GTPCGESCVWIPCISSAVGCSCKNKVCYKN

>starPep_01823

GVIIDTLKGAAKTVAAELLRKAHCKLTNSC

>starPep_01828

GVPVCGETCFGGTCNTPGCSCDPWPVCSRN

>starPep_01831

GWFKKAWRKVKNAGRVLKGVGIHYGVGLIG

>starPep_01897

KWKKFIKKIGIGAVLKVLTTGLPALKLTKK

>starPep_01974

RCICTTRTCRFPYRRLGTCLFQNRVYTFCC

>starPep_02013

SIPCGESCVWIPCTITALAGCKCKSKVCYN

>starPep_02026

TLRGDERILSILRHQNLLKELQDLALQGAK

>starPep_02050

XXVPYPRPFPRPPIGPRPLPFPGGGRPFQS

>starPep_02058

ACYCRIPACLAGERRYGTCFYLRRVWAFCC

>starPep_02067

ALWKTLLKGAGKVFGHVAKQFLGSQGQPES

>starPep_02075

ATFDIQNKXTYTVWAAAWAPSYPGGXKQLD

>starPep_02099

DSHEERRQGRHGHHEYGRKFHEKHHSHRGY

>starPep_02148

FNRGGYNFGKSVRHVVDAIGSVAGILKSIR

>starPep_02183

GIFLDKLKNFAKGVAQSLLNKASCKLSGQC

>starPep_02197

GIPCAESCVYIPCTITALLGCKCKDQVCYN

>starPep_02198

GIPCAESCVYIPCTITALLGCKCQDKVCYD

>starPep_02199

GIPCGESCVFIPCLTSAIDCSCKSKVCYRN

>starPep_02200

GIPCGESCVWIPCISSAIGCSCKNKVCYRN

>starPep_02222

GLPCGETTCFTGKCYTPGCSCSYPICKKIN

>starPep_02224

GLPTCGETCFGGTCNTPGCSCSSWPICTRD

>starPep_02227

GLPVCGESCFGGTCNTPGCACDPWPVCTRD

>starPep_02450

AISCGQVSSAIGPCLSYARGQGSAPSAGCC

>starPep_02451

AKEFGIPAAVAGTVLNVVEAGGWVTTIVSI

>starPep_02477

CGESCVFIPCITSVAGCSCKSKVCYRNGIP

>starPep_02557

GFFDRIKALTKNVTLELLNTITCKLPVTPP

>starPep_02588

GIPCGESCVFIPCISGVIGCSCKSKVCYRN

>starPep_02590

GIPCGESCVWIPCLTSAIGCSCKSKVCYKD

>starPep_02591

GIPCGESCVWIPCLTSAVGCPCKSKVCYRN

>starPep_02592

GIPCGESCVWIPCLTSTVGCSCKSKVCYRN

>starPep_02593

GIPCGESCVWIPGISAAIGCSCKNKVCYRN

>starPep_02616

GLPVCGETCFGGTCNTPGCSCSSWPICTRN

>starPep_02637

GRADYNFGYGLGRGTRKFFNGIGRWVRKTF

>starPep_02648

GTLPCESCVWIPCISSVVGCSCKSKVCYKN

>starPep_02649

GTPCAESCVYLPCFTGVIGCTCKDKVCYLN

>starPep_02650

GTPCGESCVYIPCFTAVVGCTCKDKVCYLN

>starPep_02651

GTPCGESCVYIPCISGVIGCSCTDKVCYLN

>starPep_02873

SISCGETCTTFNCWIPNCKCNHHDKVCYWN

>starPep_02958

ALWKSLLKGAGQLVGGVVQHFMGSQGQPES

>starPep_02980

CAESCVYIPCTVTALLGCSCSNRVCYNGIP

>starPep_03094

GAFGDLLKGVAKEAGLKLLNMAQCKLSGNC

>starPep_03095

GAFGNLLKGVAKKAGLKILSIAQCKLSGTC

>starPep_03098

GDVHAQTTWPCATVGVSVALCPTTKCTSQC

>starPep_03145

GILLNTLKGAAKNVAGVLLDKLKCKITGGC

>starPep_03149

GILTDTLKGAAKNVAGVLLDKLKCKITGGC

>starPep_03150

GIPCAESCVYIPCTITALLGCSCKNKVCYN

>starPep_03151

GIPCGESCVWIPCISAAIGCSCKNKVCYRN

>starPep_03152

GIPCGESCVWIPCLTSAIGCSCKSKVCYRD

>starPep_03153

GIPCGESCVYIPCTVTALAGCKCKSKVCYN

>starPep_03154

GKCNVLCQLKQKLRSIGSGSHIGSVVLPRG

>starPep_03158

GLAGAISSALDKLKQSQLIKNYAKKLGYPR

>starPep_03175

GLLLDTVKGAAKNVAGILLNKLKCKMTGDC

>starPep_03188

GLPTCGETCFGGTCNTPGCSCSSWPICTRN

>starPep_03189

GLPTCGETCFGGTCNTPGCTCDPWPICTRD

>starPep_03229

GSAIRCGESCLLGKCYTPGCTCDRPICKKN

>starPep_03235

GSVIGCGETCLRGRCYTPGCTCDHGICKKN

>starPep_03238

GTLPCGESCVWIPCISAVGCSCKSKVCYKN

>starPep_03264

GWVACVGACGTVCLASGGVGTEFAAASYFL

>starPep_03315

KFFKRLLKSVRRAVKKFRKKPRLIGLSTLL

>starPep_03556

PPCRGIFCRRVGSSSAIARPGKTLSTFITV

>starPep_03762

ALWKTMLKKAAHVGKHVGKAALGAAARRRK

>starPep_03788

ASAAGAVRAGDDETLLKPVLNSLDNLVSGL

>starPep_03789

ASAAGAVRAGDDETLLNPVLNSLDNLVSGL

>starPep_03790

ASAAGAVREDDDETLLNPVLNSLDNLVSGL

>starPep_03795

ASIVKTTIKASKKLCRGFTLTCGCHFTGKK

>starPep_03803

AUIVKTTIKASKKLCRGFTLTCGCHFTGKK

>starPep_03816

CGESCVWIPCISAALGCSCKNKVCYRNGIP

>starPep_03855

DSAAGAVRAGDDETLLKPVLNSLDNLVSGL

>starPep_03976

GAGSQEERMQGQMEGQDFSHEERFLSMVRE

>starPep_03984

GFFTLIKAANKLINKTVNKEAGKGGLEIMA

>starPep_03999

GFMGDTLKGIARNAALALMNAAQCKLSGKC

>starPep_04039

GIPCAESCVWIPCTVTAIVGCSCSDKVCYN

>starPep_04040

GIPCGXSCVWIPCISSAIGCSCKSKVCYRN

>starPep_04109

GSVIKCGESCLLGKCYTPGCTCSRPICKKD

>starPep_04112

GTPCGSSCVYIPCISGVIGCSCTDKVCYLN

>starPep_04132

GWWRRTVDKVRNAGRKVAGFASKACGALGH

>starPep_04242

KFEPPLPPKKAHKKFWEDDGIYYPPNHNFP

>starPep_04614

QKLCERPSGTWSGVCGNNNACKNQCINLEK

>starPep_04642

RCICTTRTCRFPYRRLGTCIFQNRVYTFCC

>starPep_04740

SAPAPEVSGDAVFSAIQNGXLKNLGNAFFW

>starPep_04749

SGECNMYGRCPPGYCCSKFGYCGGVRAYCG

>starPep_04773

STPACAIGVVGITVAVTGISTACTSRCINK

>starPep_04864

WLSKTAKKLENSAKKRISEGIAIAIKGGSR

>starPep_04865

WLSKTYKKLENSAKKRISEGIAIAIQGGPR

>starPep_04866

WLSKTYKKLENSAKKRISEGVAIAILGGLR

>starPep_04918

ACYCRIPACIAGERRYGTCIYQGRLWAXCC

>starPep_04921

ADDRNPLEECFRETDYEEFLEIAKNGLSTT

>starPep_04922

ADDRNPLEECFRETDYEEFLEIARNGLKKT

>starPep_04941

AFFARLLASVRAAVKAFAKKPRLIGLSTLL

>starPep_05057

ASVVKTTIKASKKLCKGATLTCGCNITGKK

>starPep_05094

CAESCVWIPCTVTALLGCSCSNKVCYNGIP

>starPep_05095

CAESCVWIPCTVTALLGCSCSNNVCYNGIP

>starPep_05101

CESCVWIPCISSVVGCSCKSKVCYKNGTLP

>starPep_05105

CGESCVWIPCISSAIGCSCKNKVCYRNGIP

>starPep_05106

CGESCVWIPCISSAIGCSCKSKVCYRNGIP

>starPep_05107

CGESCVWIPCISSAVGCSCKNKVCYKNGTP

>starPep_05108

CGESCVWIPCLTSAIGCSCKSKVCYRNGIP

>starPep_05109

CGESCVWIPCLTSAVGCSCKSKVCYRNGIP

>starPep_05110

CGESCVWIPCTITALAGCKCKSKVCYNSIP

>starPep_05111

CGESCVYIPCLTSAIGCSCKSKVCYRNGIP

>starPep_05112

CGESCVYIPCLTSAVGCSCKSKVCYRNGIP

>starPep_05113

CGETCFTGTCYTNGCTCDPWPVCTRNGLPV

>starPep_05325

FKAFKAFKAFKAFKAFKAFKAFKAFKAFKA

>starPep_05431

FVNQHLCGSHLVEALYLVCGERGFFYTPKA

>starPep_05432

FVNQHLCGSHLVEALYLVCGERGFFYTPKT

>starPep_05611

GILMDTFKGAAKNVAGFLLDKLKCKISGGC

>starPep_05662

GLAGAISSVLDKLKQSQLIKNYAKKLGYPR

>starPep_05737

GLPCGESCVFIPCITTVVGCSCKNKVCYNN

>starPep_05815

GSVIKCGESCLLGKCYTPGCTCSRPICKKN

>starPep_05853

GWLRRIGRRIERVGQHKLKKALRALARHWK

>starPep_05858

GXFKKAXRKVKNAGRRVLKGVGIHYGVGLI

>starPep_05867

HAEGTFTSDVSSYLEGQAAKEFIAWLVKGR

>starPep_06132

IWEGIKNAGKGFLVSILDKVRCKVAGGCNP

>starPep_06151

KCPWWNLSCHLGNDGKICTYSHECTAGCNA

>starPep_06153

KCSWWNASCHLGNNGKICTVSHECAAGCNL

>starPep_06174

KFFARLLASVRAAVKKFRKKPRLIGLSTLL

>starPep_06185

KFKKFKKFKKFKKFKKFKKFKKFKKFKKFK

>starPep_06272

KLFKKIGIGAVLKVLKVLTTGLPALKLTLK

>starPep_06279

KLKKALRALARHWKGWLRRIGRRIERVGQH

>starPep_06579

LKKLKGRVSRSFLFFVKLRPAKRTLKKRIL

>starPep_06681

LRVRLASHLRKLRKRLLRDADDLQKRLAVY

>starPep_07463

RIAGYGLRGLAVIPRRICIRGLNLIFEIIR

>starPep_07710

SLGSFMKGVGKGLATVGKIVADQFGKLLEA

>starPep_07746

SVLSTITDMAKAAGRAALNAITGLVNQGEQ

>starPep_07774

TDHQMAQSACIGVSQDNAYASAIPRDCHGG

>starPep_07918

VTLASHLPSDFTPAVHASLDKFLANVSTVL

>starPep_07948

WEAKLAKALAKALAKHLAKALAKALKACEA

>starPep_08197

ACYCRAPACIAGERRYGTCIYQGRLWAFCC

>starPep_08198

ACYCRIPACIAGEAAYGTCIYQGALWAFCC

>starPep_08199

ACYCRIPACIAGERRAGTCAYQGRAWAACC

>starPep_08200

ACYCRIPACIAGERRAGTCIYQGRLWAACC

>starPep_08201

ACYCRIPACIAGERRAGTCIYQGRLWAFCC

>starPep_08202

ACYCRIPACIAGERRYATCIYQGRLWAFCC

>starPep_08203

ACYCRIPACIAGERRYGTCAYQGRAWAFCC

>starPep_08204

ACYCRIPACIAGERRYGTCAYQGRLWAFCC

>starPep_08205

ACYCRIPACIAGERRYGTCIAQGRLWAFCC

>starPep_08206

ACYCRIPACIAGERRYGTCIYAGRLWAFCC

>starPep_08207

ACYCRIPACIAGERRYGTCIYQGALWAFCC

>starPep_08208

ACYCRIPACIAGERRYGTCIYQGRLAAFCC

>starPep_08209

ACYCRIPACIAGERRYGTCIYQGRLWAACC

>starPep_08306

AINNALNKVCSTGRRQRSICKQLLKKLRQQ

>starPep_08360

ALAGTIIAGASLTFQVLDKVLEELGKVSRK

>starPep_08375

ALLHHGLNCAKGVLAALLHHGLNCAKGVLA

>starPep_08446

APWLVPSQITTCCGYNPGTMCPSCMCTNTC

>starPep_08450

AQEEAEAEERRLQEQEELENYIEHVLLRRP

>starPep_08478

ASKQASKQASKQASKQASKQASRSLKNHLL

>starPep_08560

AVSPTTLRTEVVKTFRRDKPFPHRMDCVTT

>starPep_08589

CCFLNITNSHVSILQERPPLENRVLTGWGL

>starPep_08611

CEEQAQQIRLQAEAFQARLKSWFEPLVEDM

>starPep_08653

CGESCVFIPCVTALLGCSCKSKVCYKNSIP

>starPep_08655

CGESCVYIPCISGVIGCSCTDKVCYLNGTP

>starPep_08662

CGETCVGGTCNTPGCTCSWPVCTRNGLNPV

>starPep_08855

CRFPNITNSHVPILQERPPLENRVLTGWGL

>starPep_08873

CSCKNKVCYRNGIPCGESCVWIPCISAALG

>starPep_08925

CTLISWIKNKRKQRPRVSRRRRRRGGRRRR

>starPep_08998

DCYCRIPACIAGEAAYGTCIYQGALWAFCC

>starPep_08999

DCYCRIPACIAGEKKYGTCIYQGKLWAFCC

>starPep_09001

DDCGKLFSGCDTNADCCEGYVCRLWCKLDW

>starPep_09023

DEDTAGLPGRQLPPCTSLLVGLMPCAAARS

>starPep_09114

DPTARLQLEARLQHLVAEILEREQSLALHA

>starPep_09161

DVTFSLLGANTKSYAAFITNFRKDVASEKK

>starPep_09200

EDVSAGEDCGPLPEGGPEPRSDGAKPGPRE

>starPep_09217

EERKKLGEEIKKEAEEAKKQIEETKKNDEE

>starPep_09239

ELAGTIIDGASLTFEVLDKVLGELGKVSRK

>starPep_09250

ELLFDGTNPSTEEMGDDFRSGLCPFDTSPV

>starPep_09315

EWDREINNYTSLIHSLIEESQNQQEKNEQE

>starPep_09587

FNVALDQVFESIENSQALVDQSNRILSSAE

>starPep_09680

GCCGPYPNAACHPCGCKVGRPPYCDRPSGG

>starPep_09806

GIGCGESCVWIPCVSAAIGCSCSNKICYRN

>starPep_09837

GINASVVNIQKEIDRLNEVAKNLNESLIDL

>starPep_09842

GIPCGESCVFIPCISSVVGCSCKSKVCYLD

>starPep_09843

GIPCGESCVFIPCISTVIGCSCKNKVCYRN

>starPep_09844

GIPCGESCVFIPCITGIAGCSCKSKVCYRN

>starPep_09845

GIPCGESCVFIPCLTSAIGCSCKSKVCYRN

>starPep_09846

GIPCGESCVWIPCISSAIGCSCXSXVCYRN

>starPep_09880

GLCPFDTSPVVKGKYNTTLLNGSAFYLVCP

>starPep_09903

GLKDMIKNLAKEAAVKLAGAVINKFSPQPQ

>starPep_09937

GLPCGESCVFIPCITTVVGCSCKNKVCYND

>starPep_09994

GQVWEATATVNAIRGSVTPAVSQFNARTAD

>starPep_10082

GTSCGETCVLLPCLSSVLGCTCQNKRCYKD

>starPep_10093

GVIPCGESCVFIPCINKKKCSCKNKVCYRD

>starPep_10123

GWTLNSAGYLLGPHAVGNHRSFSDKNGLTS

>starPep_10152

HALQDTEENPRSFPASQTEAHEDPDEMNED

>starPep_10155

HAPQDTEENARSFPASQTEPLEDPDQINED

>starPep_10547

KFFKKLKNSVKKRAKKFFKKPRVIGVSIPF

>starPep_10866

KMVQGSGCFGRKMDRISSSSGLGCKVLRRH

>starPep_10871

KNKKQTDILEKVKEILDKKKKTKSVGQKLY

>starPep_11068

LAEGPPVKECAVTCRYDKDADINVVTQARN

>starPep_11221

LLGDFFRKSKEKIGKEFKRIVQRIKDFLRN

>starPep_11401

MAGRSGDSDEELLKTVRLIKFLYQSNPPPS

>starPep_11711

MGAVAKFLGKAALGGAAGGATYAGLKKIFG

>starPep_11732

MIRIRSPTKKKLNRNSISDWKSNTSGRFFY

>starPep_11877

MPRVRSLFQEQEEPEPGMEEAGEMEQKQLQ

>starPep_11878

MPRVRSLFQRQKRTEPGLEEVGEIEQKQLQ

>starPep_11900

MRFGSLALVAYDSAIKHSWPRPSSVRRLRM

>starPep_12060

NNYTSLIHSLIEESQNQQEKNEQELLELDK

>starPep_12065

NPMYNAVSNADLMDFKNLLDHLEEKMPLED

>starPep_12067

NPPDHSAPLGATRPSAPPLPHVVDLPQLGP

>starPep_12071

NPVLVKDATGSTQFGPVQALGAQYSMWKLK

>starPep_12115

PAIAQRATATLGTVGSNTSGTTEIEACILL

>starPep_12117

PAICQRATATLGTVGSNTSGTTAIEACILL

>starPep_12118

PAICQRATATLGTVGSNTSGTTEIAACILL

>starPep_12119

PAICQRATATLGTVGSNTSGTTEIEAAILL

>starPep_12120

PAICQRATATLGTVGSNTSGTTEIEACILL

>starPep_12122

PAIYIGATVGPSVWAYLVALVGAAAVTAAN

>starPep_12141

PDEDAINDALNKVCSTGRRQRSICKQLLKK

>starPep_12142

PDEDAINNALNKVCSTGRRQRSICKQLLKK

>starPep_12173

PKMVQGSGCFGRKMDRISSSSGLGCKVLRR

>starPep_12269

PSQPTYPGDDAPLEDLMAFAIDLSFYLGVV

>starPep_12306

QDYCAQEGQQEVQRKDLSDLERYLRQSRQR

>starPep_12812

RSLQDTEEKSRSFSASQADPLSDPDQMNED

>starPep_12813

RSLQNTEEKSSSFPAPQTDPLGDPDQINED

>starPep_12913

SCSGRDSRCPPVCCMGLMCSRGKCVSIYGE

>starPep_12920

SCVWIPCISAALGCSCKNKVCYRNGIPCGE

>starPep_12944

SFLNFFKGAAKNLLAAGLDKLKCKISGTQC

>starPep_13026

SPKMVQGSGCFGRKMDRISSSSGLGCKVLR

>starPep_13041

SQGVVESMNKELKKIIGQVRDQAEHLKTAY

>starPep_13042

SQGVVESMNKELPKIIGQVRDQAEHLKTAY

>starPep_13066

SSPETLISDLLMKESTENAPRTRLEDPSMW

>starPep_13067

SSPETLISDLLMRESTENAPRTRLEDPSMW

>starPep_13068

SSPETLISDLLMRESTENVPRTRLEDPAMW

>starPep_13159

TCGETCFGGTCNTPGCTCDPWPICTDRGLP

>starPep_13234

TLISWIKNKRKQCRPRVSRRRRRRGGRRRR

>starPep_13235

TLISWIKNKRKQRPRVSRRRRRRGGRRRRC

>starPep_13237

TLKNRYYEPRDSYFQQYMLKGEYQYWFDLD

>starPep_13246

TLQPPSALRRRHYHHALPPSRHYPGREAQA

>starPep_13306

TSVPYPRPFPRPPIGPRPLPFPGGGRPFQS

>starPep_13316

TTVKVHASDERLGPMPCRPKEIVSSAGPVM

>starPep_13390

VEDTLYGDHECGSLLQDAALYLVDGMTNTI

>starPep_13412

VGECVRGRCPSGMCCSQAGYCGKGPKYCGR

>starPep_13414

VGECVRGRCPSGMCCSQWGYCGKGPKYCGR

>starPep_13417

VGHNADLQIKLSIRRLLAAGVLKQTKGVGA

>starPep_13444

VKQCRWCGFDFDGPDGLPHYPIGKCILANE

>starPep_13497

VPTHLATDVELKEIQGMMDASEGTNYTCCK

>starPep_13542

VSRRYLASLHKKALPTSVTFELLFDGTNPS

>starPep_13574

VVNIQKEIDRLNEVAKNLNESLIDLQELGK

>starPep_13609

WCNWHNIDPWIQLMNRTQADLAEGPPVKEC

>starPep_13762

WYKHVASPRYHTVGRAAGLLMGLRRSPYLW

>starPep_13764

WYKHVASPRYHTVGRASGLLMGLRRSPYLW

>starPep_13873

YCQKWMWTCDSERKCCEGMVCRLWCKKKLW

>starPep_13877

YDPEAASAPGSGNPCHEASAAQKENAGEDP

>starPep_13900

YGGFMKSWDERSQKPLLTLFKNVIIKDGQQ

>starPep_13945

YLRGVNRSLHGIWPGKICKGVPTHLATDVE

>starPep_14183

AANQHLCGSHLVEALYLVCGERGFFYSPKA

>starPep_14184

AANQHLCGSHLVEALYLVCGERGFFYSPKT

>starPep_14237

AAXNXFALADYWGNNGAWAXLXHEAMAWAK

>starPep_14318

ACVELGEICATGFFLDEECCTGSCHVFCVL

>starPep_14322

ACVPVYKECWYPQKPCCEDRVCQCSFGMTN

>starPep_14325

ACYCRIPACLAGERRYGTCIYQGRLWAFCC

>starPep_14354

ADPRVVVDLRFFGKSDIVEENRVTFGPNPK

>starPep_14362

ADVFDRGGPYLQRGVADLVPTATLLDTYSP

>starPep_14406

AELTSCFPVDHECDGGASNCNCCGDDVYCA

>starPep_14638

AIAEYAARIEALLRAAQEQQEKLEAALREL

>starPep_14640

AIAEYAARIEALLRALQEQQEKNEAALREL

>starPep_14753

AKKPFVQRVKNAASKAYNKLKGLAMQSQYG

>starPep_14880

ALGTLLKGVGSAVATVGKMVADQFGKLLQA

>starPep_14905

ALGVATSAQITAAVALVEAKQARSDIEKLK

>starPep_14963

ALQFIQNTASGALYYNTKTHKYQYQQTSGA

>starPep_15135

APCGESCVYIPCLLTAPIGCSCSNIVCYRN

>starPep_15210

APNVKDSKASGSCCDNPSCAVNNRHCGRRR

>starPep_15212

APNVKDSKASGSCCDNPSCAVNNSHCGRRR

>starPep_15299

APWLVPSQITTCCGYNPGTMCPSCMCTNSC

>starPep_15303

APWLVPSTITTCCGYNPGTMCPPCRCDNTC

>starPep_15323

AQEEADAEERRLQEQEELENYIEHVLLHRP

>starPep_15349

AQQHLLQLTVWQIKQLQARILAVERYLKDQ

>starPep_15389

ARFLHPFQYYTLYRYLTRFLHRYPIYYIRY

>starPep_15562

ASIIKTTIKVCKAVSKTLTCICTGCCSNSK

>starPep_15563

ASIIKTTIKVCKAVSKTLTCICTGSCSNCK

>starPep_15564

ASIIKTTIKVSKAVCKTLTCICTGCCSNSK

>starPep_15610

ASSQHLCGSHLVDALYMVCGEKGFFYQPKT

>starPep_15755

AVFTVVNQCPFTVWAASVPVGGGRQLNRGE

>starPep_15762

AVHKECKTDVDCRQIWFVTKCINHECQPIL

>starPep_15797

AVNQHLCGSHLVEALYLVCGERGFFYSPKA

>starPep_15912

AXIIKXXIKVAKAVAKXLXAIAXGAAXNAK

>starPep_16074

CCCCCTTCCFSIATGSGNSQGGSGSYTPGK

>starPep_16359

CFFDRIKALTKNVTLELLNTITCKLPVTPP

>starPep_16392

CGESCVFIPCISSVIGCSCSSKVCYRNGIP

>starPep_16393

CGESCVFIPCLTSAIDCSCKSKVCYRNGIP

>starPep_16396

CGESCVWIPCISAVGCSCKSKVCYKNGTLP

>starPep_16397

CGESCVWIPCLTSVFNCKCENKVCYHDKIP

>starPep_16405

CGETCFGGTCNTPGCICDPWPVCTRNGLPV

>starPep_16406

CGETCFGGTCNTPGCSCSSWPICTRNGLPV

>starPep_16416

CGETCVGGTCNTPGCTCFKPWRCTRNGLPV

>starPep_16424

CGETCVGGTCNTPGCTCSWPVCLYRNGKPV

>starPep_16426

CGETCVGGTCNTPGCTCSWPVCRDHSYQEE

>starPep_16433

CGETCVGGTCNTPGCTCSWPVCYRSPFSRV

>starPep_16440

CGETCVVDTRCYTKKCSCAWPVCMRNSLAG

>starPep_16504

CHHEGLPCTSGDGCCGMECCGGVCSSHCGN

>starPep_16510

CIDGGEICDIFFFKLLQWVVHYSCLRMKLP

>starPep_16547

CIVGTPCHVCRSQSKSCNGWLEKQRYCGYC

>starPep_16548

CIVGTPCHVCRSQSKSCNGWLGKQGYCGYC

>starPep_16549

CIVGTPCHVCRSQSKSCNGWLGKQRYCGYC

>starPep_16701

CLAPQRWCSMHDDSLHDDNCCKTCIILWCS

>starPep_16861

CQPNGYYCDFEFTPKCCLKCDYNRKYCQPY

>starPep_16976

CSCRYSSCRFGERLLSGACRLNGRIYRLCC

>starPep_16978

CSCSSLMDKECVYFCHLDIIWVNTPEHIVP

>starPep_16997

CSNFGSDCIPATHDCCSGECFGFEDMGLCT

>starPep_17037

CTDSCEFQGRFVSGYLRLLRNKQLLLCHKP

>starPep_17087

CTQDFDPCMPVCHECCTRSHFVVCRRPICL

>starPep_17098

CTSPKQCLPPCKAQFGQSAGAKCMNGKCKC

>starPep_17167

CVSPGGVCQHKDECCSDRCEQSAIVSICKQ

>starPep_17277

DAEVVSTESDVIVTCEPCMNPACGPNYGKC

>starPep_17337

DCCPVAGMPLWMQPLLWMTSFVIGTSSSNE

>starPep_17345

DCGEQGEGCATRPCCEELSCVGSRPGGLCQ

>starPep_17370

DCLPDWFHYEGHCYRVFDEPKKWADAEKFC

>starPep_17372

DCLPGWSSHEGHCYKVFNQEMYWADAEKFC

>starPep_17378

DCPSDWSPYEGHCYKHFIKWMNNEDAERFC

>starPep_17397

DCSGSGYGCKNTPCCDGLTCRGPHQGPICL

>starPep_17429

DDCGGLFSGCDSNADCCEGYVCRLWCKYKL

>starPep_17439

DDDCEPPGNFCGMIKIGPPCCSGWCFFACA

>starPep_17464

DDECEPPGDFCGFFKIGPPCCSGWCFLWCA

>starPep_17466

DDECEPPGDFCGFFKIGPPCCSGWCFLWCS

>starPep_17467

DDECEPPGDFCGFFKIGPPCCSGWCFLWCV

>starPep_17470

DDECEPPGDFCGFLKLGPPCCSGWCFLWCA

>starPep_17471

DDECEPPGDFCGLFKIGPPCCSGWCFLWCA

>starPep_17473

DDECEPPGVFCGTFKIGPPCCSGWCFLWCA

>starPep_17645

DFFRKSKEKIGKEFKRIVQRIKDFLRNLVP

>starPep_17858

DKPKRKKKGGKNGKNRRNRKKKNPCNAEFQ

>starPep_18030

DPYAEAASGPNPGSKSHESAQAENCGADPE

>starPep_18267

DWCGDAGDACGTLKLRCCSGLCNQYSGTCT

>starPep_18321

DYDCEPPGNFCGMIKIGPPCCSGWCFFACA

>starPep_18481

ECREKGQGCTNTALCCPGLECEGQGSKELC

>starPep_18488

ECREKGQGCTNTALCCPGLECEGRGSKELC

>starPep_18525

ECTPPGGACNIHPHCCEEFCDMANNRCLEM

>starPep_18541

ECWSQAADCSDGHCCAGRSFSKNCRPYGGD

>starPep_18575

EDMNQKLFDLRGKFKRPPLRRVRMSADAML

>starPep_18616

EEEEEEEEEEKKRLKKIFKKPMVIGVTIPF

>starPep_18755

EILNIIDSISDVAKQICCQITVDCCVLDEE

>starPep_18767

EIVNIIDSISDVAKQICCEITVQCCVLDEE

>starPep_18823

EKSLVPSVITTCCGYDPGTMCPPCRCTNSC

>starPep_18969

EMIAAAIRAEKSRGGSSRQSIQKYIKSHYK

>starPep_19249

EWEREIDNYTSLIYSLIEESQNQQEKNEQE

>starPep_19282

EYXSPSQGXQSQXSGGGGXGGGGGGGGAQN

>starPep_19310

FANQHLCGSHLVEALYLVCGERGFFYTPKA

>starPep_19653

FINKAGKLQSQLRTTVVAAAAFLDAFQKVA

>starPep_20211

FPNQHLCGSHLVEALYLVCGEKGFYYIPRM

>starPep_20329

FSCDHSACAVRCLAQRRKGGKCKNGDCVCR

>starPep_20414

FVBQHLCGSHLVEALYLVCGERGFFYTPKS

>starPep_20429

FVKQHLCGPHLVEALYLVCGERGFFYTPKS

>starPep_20430

FVKQHLCGSHLVEALYLVCGERGFFYTPMS

>starPep_20435

FVNKHLCGSHLVDALYLVCGDRGFFYTPMA

>starPep_20438

FVNQHLCGPHLVEALYLVCGERGFFYAPKT

>starPep_20440

FVNQHLCGSHLVEALYLVCGERGFFYTPKF

>starPep_20441

FVNQHLCGSHLVEALYLVCGERGFFYTPKS

>starPep_20442

FVNQHLCGSHLVEALYLVCGNDGFFYRPKA

>starPep_20470

FVSRHLCGSNLVETLYSVCQDDGFFYIPKD

>starPep_20496

FWNWLSAWKDLELYPGSLELDKWASLWNWF

>starPep_20592

FYDPLVFPSDEFDASISQVNEKINQSLAFI

>starPep_20628

GAFGDFLKGAAKKAGLKILSIAQCKLFGTC

>starPep_20629

GAFGDFLKGAAKKAGLKILSIAQCKLSGTC

>starPep_20630

GAFTDLLKGVAKQAGIKILGIAQCKLAKTC

>starPep_20633

GAGCIETCYTFPCISEMINCSCKNSRCQKN

>starPep_20684

GAPVCGETCFGGTCNTPGCTCDPWPVCTND

>starPep_20760

GCCGKYPNAACHPCGCTVGRPPYCDRPSGG

>starPep_20761

GCCGKYXNAACHXCGCTVGRXXYCDRXSGG

>starPep_20764

GCCGPYXNAACHXCGCKVGRXXYCDRXSGG

>starPep_21015

GCLDPGYFCGTPFLGAYCCGGICLIVCIET

>starPep_21022

GCLPDEYFCGFSMIGALLCCSGWCLGICMT

>starPep_21033

GCPRILMRCKQDSDCLAGCVCGPNGFCGSP

>starPep_21126

GDLFKCGETCFGGTCYTPGCSCDYPICKKN

>starPep_21127

GDLFKCGETCFGGTCYTPGCSCDYPICKNN

>starPep_21132

GDPFKCGESCFAGKCYTPGCTCEYPICMNN

>starPep_21135

GDPLKCGESCFAGKCYTPGCTCDRPICKKN

>starPep_21136

GDPLKCGESCFAGKCYTPGCTCEYPICMNN

>starPep_21137

GDPLKCGESCFAGKCYTPGCTCSRPICKKN

>starPep_21166

GECDGKKDCITNDDCTGCLCSDFGSYRKCA

>starPep_21188

GEFLKCGESCVQGECYTPGCSCDYPICKNN

>starPep_21206

GEPVCGDSCVFFGCDDEGCTCGPWSLCYRN

>starPep_21211

GETCFGGTCNTPGCTCDPWPVCTRNGLPVC

>starPep_21238

GFASFLGKALKALKAALKIGANALGGAPQQ

>starPep_21772

GFFDRIKALTKNVTLELLNTITCKLGVTGG

>starPep_21773

GFFDRIKALTKNVTLELLNTITGKLGVTGG

>starPep_21774

GFFDRIKALTKNVTLELLNTITGKLPVTPP

>starPep_21781

GFFPLIKGPAKLIAKTVAKKPAKTGLEFML

>starPep_21833

GFICGESCVYIPCITALLGCSCSNQICSKN

>starPep_21907

GFPCGESCVYVPCLTAAIGCSCSNKVCYKN

>starPep_21925

GFSSIFRGVAKFASKGLGKDLAKLGVDLVA

>starPep_22010

GGGGSGGGGSGGGGSGGGGSGGGGSGGGGS

>starPep_22014

GGGIEEKIEEIEELLQLTVWGIKQLQARIL

>starPep_22095

GGSIPCGESCVFLPCFLPGCSCKSSVCYLN

>starPep_22115

GGVCGETCVGGTCNTPGCKCSWPVCGSGSD

>starPep_22116

GGVCGETCVGGTCNTPGCTCSWPVCGSGSD

>starPep_22212

GIFLDKLKNFGKDVAGILLKKASCALSGQC

>starPep_22357

GIKHILFMAKTKLPRATCTAEIKENCDRKK

>starPep_22376

GILDTLKQFAKGVGKDLVKGAAQGVLSTVS

>starPep_22388

GILLDKLKNFAKGVAQSLLNKASCALSGQC

>starPep_22431

GIPCAESCVWIPCTVTAIVGCSCSWGVCYN

>starPep_22432

GIPCAESCVWIPCTVTALLGCSCKDKVCYN

>starPep_22433

GIPCAESCVWIPCTVTKMLGCSCKDKVCYN

>starPep_22435

GIPCAESCVYIPCLTSAIGCSCKSKVCYRN

>starPep_22440

GIPCGDSCHYIPCVTSTIGCSCTNGSCMRN

>starPep_22441

GIPCGEGCVYLPCFTAPLGCSCSSKVCYRN

>starPep_22442

GIPCGESCAWIPCISAVQGCSCRNKICYRN

>starPep_22444

GIPCGESCHYIPCVTSAIGCSCRNRSCMRN

>starPep_22446

GIPCGESCMWIPCISAAIGCSCTNHVCYKN

>starPep_22447

GIPCGESCVFIPCFTSVFGCSCKDKVCYRN

>starPep_22448

GIPCGESCVFIPCISALLGCSCSNKVCYNN

>starPep_22449

GIPCGESCVFIPCISAVVGCSCSNKVCYNN

>starPep_22450

GIPCGESCVFIPCISSVVGCSCKSKVCYNN

>starPep_22451

GIPCGESCVFIPCITAAIGCSCKTKVCYRN

>starPep_22452

GIPCGESCVFIPCITGAIGCSCKSKVCYRD

>starPep_22454

GIPCGESCVFIPCLTAAIGCSCRSKVCYRN

>starPep_22455

GIPCGESCVFIPCLTTVVGCSCKNKVCYNN

>starPep_22461

GIPCGESCVFIPCVTTVIGCSCKDKVCYNN

>starPep_22463

GIPCGESCVLIPCISSVIGCSCKSKVCYRN

>starPep_22464

GIPCGESCVWIPCISGAIGCSCKSKVCYKN

>starPep_22465

GIPCGESCVWIPCISGAIGCSCKSKVCYRN

>starPep_22466

GIPCGESCVWIPCISGVQGCSCSNKICYRN

>starPep_22468

GIPCGESCVWIPCISSAIGCSCKNKVCFKN

>starPep_22470

GIPCGESCVWIPCISSAIGCSCKNKVCYRK

>starPep_22471

GIPCGESCVWIPCISSAIGCSCKSKVCYXN

>starPep_22472

GIPCGESCVWIPCISSAIGCSCXSXVCYXN

>starPep_22473

GIPCGESCVWIPCISSALGCSCKNKVCYRN

>starPep_22474

GIPCGESCVWIPCISSALGCSCKSKVCYRN

>starPep_22475

GIPCGESCVWIPCITSAVGCSCKSKVCYRN

>starPep_22476

GIPCGESCVWIPCLTAAIGCSCSSKVCYRN

>starPep_22477

GIPCGESCVWIPCLTATIGCSCKSKVCYRN

>starPep_22478

GIPCGESCVWMYCITATMGCSCRNKVCYKN

>starPep_22481

GIPCGESCVYLPCFTAPLGCSCSSKVCYRN

>starPep_22484

GIPCGETCVFMPCISGPMGCSCKHMVCYRN

>starPep_22485

GIPCGGSCVWIPCISGVQGCSCSNKICYRN

>starPep_22635

GKLGKDAVEDLESVGKGAVHDVKDVLDSVL

>starPep_22829

GLFKTLIKGAGKMLGHVAKEFLGSEGEPES

>starPep_22830

GLFKTLIKGAGKMLGHVAKEFLGSQGEPES

>starPep_22831

GLFKTLIKGAGKMLGHVAKEFLGSQGQPES

>starPep_22940

GLKDMIKNLAKEAAVKLAGAVINRFSPQPQ

>starPep_23164

GLPCGESCIWIECISGAIGCSCRNKVCYRN

>starPep_23168

GLPICGETCFGGTCNTPGCICDPWPVCTRD

>starPep_23169

GLPICGETCFGGTCNTPNCVCDPWPICTNN

>starPep_23181

GLPTCGETCFGGTCNTPGCTCDPFPVCTHD

>starPep_23195

GLPVCGETCFGGTCNTPGCACDPWPVCTRN

>starPep_23196

GLPVCGETCFGGTCNTPGCSCDPWPVCTRN

>starPep_23197

GLPVCGETCFGGTCNTPGCSCXTWPVCSRN

>starPep_23201

GLPVCGETCRRDSDCNTPGCTCSWPVCTRN

>starPep_23220

GLPVCGETCVGGTCNTPGCKCRGNGYCTRN

>starPep_23237

GLPVCGETCVGGTCNTPGCTCRGNGYCTRN

>starPep_23248

GLPVCGETCVGGTCNTPGCTCSRVVHCTRN

>starPep_23258

GLPVCGETCVGGTCNTPGCTCSWWPVCTRN

>starPep_23272

GLPVCGETCVGPTCNTPGCTCYRSPFCTRN

>starPep_23435

GMSGYIQGIPDFLKGYLHGISAANKHKKGR

>starPep_23498

GNPIVCGETCFFQKCYTPGCSCDAVICTNN

>starPep_23774

GRKKRRQRRRPPQTYADFIASGRTGRRNAI

>starPep_23806

GRPMGHQAIETGLNIFRGLFKGKKKNKKTK

>starPep_23883

GSAFGCGETCVKGKCNTPGCVCSWPVCKKN

>starPep_23884

GSAIACGESCFKFKCYTPGCSCSYPICKKD

>starPep_23885

GSAILCGESCTLGECYTPGCTCSWPICTKN

>starPep_23886

GSAIRCGERCLLGRCHRPGCTCIRRICRRN

>starPep_23963

GSPTCGETCFGGTCYTPGCVCDPWPICTKN

>starPep_23964

GSPTCGETCFGGTCYTPNCVCDPWPICTKN

>starPep_23998

GSSPLCGETCAGGTCNTPGCSCSWPVCVRD

>starPep_24013

GSVFNCGETCVFGTCFTSGCSCVYRVCSKD

>starPep_24047

GTDAKVKLIKQELDKYKNAVTELQLLMQST

>starPep_24065

GTPCGESCIYVPCISAVFGCWCQSKVCYKD

>starPep_24099

GTYLYPFSYYRLWRYFTRFLHKQPYYYVHI

>starPep_24183

GVPCAESCVYIPCISTVLGCSCSNQVCYRN

>starPep_24185

GVPCGESCVFIPCLTAVVGCSCSNKVCYLN

>starPep_24186

GVPCGESCVWIPCLTSAIGCSCKSSVCYRN

>starPep_24188

GVPCGESCVWMYCISAAMGCSCRNKVCYRN

>starPep_24190

GVPCGESCVYIPCITSVIGCSCSSKVCYIN

>starPep_24276

GWFGKAFRSVSNFYKKHKTYIHAGLSAATL

>starPep_24356

GWVAVVGACGTVCLASGGVGTEFAAASYFL

>starPep_24370

GXFKKTFHKVSHAVKSGIHAGQRGCSALGF

>starPep_24396

GXXRRTVDKVRNAGRKVAGFASKACGALGH

>starPep_24457

HADGRYTSDISSYLEGQAAKEFIAWLVNGR

>starPep_24491

HAEGTYTSDITSYLEGQAAKEFIAWLVNGR

>starPep_24561

HGKKVLDSFSNGMKHLDDLKGTFAALSELH

>starPep_24725

HPLQDTEEKPRSFSTSQTDLLDDPDQMNED

>starPep_24768

HRRSVAHQEEASLHVKTDELPSPDTVREQL

>starPep_24769

HRRSVAHQQQASLHVKTNQLPSPNTVRQQL

>starPep_24810

HSLQDTEEKSRSFPASQTDPLEDPDQINED

>starPep_24812

HSLQNTEEKSSSFPAPQTDPLGDPDQISED

>starPep_24947

IATKKNGRKLCLDLQAALYKKKIIKKLLES

>starPep_25014

IEAQQHLLQLTVWGIKQLQARILAVERYLK

>starPep_25017

IEEDCGYVPCEFGCCRIIDGKEKCREIDCQ

>starPep_25018

IEEDCGYVXCEFGCCRIIDGKEKCREIDCQ

>starPep_25711

IPCATSDDCLKNMCRPPLTPRCIEHNCKCK

>starPep_25932

ISLNPPRSTIAMRAINNYRWRSKNQNTFLR

>starPep_26001

ITLNNSVALDPIDISIELNKAKSDLEESKE

>starPep_26173

KAFITLALGQEGCCPSGPCHFAACNPPCCT

>starPep_26251

KCAAAGEACVIPIIGNVFCCKGYCLFVCIS

>starPep_26252

KCALERGEVCVVPILGTLACCRAFCSGVCL

>starPep_26284

KCMEQGTYCSLILFSSSCCGDLCLFGFCIL

>starPep_26296

KCPWWNLXCHLGNDGKIXXYXHEXTAGXNA

>starPep_26364

KEEHGKRKKKGKGLGKKRDPCLRKYKDFCI

>starPep_26370

KEGYIVNYHTGCKYTCAKLGDNDYCLRECK

>starPep_26405

KEVKRGCVATCPKPKKNEIIQCCAKDKCNK

>starPep_26419

KFFKKLKNSVKKRAKKFFKKPKVIGVTFPF

>starPep_26420

KFFRKLAKSVKKAAKEFFKKPRVIGVSIPF

>starPep_26421

KFFRKLKKSVKKRAKKFFKKPRVIGVSIPF

>starPep_26570

KHVRDCPKGIWRSCRYKCIDNKCVFTYWPH

>starPep_26617

KIFKKIEKVGRNVRDGIIKAGPAVAVVEQA

>starPep_26668

KIPCGESCVWIPCFTSAFGCYCQSKVCYHS

>starPep_26669

KIPCGESCVWIPCISSILGCSCKDKVCYHN

>starPep_26885

KKLFKKILKYLAGPAKKLFKKILKYLAGPA

>starPep_26889

KKLFKKILKYLAGPAKKLFKKILKYLKDEL

>starPep_26890

KKLFKKILKYLAGPALYKLIKKFLKKKDEL

>starPep_27266

KLWKLFKKIGIGAVLKVLTTGLPALKLTLK

>starPep_27320

KNECLWTDMLSNFGYPGYQSKHYACIRQKG

>starPep_27443

KQATCSIPYEYSNGKFKRTLYYSNGVYANS

>starPep_27548

KRIPNKKPGKKTTTKPTKKPTIKTTKKDLK

>starPep_27569

KRKFHEKHHSHRGYCCYGRHSHHKEHFKRK

>starPep_27847

KVLCGRDGTCPRFMCGPGIIPKCVGRYCEC

>starPep_28241

KYYGNGVHCTKSGCSVNWGEAFSAGVHRLA

>starPep_28292

LANQHLCGSHLVEALYLVCGDRGFFYYPKI

>starPep_28369

LDKRCIPHFDPCDPIRHTCCFGLCLLIACI

>starPep_28421

LEANISQSLEQAQIQQEKNMYELQKLNSWD

>starPep_28435

LEDPYKSDSNSRYIEVVVVNDNSMFRKYNR

>starPep_28569

LGKLGKDAVEDLESVGKGAVHDVKDVLDSV

>starPep_28581

LGPQLNKGCATCSIGAACLVDGPIPDEIAG

>starPep_28651

LIFCFEDINCPFDKCFPQLPKCINSFCECV

>starPep_28701

LIVSETNPTQVVAALLRNLNTSNDELVVAK

>starPep_28726

LKCNKLIPLAYKTCPAGKDLCYKMYMVSDK

>starPep_28727

LKCNKLIPLAYKTCPAGKNLCYKMYMVATP

>starPep_28731

LKCNKLVPLFYKTCPAGKDLCYKMYMVATP

>starPep_28733

LKCNKLVPLFYKTCPAGKNLCYKMFMVSNK

>starPep_28744

LKDGYPTNSKGCKISGCLPGENKFCLNECQ

>starPep_29137

LNKRCIDGGEICDIFFPNCCSGWCIILVCA

>starPep_29138

LNKRCLDGGEICGILFPSCCSGWCIVLVCA

>starPep_29381

LRKRLRKFRNKIKEKLKKIGQKIQGFVPKL

>starPep_29527

LSWDLPEPRSRAGKIRVHPRGNLWATGHFM

>starPep_29626

LVNQHLCGSHLVEALYLVCGDRGFFYYPKV

>starPep_29627

LVNQHLCGSHLVEALYLVCGERGFFYTPKA

>starPep_30092

MAKWKLFKKIGIGAVLKVLTTGLPALKLTK

>starPep_30101

MAKWKLFKKIGIGFKKAAHVGKAALTKXXX

>starPep_30233

MARSVPLVSTIFVFFLLIVATEMGPSMVAA

>starPep_30427

MDKGEVADLTAWLEKVKEIQERYPLPEEPK

>starPep_30628

MFLKAVVLTVALVAITGTQAEVTSDQVANV

>starPep_31759

MGAAVKMLGKAFAGGVAGGATYGGLKKIFG

>starPep_31764

MGAIIKGAAKVLGKGAATGGVIYGLEKLFR

>starPep_31765

MGAIIKGGLKLVGGGAAGGFTYGGLKKIFG

>starPep_31768

MGALFKAALKAAGGGAAGGATYGGLKHFFG

>starPep_31769

MGALVKGGLKLIGGTAASWLGWEAGERVWK

>starPep_31774

MGAVVKGGLKIIGGTAASWLGWEAGTRIWK

>starPep_31789

MGFVLFSQLPSFLLVSTLLLFLVISHSCRA

>starPep_31877

MICYSHKTPQPSATIGCEEKTCYKKSVRKL

>starPep_31892

MIHLTKQNTMEALHFIKQFYDMFFILNFNV

>starPep_32078

MKFTATFLXXXLLFIFXVLMXVEDPLGECG

>starPep_32630

MLSLIFLHRLKSMRKRLDRKLRLWHRKNYP

>starPep_32641

MLTAEEKAAVTAFWGKVKVDEVGGEALGRL

>starPep_32861

MNYLVFFSLALLVMTGRTVTREKRKDMMDL

>starPep_32892

MPKARPVNHNKKKSKITIKSNFTLFYMFNP

>starPep_32935

MPTEERVRKRKESNRESARRSRYRKAAHLK

>starPep_33260

MSKELEKVLESSSMAKGDGWKVMAKGDGWE

>starPep_33571

MTVKIAQKKVLPVIGRAAALCGSCYPCSCM

>starPep_33610

MVILVFSLIFIFTDNYLVYQSKSIKEDVMI

>starPep_33622

MVKSKIGSWILVLFVAMWSDVGLCKKRPKP

>starPep_33770

NAAAKAFDLKGCCSHPACSGNYQEYCRESY

>starPep_33782

NAEEGTAVPYVPGYHKKNEIEFQKDIDRFV

>starPep_33789

NAGAPQHLCGSHLVDALYLVCGPTGFFYNP

>starPep_33990

NGVIPCGESCVFIPCISTLLGCSKNKVCYR

>starPep_34059

NIVDVPCRDDYYRDSSGNGVYDQLGGCGAA

>starPep_34328

NLLQFAFMIRQANKRRRPVIPYEEYGLYYM

>starPep_34360

NLYQFGGMIGCANKGTRSWLSYVNYGCYCG

>starPep_34363

NLYQFKNMIHCTVPSRPWWHFADYGCYCGR

>starPep_34367

NLYQFKNMVQCVGTQLCVAYVKYGCYCGPG

>starPep_34429

NNLLRAIEAQQHLLQLTVWQIKQLQARILA

>starPep_34481

NPGTPQHLCGSHLVDALYLVCGPTGFFYNP

>starPep_34491

NPLQDTEEKSRSFKASQSEPLDESRQLNEV

>starPep_34498

NPMYNVVSNADLVDFKNLLDHLEEKMPLED

>starPep_34510

NPVYGSVSNADLMDFKNLLDHLEDKMPLED

>starPep_34593

NSASLISSWVDNTNFCCCSHDCATICDDCF

>starPep_34798

PCISDDDCPEALSPQFPKCIHNVCVYFVEE

>starPep_35173

PSDEFDASISQVNEKINQSLAFIRKSDELL

>starPep_35388

QCTPVGGYCFDHHHCCSNHCIKSIGRCVAH

>starPep_35389

QCTPVGGYCSRHHHCCSNHCIKSIGRCVAH

>starPep_35390

QCTPVGGYCSRHYHCCSNHCIKSIGRCVAH

>starPep_35559

QHGKISSEQHTMFDPIEGCCQQSCTTCFPC

>starPep_35649

QKDLVVTATTTCCGYNPMTMCPPCMCTNTC

>starPep_35735

QKSLVPSVITTCCGYDPGTMCPPCRCTNSC

>starPep_35749

QLEARFEPKQRNFRKRELDFEKLFANMPDY

>starPep_35907

QPRFESCQQDSDCDFQFFCWNNECHRIILI

>starPep_35970

QRACPRILKKCRRDSDCPGECICKENGYCG

>starPep_36052

QSLEQAQIQQEKNMYELQKLNSWDVFTNWL

>starPep_36071

QSWLVPSTITTCCGYSPGTMCPPCMCTNTC

>starPep_36097

QTQCQSVRDCQQYCLTPDRCSYGTCYCKTT

>starPep_36100

QTQYTDAPSFSDIPNPIGSENSEKTTMPLW

>starPep_36118

QVKCKTVKDCPIRRNRKYYCLFGICKYDVM

>starPep_36137

QVPIRPSQPRPQPFKPRPQQVPPRTPHPRL

>starPep_36257

RCAHGTYYSNDSQQCLLNCCWWGGGDHCCR

>starPep_36290

RCDEEGTGCSSDSECCSGRCTPEGLFEFCE

>starPep_36503

RGFMDTAKNVAKNMAVTLLDNLKCKITKAC

>starPep_36528

RGLRRLGRKIAHGVKKYGPTVLRIIRIAGC

>starPep_36534

RGLWSKIKEAGKAALTAAGKAALGAVSDAV

>starPep_36700

RIKAERKRMRNRIAASKSRKRKLERIARGC

>starPep_36945

RKLPDAPGMHTWGGGGKCSTRGRKCCRRKK

>starPep_36984

RKSKEKIGKEFKRIVQRIKDFLRNLVPRTE

>starPep_38006

RRRFVVQQDTISPRLEVDERFLPNSVQEQI

>starPep_38007

RRRFVVQQNTISPRLQVNQRFLPNSVQQQI

>starPep_38088

RRRSVGEEDAIPSHIEVNKFFLRKPAKEHI

>starPep_38089

RRRSVGQQNAIPSHIQVNKFFLRKPAKQHI

>starPep_38138

RRVSRRFMRRSGSGSGSGQQMVQQSGQQFS

>starPep_38139

RRVSRRFMRRSRWARKGSGQMVQQSSQQFQ

>starPep_38454

RSLQDTEEKPRSVSASQTDMLDDPDQMNED

>starPep_38456

RSLQDTEEKSRSFSAPQTEPLNDLDQMNED

>starPep_38459

RSLQNTEEKSRSFPAPQTDPLDDPDQMTED

>starPep_38626

RVKASRRSASHPTYSEMIAAAIRAEKSRGG

>starPep_38835

RWRQQWSGPGTTKRFPETVLARCVKYTEIH

>starPep_38837

RWRQTWSGPGTTKRFPETVLARCVKYTEIH

>starPep_39214

SCAQSGLSCDTRPCCDDKPCVPNGRQSMCG

>starPep_39243

SCGHSGAGCYSRPCCPGLHCSGTHAGGMCV

>starPep_39244

SCGHSGAGCYTRPCCPGLHCSGGHAGGLCV

>starPep_39245

SCGHSGAGCYTRPCCPGLHCSGGQAGGLCV

>starPep_39247

SCGNLGESCSAHRCCPGLMCMGEASICIPY

>starPep_39250

SCGNLHESCSAHRCCPGLMCFTLPTPICIW

>starPep_39251

SCGNLHESCSAHRCCPGLMCNGEASICVPY

>starPep_39289

SCSGRDSRCXXVCCMGLMCSRGKCVSIYGE

>starPep_39290

SCSGSGYGCKNTPCCAGLTCRGPRQGPICL

>starPep_39294

SCTDDFEPCEAGFENCCSKSCFEFEDVYVC

>starPep_39314

SCVFIPCISAAIGCSCKNKVCYRNGFPCGE

>starPep_39316

SCVFIPCLTTVAGCSCKNKVCYRNGIPCGE

>starPep_39323

SCVYIPCTITALLGCSCKNKVCYNGIPCAE

>starPep_39489

SEQEAGLDTGDGDGDQQYLVRPWLYLWADN

>starPep_39572

SGECNMYGRCPPGYCCSKFGYCGVGRAYCG

>starPep_39648

SGSLSTFFRLFNRGGGWGHFFKKAAHVGKL

>starPep_39668

SGSWLRDVWDWICTVLTDFKTWLQSKLDYK

>starPep_39680

SGWMDYINGFLKGFGGQRTLPTKDYNIPQV

>starPep_39720

SIGSALKKALPVAKKIGKIALPIAKAALPC

>starPep_39742

SIPCETTADCPVAVPPEYYKCMYKVCVLIR

>starPep_39743

SIPCGESCVYIPCITTIVGCSCKNSVCYSN

>starPep_39744

SIPCGESCVYIPCLTTIVGCSCKSNVCYSN

>starPep_39756

SIRMCRREAQLCDPIFQNCCHGLFCVLVCV

>starPep_39893

SLIEESQNQQEKNEQELLELDKWASLWNWF

>starPep_40052

SNPETMVSDVWWRESTENIPRSRFEDPSMW

>starPep_40099

SPHRPRHSRLQREPQVQWLEQQVAKRRTKR

>starPep_40116

SPLQETEEKSRSFKASQAEPLDDSRQLNEV

>starPep_40189

SQAYDPYSNAAQFQLSSQSRGYPYQHRLVY

>starPep_40355

SSLLEKGLDGAKKAVGGLGKLGKDAVEDLE

>starPep_40386

SSPETLISDLLLRESTENIPRSRFEDPSMW

>starPep_40388

SSPETLISDLLMRESTENVPRTRLEDPSMW

>starPep_40389

SSPETLISDLLMRESTGNIPRTRLEDPSMW

>starPep_40390

SSPETMLSDVWWRENTENIPRSRFEDPPMW

>starPep_40463

SVALDPIDISIELNKAKSDLEESKEWIRRS

>starPep_40640

SXFSRTVHNVGNAVRKGIHAGQGVCSGLGL

>starPep_40754

TCFGGTCNTPGCSCDPWPMCSRNGLPVCGE

>starPep_40755

TCFGGTCNTPGCSCDPWPVCSRNGVPVCGE

>starPep_40756

TCFGGTCNTPGCSCETWPVCSRNGLPVCGE

>starPep_40758

TCGETCFGGTCNTPGCTCDPWPICTRDGLP

>starPep_40796

TCSPAGEVCTSKSPCCTGFLCSHIGGMCHH

>starPep_40797

TCSPAGEVCTSKSPCCTGFLCTHIGGMCHH

>starPep_40872

TFFRLFNRSFTQALGKGGGKLFKFLRKHLL

>starPep_40945

TIPCAESCVWIPCTVTALLGCSCKDKVCYN

>starPep_41079

TMCYSHTTTSRAILTNCGENSCYRKSRVHP

>starPep_41278

TSAQITAAVALVEAKQARSDIEKLKEAIRD

>starPep_41504

VAEECEESCEDEEKHCCNTNNGPSCAPQCF

>starPep_41593

VCTPPEGYCTYHRDCCDLYCNKTTNVCLET

>starPep_41694

VFINVKCRGSPECLPKCKEAFGKAAGKCVN

>starPep_41695

VFINVKCRGSPECLPKCKEAIGKAAGKCMN

>starPep_41696

VFINVKCRGSPECLPKCKEAIGKAAGKCVN

>starPep_41763

VGGPQHLCGSHLVDALYLVCGDRGFFYNPR

>starPep_41847

VIGGDECNINEHRFLVALYDGLSGTFLCGG

>starPep_41848

VIGGDECNINEHRFLVALYDPDGFLSGGIL

>starPep_41986

VKPCSEEGQLCDPLSQNCCRGWHCVLVSCV

>starPep_42119

VLSGIMSNLGTVGNMVGGFCCTVYSGCCSE

>starPep_42264

VPVGPALAYACSVMCAKGYDTVVCTCTRRR

>starPep_42617

VSNAATRVCRTGRSRWRDVCRNFMRRYQSR

>starPep_42667

VTEECEENCEEEEKHCCNTNNGPSCAPQCF

>starPep_42792

VWPYGNKKFETLSYLPPLSTGGRIRCMQAM

>starPep_42943

WEAALAEALAEALAEHLAEALAEALEALAA

>starPep_42982

WGATRGCAATCPEAKPRETVECCATDKCNL

>starPep_43161

WKRLWPARILAGHSRRRMRWMVVWRYFAAT

>starPep_43355

WRATRGCAATCPEAKPRETVECCATDKCNL

>starPep_43514

WWEGECYDWLRQCSSPAQCCSGNCGAHSKA

>starPep_43635

WYKHTASPRYHTVGRAAGLLMGLRRSPYMW

>starPep_43872

XHLLREVLELARAEQLAQEAHKNRKLLEII

>starPep_44207

XXIIKXXIKVAKAVAKXIXAIAXGAAANAK

>starPep_44214

XXLLKXXLKVAKAVAKXLXALAXGAAANAK

>starPep_44245

XXXMAKWKLFKKIGIGFKKAAHVGKAALTK

>starPep_44252

XXXXKXXXKVAKAVAKXXXAXAXGAAXNAX

>starPep_44281

YADAIFTNSYRKVLGQLSARKLLQDIMSRQ

>starPep_44388

YECYSTGTFCGVNGGLCCSNLCLFFVCLFS

>starPep_44440

YGGFMKSWEEDRQKPLVTLFKNIINKDEQQ

>starPep_44467

YGRHSHHKEHFKRKCCKRKFHEKHHSHRGY

>starPep_44495

YHWYGYTPQNVIGGGGGRLLRRLLRRLLRK

>starPep_44496

YHWYGYTPQNVIGGGLKLLKKLLKKLLKLL

>starPep_44941

YTSLIHSLIEESQNQQEKNEQELLELDKWA

>starPep_44982

YTSVITIELSNIKENKCNGTDAKVKLIKQE

>starPep_45010

YVEEAVRAALKKEARISTEDTPVNLPSFDC
