# Supplementary material for: Automatic construction of molecular similarity networks for visual graph mining in chemical space of bioactive peptides: an unsupervised learning approach
Source: Sci Rep. 2020 Oct 22;10:18074. doi: 10.1038/s41598-020-75029-1 (PMC7583304; doi:10.1038/s41598-020-75029-1)
Supplement: Supplementary file 8 — Supplementary Information 7. [file 41598_2020_75029_MOESM8_ESM.pdf]

Automatic construction of molecular similarity networks for visual graph mining in chemical space of bioactive peptides: an unsupervised learning approach

Longendri Aguilera-Mendoza[1], Yovani Marrero-Ponce[2,3,4,*], César R. García-Jacas[5], Edgar Chavez[1], Jesus A. Beltran[1], Hugo A. Guillen-Ramirez[6,7], and Carlos A. Brizuela[1,*]

[1]Departamento de Ciencias de la Computación, Centro de Investigación Científica y de Educación Superior de Ensenada (CICESE), Baja California, 22860, Mexico
[2]Universidad San Francisco de Quito, Grupo de Medicina Molecular y Traslacional (MeM&T), Escuela de Medicina, Colegio de Ciencias de la Salud (COCSA), Av. Interoceánica Km 12 1/2 y Av. Florencia, 17-1200-841, Quito, Ecuador.
[3]Grupo GINUMED, Corporacion Universitaria Rafael Nuñez. Facultad de Salud. Programa de Medicina, Cartagena, Colombia.
[4]Unidad de Investigación de Diseño de Fármacos y Conectividad Molecular, Departamento de Química Física, Facultad de Farmacia, Universitat de València, Spain.
[5]Cátedras Conacyt - Departamento de Ciencias de la Computación, Centro de Investigación Científica y de Educación Superior de Ensenada (CICESE), Ensenada, Baja California, Mexico.
[6]Department of BioMedical Research (DBMR), University of Bern, Bern 3008, Switzerland
[7]Department of Medical Oncology, Inselspital, University Hospital and University of Bern 3010, Switzerland
[*]ymarrero77@yahoo.es; cbrizuel@cicese.mx

ABSTRACT

Algorithm SI2-1: Feature ranking and filtering.
Algorithm SI2-2: Feature subset optimization.
Algorithm SI2-3: Parallel construction of the HSP network.

Algorithm SI2-1

Algorithm 1: Feature ranking and filtering

```
input  : A descriptor matrix 𝒟 = [x_ij]_{n×m}, an entropy threshold θ_1, a correlation method corr_method, and correlation-based similarity threshold θ_2
output : A subset F of candidate features
/* Entropy-based filtering                                                      */
F ⟵ {j | j = 1...m} ;                                 /* Initialize the candidate set */
for j = 1 to m do
    f_j.entropy ⟵ H(f_j) ;                                  /* It is defined in Eq. 2 */
    if f_j.entropy < θ_1 then
        F ⟵ F \ {f_j} ;                               /* Removing irrelevant features */
    end
end
rankedFeatures ⟵ Sort features in F by descending order of their entropy values;
/* Correlation-based filtering                                                  */
for j = 1 to sizeOf(rankedFeatures) − 1 do
    for k = j + 1 to sizeOf(rankedFeatures) do
        if corr_method = "pearson" then
            sim(f_j, f_k) ⟵ |ρ(f_j, f_k)| ;                 /* It is defined in Eq. 5 */
        else
            if corr_method = "spearman" then
                sim(f_j, f_k) ⟵ |r_s(f_j, f_k)| ;           /* It is defined in Eq. 6 */
            end
        end
        if sim(f_j, f_k) ≥ θ_2 then
            F ⟵ F \ {f_k} ;                            /* Removing redundant features */
        end
    end
end
```

Algorithm SI2-2

Algorithm 2: Feature subset optimization

```
input  : A candidate feature set F
output : A subset F* of optimized features
/* Second stage: subset optimization                                            */
F* ⟵ F ;                                                  /* Candidate feature set */
best_merit ⟵ Φ(F*) ;                                        /* It is defined in Eq. 7 */
success ⟵ true;
while success do
    success ⟵ false;
    foreach f_j ∈ F* do
        F' ⟵ F* \ {f_j};
        merit ⟵ Φ(F');
        if merit > best_merit then
            best_merit ⟵ merit ;
            best_subset ⟵ F' ;
            success ⟵ true;
        end
    end
    if success then
        F* ⟵ best_subset ;
    end
end
```

Algorithm SI2-3

Algorithm 3: Parallel construction of the HSP network

```
input  : A descriptor matrix 𝒟 = [x_ij]_{n×m}, and distance function d
output : A weighted graph G' = (V, E', w) with a weight w : E' → [0,1]
V ⟵ {i | i = 1...n};
E' ⟵ ∅;
maxDist ⟵ 0;
for u = 1 to n do in parallel
    candidates ⟵ Array[0...n−1] ;                       /* Candidate neighbors to node u */
    cursor ⟵ 0;
    foreach v ∈ V do
        if v ≠ u then
            dist ⟵ d(u,v) ;                             /* Calculating distance d in the space 𝒟 */
            candidates[cursor] ⟵ Candidate(v,dist) ;
            cursor ⟵ cursor + 1 ;
        end
    end
    Sort candidates by ascending order of their distances to node u;
    largeDist ⟵ candidates[n−1].distance;
    cursor ⟵ 0;
    while cursor < n−1 do
        if candidates[cursor] ≠ null then
            v ⟵ candidates[cursor].node ;               /* Nearest candidate node to u */
            dist ⟵ candidates[cursor].distance;
            writeLock() ;                                /* Synchronizing access to a shared resource E' */
            E' ⟵ E' ∪ {(u,v)} ;
            writeUnlock();
            /* Ignoring candidates in the forbidden area for node u */
            for k = cursor + 1 to n−1 do
                if candidates[k] ≠ null then
                    dist ⟵ d(v, candidates[k].node ) ;
                    if dist < candidates[k].distance then
                        candidates[k] ⟵ null ;          /* Ignore candidate nodes closer to v than to u */
                    end
                end
            end
        end
        cursor ⟵ cursor + 1 ;
    end
    writeLock() ;                                        /* Synchronizing access to a shared resource maxDist */
    if largeDist > maxDist then
        maxDist ⟵ largeDist;
    end
    writeUnlock();
end
foreach (u,v) ∈ E' do
    w(u,v) ⟵ sim(u,v) ⟵ 1 − d(u,v)/maxDist ;            /* The similarity defined in Eq. 8 */
end
```
