# Supplementary material for: Automatic construction of molecular similarity networks for visual graph mining in chemical space of bioactive peptides: an unsupervised learning approach
Source: Sci Rep. 2020 Oct 22;10:18074. doi: 10.1038/s41598-020-75029-1 (PMC7583304; doi:10.1038/s41598-020-75029-1)
Supplement: Supplementary file 10 — Supplementary Information 9. [file 41598_2020_75029_MOESM10_ESM.zip › SI4_1-4_Anticancer_peptides/SI4-1_Anticancer_NR98.fasta.docx]

**Supporting Information:**

Automatic construction of similarity networks for visual graph mining in chemical space of bioactive peptides: an unsupervised learning approach

Longendri Aguilera-Mendoza, Yovani Marrero-Ponce*, César R. García-Jacas, Edgar Chavez, Jesus A. Beltran, Hugo A. Guillen-Ramirez, Carlos A. Brizuela*.

Corresponding authors *: Y. Marrero-Ponce: ymarrero@usfq.edu.ec or ymarrero77@yahoo.es; Carlos A. Brizuela: cbrizuel@cicese.edu.mx

**Contents**: Anticancer peptide sequences (FASTA format) that were retrieved from starPepDB (DOI: 10.1093/bioinformatics/btz260) for the purpose of the study.

>starPep_00519

KWKLFKKIPKFLHLAKKF

>starPep_27586

KRKILILILIKRK

>starPep_02244

GRDYRTCLTIVQKLKKMVDKPTQRSVSNAATRVCRTGRSRWRDVCRNFMRRYQSRVTQGL

VAGETAQQICEDLRLCIPSTGPL

>starPep_16624

CKITRCPMIPCYISSPDECLWMDWVTEKNINGHQAKFFACIKRSDGSC

>starPep_01271

FALALKALKKALKKLKKALKKAL

>starPep_27585

KRKILILIKRKRK

>starPep_00004

LLGDFFRKSKEKIGKEFKRIVQRIKDFLRNLVPRTES

>starPep_37975

RRPKGRGKRRREKQRPCDKPRR

>starPep_09360

FAKKLAKLAKKLAKLALAL

>starPep_36961

RKRKLILILIKRKR

>starPep_29863

MAFLKKSLFLVLFLGLVSLSICEKEKRQNEEDEDENEAANHEEGSEEKRGLFDIVKKVVG

AFGSLGKRNDLE

>starPep_06208

KILRGVSKKIMRTFLRRISKDILTGKK

>starPep_13909

YGRKKRRQRRRREADFFWSLCTADMS

>starPep_35821

QMIVIELGTNPLKSSGIENGAFQGMK

>starPep_00025

FKCRRWQWRMKKLGAPSITCVRRAF

>starPep_26684

KISKKIMRTFLRRISKDILTGKK

>starPep_09411

FALALKALKKLAKKLKKLAKKAL

>starPep_37977

RRPKGRGKRRREKQRPSDKPRR

>starPep_09828

GIIKKIIIKKIIIKKIIIKKI

>starPep_11012

KWKKLLKKPPPLLKKLLKKL

>starPep_10206

HHPHGHHPHGHHPHGHHPHG

>starPep_01785

GLICESCRKIIQKLEDMVGPQPNEDTVTQAASRVCDKMKILRGVCKKIMRTFLRRISKDI

LTGKKPQAICVDIKICKE

>starPep_31022

MFTLKKSLLLLFFLGTINLSLCEEERDADEEERRDDPEERAVEVEKRFVDLKKIANIINS

IFGK

>starPep_03498

MNFNKLFVFVALVLAVCIGQSEAGWLKKIGKKIERVGQHTRDATIQTIGVAQQAANVAAT

LKG

>starPep_19622

FIFHIIKGLFHAGKMIHGLVTRRRHGVEELQDLDQRAFEREKAFA

>starPep_00395

YKQCHKKGGHCFPKEKICLPPSSDFGKMDCRWRWKCCKKGSG

>starPep_00663

GFSSIFRGVAKFASKGLGKDLAKLGVDLVACKISKQC

>starPep_01880

KRFKKFFKKVKKSVKKRLKKIFKKPMVIGVTIPF

>starPep_09764

GFWSSVWDGAKNVGTAIIKNAKVCVYAVCVSHK

>starPep_09352

FAKKLAKKLKKLAKKLAKLALALKALALKAL

>starPep_37978

RRPKGRGKRRREKQRPTDCHLCGDAVPRR

>starPep_10623

KKKFPWWWPFKKKCKKKFPWWWPFKKKC

>starPep_00648

GFFGKMKEYFKKFGASFKRRFANLKKRL

>starPep_17857

DKPKKKPPPPAGPPPPPPPPPGPPPPGP

>starPep_05013

ALWKTLLKKVLKAAAKAALNAVLVGANA

>starPep_10598

KILRGVAKKIMRTFLRRISKKILTGKK

>starPep_10624

KKKFPWWWPFKKKKKKFPWWWPFKKKK

>starPep_40171

SPWSSASVTAGDGVDITRIR

>starPep_26658

KILRGVSKKIMRRILTGKK

>starPep_00813

KWKLFKKIPKFLHSAKKF

>starPep_27510

KRFKQDGGWSHWSPWSSC

>starPep_04018

GIIKKIIKKIIKKIIKKI

>starPep_01987

RQIKIWFQNRRMKWKK

>starPep_02206

GLFDIVKKVVGTIAGL

>starPep_16874

CREKAKKLFKKILKKL

>starPep_09371

FAKLFAKLAKKFAL

>starPep_09388

FAKLLAKLAKKFAL

>starPep_07961

WKKWXKKWK

>starPep_06257

KKWWKKWXK

>starPep_23497

GNPANPLNLKKHHGVFCDVCKALVEGGEKVGDDDLDAWLDVNIGTLCWTMLLPLHHECEE

ELKKVKKELKKDIENKDSPDKACKDVDLC

>starPep_08212

ADDKNPLEECFREDDYEEFLEIAKNGLKKTSNPKHIVYPVKPSEQLYEESLRDQLPTSMH

RYPSMIQKIFFAGEYTANAHGWIDSTIK

>starPep_31839

MGSSHHHHHHSSGLVPRGSHMIPVNGVTELEEAASNDTPVAARHEMSMQSWMMPNHIREK

RQSHLSMCSVCCNCCKNYKGCGFCCRF

>starPep_01192

SQLGDLGSGAGQGGGGGGSIRAAGGAFGKLEAAREEEFFYKKQKEQLERLKNDQIHQAEF

HHQQIKEHEEAIQRHKDFLNNLHK

>starPep_01740

GETDPNTQLLNDLGNNMAWGAALGAPGGLGSAALGAAGGALQTVGQGLIDHGPVNVFIPV

LIGPSWNGSGSGYNSATSSSGSGS

>starPep_02255

GYFCESCRKIIQKLEDMVGPQPNEDTVTQAASQVCDKLKILRGLCKKIMRSFLRRISWDI

LTGKKPQAICVDIKICKE

>starPep_00647

GEILCNLCTGLINTLENLLTTKGADKVKDYISSLCNKASGFIATLCTKVLDFGIDKLIQL

IEDKVDANAICAKIHAC

>starPep_02878

SLQPGAPNFPIPGQEKQEGWKFDPSLTRGEDGNTLGSINIHHTGPNHEVGANWDKVIRGP

GKAKPTYSIHGSWRW

>starPep_06783

MASLKKSLFLVLLLGFVSVSICEEEKRQEDEDEHEEEGESQEEGSEEKRGLLSVLGSVAK

HVLPHVVPVIAEHLG

>starPep_29865

MAFLKKSLFLVLFLGLVSLSICEKEKRQNGEDEDENEAANHEEGSEEKRGLFDIVKKVVG

AIGSLGKRNDVE

>starPep_30471

MDSNKDERAYAQWVIIILHNVGSSPFKIANLGLSWGKLYADGNKDKEVYP

>starPep_32716

MNFQQRLQSLWTLARPFCPPLLATASQMQMVVLPCLGFTLLLWSQVSG

>starPep_38527

RTCESQSHRFKGPCARDSNCATVCLTEGFSGGDCRGFRRRCFCTRPC

>starPep_00805

KSCCRNTWARNCYNVCRLPGTISREICAKKCDCKIISGTTCPSDYPK

>starPep_01488

KSCCPNTTGRNIYNTCRLGGGSRERCASLSGCKIISASTCPSDYPK

>starPep_01487

KSCCPNTTGRNIYNTCRFAGGSRERCAKLSGCKIISASTCPSDYPK

>starPep_02704

KSCCPNTTGRNIYNTCRFGGGSREVCARISGCKIISASTCPSDYPK

>starPep_02706

KSCCPNTTGRNIYNTCRFGGGSRQVCASLSGCKIISASTCPSDYPK

>starPep_02292

KSCCPNTTGRNIYNTCRLTGSSRETCAKLSGCKIISASTCPSNYPK

>starPep_01128

KSCCPNTTGRNIYNACRLTGAPRPTCAKLSGCKIISGSTCPSDYPK

>starPep_02703

KSCCKNTTGRNIYNTCRFAGGSRERCAKLSGCKIISASTCPSDYPK

>starPep_19352

FCYWXXXTKKRPKPFQWFWLXKKLMYPTYLKKFQWAVXHL

>starPep_17450

DDDDKRAGSPSGGPFCALARQPLTGSPPNERAFFCSSRDV

>starPep_00225

RRRPRPPYLPRPRPPPFFPPRLPPRIPPGFPPRFPPRFP

>starPep_00096

AGRGKQGGKVRAKAKTRSSRAGLQFPVGRVHRLLRKGNY

>starPep_12663

RRGLFKKLRRKIKKGFKKIFKRLPPVGVGVSIPLAGRR

>starPep_02776

MPRWRLFRRIDRVGKQIKQGILRAGPAIALVGDARAVG

>starPep_00791

ISRLAGLLRKGGEKIGEKLKKIGQKIKNFFQKLVPQPE

>starPep_40880

TFKRKNGSRKNGHRPGGYSLIALGNKKVLKAPYMESI

>starPep_00018

KWKLFKKIEKVGQNIRDGIIKAGPAVAVVGQATQIAK

>starPep_21926

GFSSIFRGVAKFASKGLGKDLAKLGVDLVASKISKQS

>starPep_22827

GLFKKLRRKIKKGFKKIFKRLPPIGVGVSIPLAGKR

>starPep_24565

HGLGHGHEQQHGLGHGHKFKLDDDLEHQGGHVLD

>starPep_21234

GFALAGLARILCLWFREFSGFFRRLNRRFAMRRR

>starPep_18007

DPFFKVPVNKLAAAVSNFGYDLYRVRSSTSPTTN

>starPep_28058

KWKVFKKIEKKWKVFKKIEKAGPKWKVFKKIEK

>starPep_06359

KQLIRFLKRLDRNGGGKLLLKLLKKLLKLLKKK

>starPep_00182

GFFALIPKIISSPLFKTLLSAVGSALSSSGGQE

>starPep_08109

YRWYGYTPQNVIGGGKLLLKLLKKLLKLLKKK

>starPep_07166

NYQWVPYQGRVPYPRGGLLKLLKKLLKKLLKL

>starPep_02587

GIPCAESCVWIPPCTITALMGCSCKNNVCYNN

>starPep_25343

IKYLLVKLQGASQKTITLMLRRNNLYVMGYS

>starPep_06681

LRVRLASHLRKLRKRLLRDADDLQKRLAVY

>starPep_23258

GLPVCGETCVGGTCNTPGCTCSWWPVCTRN

>starPep_00048

DCYCRIPACIAGERRYGTCIYQGRLWAFCC

>starPep_23182

GLPTCGETCFKGKCYTPGCSCSYPICKKD

>starPep_01650

ALWKNMLKGIGKLAGQAALGAVKTLVGAE

>starPep_04706

RRQRRTSKLMKRGGKLAKLAKKLAKLAK

>starPep_22415

GIMDTVKNAAKNLAGQLLDKLKCKITAC

>starPep_08393

ALWKSLLKNVGKAAGKAALNAVTDMVNQ

>starPep_09916

GLLEALAELLEGLRKRLRKFRNKIKEK

>starPep_13296

TRWLWLLRGGLKAAGWGIRAHLNRNQ

>starPep_01168

RFRLPFRRPPIRIHPPPFYPPFRRFL

>starPep_00341

GRRKRKWLRRIGKGVKIIGGAALDHL

>starPep_00126

GLFGKLIKKFGRKAISYAVKKARGKH

>starPep_41038

TLPFAYCNIHQVCHYAQRNDRSYWL

>starPep_13195

TFRAFLSSRLQDLYSIVRRADRAAV

>starPep_00472

GLFSVLGAVAKHVLPHVVPVIAEKL

>starPep_00692

GLFGVLGSIAKHVLPHVVPVIAEKL

>starPep_00260

DTHFPICIFCCGCCHRSKCGMCCKT

>starPep_08186

ACDCRGDCFCGGGGIVRRADRAAVP

>starPep_26305

KCVRQNNKRVCKGLRKRLRKFRNK

>starPep_22967

GLLEALAELLEGRKKRRQRRRPPQ

>starPep_00011

FLPLLAGLAANFLPTIICKISYKC

>starPep_05376

FLKLLKKLAAKFLPTIICKISYKC

>starPep_13353

VALALKALKKLAKKLKKLAKKAL

>starPep_36932

RKKRRQRRRLNLKALLAVAKKIL

>starPep_00759

GWKKWLRKGAKHLGQAAIKGLAS

>starPep_09554

FLPIVAKLLSGLLGRKKRRQRRR

>starPep_09350

FAKKLAKKLKKLAKKLAKLAKKL

>starPep_14993

ALWKEVLKNAGKAALNEINNLVQ

>starPep_10513

KAQIRAMECNILGRKKRRQRRR

>starPep_02607

GLFVGVLAKVAAHVVPAIAEHF

>starPep_09398

FAKLWAKLAFGKGIGKVGKKLL

>starPep_01246

ATPATPTVAQFVIQGSTICLVC

>starPep_00839

RAGLQFPVGRLLRRLLRRLLR

>starPep_00664

GFWGKLFKLGLHGIGLLHLHL

>starPep_01646

AGYLLGKINLKALAALAKKIL

>starPep_01133

KWKLFKKIGIGAVLKVLTTG

>starPep_07818

VAKKLAKLAKKLAKLALAL

>starPep_40173

SPWSSCSVTCGDGVITRIR

>starPep_16456

CGGYCGGWKRKRCTSYRCG

>starPep_40168

SPWSPCSTSCGLGVSTRI

>starPep_12022

NGVQPKYRWWRWWRRWW

>starPep_00316

GLFDIVKKIAGHIVSSI

>starPep_00315

GLFDIVKKIAGHIASSI

>starPep_00314

GLFDIVKKIAGHIAGSI

>starPep_22126

GGVKRFKKFFRKLKKSV

>starPep_05251

FALKALKKLKKALKKAL

>starPep_07876

VNWKKIILGKIIKVVK

>starPep_13342

VAKALAKALLKALKAL

>starPep_04698

RQIRIWFQNRRMRWRR

>starPep_07415

RGDLLRHVVKILSKYL

>starPep_07414

RGDLLRHVVKILEKYL

>starPep_03363

KWFKKIPKFLHLLKKF

>starPep_04309

KQIKIWFQNKKMKWKK

>starPep_00311

GLFDIAKKVIGVIGSL

>starPep_09359

FAKKLAKLAKKLAKAL

>starPep_09330

FAFAKIIAKIAKKII

>starPep_05148

DDALRRLLRRLLRRL

>starPep_43229

WLRRIKAWLRRIKA

>starPep_06441

KWKKLLKKPLKLKL

>starPep_01847

ILPILSLIGGLLGK

>starPep_40437

STNITVTLKKFPL

>starPep_12497

RGVSGHGQHGVHG

>starPep_27634

KRKRKILILIKRK

>starPep_00002

ILPWKWPWWPWRR

>starPep_00350

HGVSGHGQHGVHG

>starPep_05247

FAKLLAKLAKKLL

>starPep_05246

FAKLLAKALKKLL

>starPep_12313

QETFSDLWKLLP

>starPep_39912

SLLSLFRKLIT

>starPep_38095

RRRVVVVVRRR

>starPep_38002

RRRFFFFFRRR

>starPep_13726

WRWRWRWRW

>starPep_27056

KKXWKKWWK

>starPep_00922

AWKLFDDGV

>starPep_10825

KKWXWKK

>starPep_03724

AACARFIDDFCDTLTPNIYRPRDNGQRCYAVNGHRCDFTVFNTNNGGNPIRASTPNCKTV

LRTAANRCPTGGRGKINPNAPFLFAIDPNDGDCSTNF

>starPep_02308

LCPLDVLQLSSELLDIDGNEVEASRILSDITAFGGIRCPLTVVQSRGIGTIISSPYRFIA

EGHPLSLKDMDGWFRVSDDEFNNYK

>starPep_05687

GLICESCRKIIQKLEDMVGPQPNEDTVTQAASQVCDKLKILRGLCKKIMRSFLRRISWDI

LTGKKPQAICVDIKICKEKTGLI

>starPep_33129

MRLLVLSSLLCILLLCFSIFSTEGKRRPAKAWSGRRTRLCCHRVPSPNSTNLKGHHVRLC

KPCKLEPEPRLWVVPGALPQV

>starPep_23916

GSHGAFCHLCEDLIKDGKEAGDVALDVWLDEEIGSRCKDFGVLASECFKELKVAEHDIWE

AIDQEIPEDKTCKEAKLC

>starPep_02877

SLQPGAPKLPYAWSRKQEGWKFDPSLTRGEDGNTLGSINIHHTGRNHEVGANWNKVIRGP

GKAKPTYSIHGSWRW

>starPep_11479

MEKKSFAGLCFLFLVLFVAQECVLQTEAKTCENLADTFRGPCFATGNCDDHCKNKEHLLR

GRCRDDFRCWCTRNC

>starPep_29841

MAFLKKSLFLVLFLGFVSVSICEEEKRQEDEDEHVEEGENQEEGSEEKRGLLSVLGSVAK

HVLPHVVPVIAEHLG

>starPep_29837

MAFLKKSLFLGLFLGFVSVSICEEEKRQEDEDEHDEEGENQEEGSEEKRGLLSVLGSVAK

HVLPHVVPVIAEHLG

>starPep_17285

DAINSPVTCCYTLTSKKISMQRLMSYRRVTSSKCPKEAVIFKTIAGKEICAEPKXXWVQD

SISHLDKKNQXPKP

>starPep_29840

MAFLKKSLFLVLFLALVPLSICEEKKSEEENEEKQEDDQSEEKRGLVTSLIKGAGKLLGG

LFGSVTGGQS

>starPep_03464

MFTLKKSLLLLFFLGTISLSLCEEERNADEEEKRDVEVEKRFLGALFKVASKVLPSVFCA

ITKKC

>starPep_04469

MFTLKKSLLLLFFLGTISSSLCEQERDSDDEDQGEVTEQVVKRLVRGCWTKSYPPKPCFV

RG

>starPep_18139

DSEGWKVQPNINRDQDGNTAGSVRVQKQLGNHEVHAGASRVFSGPNRGGPSYNVGATFNW

>starPep_35918

QPVGINTSTTCCYRFINKKIPKQRLESYRRTTSSHCPREAVIFKTKLDKEICADPTQKWV

>starPep_35428

QDRPKFCYLPADPAECNAYMPRFYYDSASNKCKEFIYGGCRGNANNFKNRAECRHTCVAS

>starPep_03733

ACVNQCPDAIDRFIVKDKGCHGVEKKYYKQVYVACMNGQHLYCRTEWGGPCQL

>starPep_03697

VPAESEAAHLRVRRGFGCPLNQGACHNHCRSIRRRGGYCSGIIKQTCTCYRN

>starPep_00564

YRGGYTGPIPRPPPIGRPPFRPVCNACYRLSVSDARNCCIKFGSCCHLVK

>starPep_16349

CETWRTETTGATGQASSLLSGRLLEQKAASCHNSYIVLCIENSFMTSFSK

>starPep_00740

GRRRRSVQWCAVSQPEATKCFQWQRNMRKVRGPPVSCIKRDSPIQCIQA

>starPep_01641

ADDKNPLEEFRETNYEVFLEIAKNGLKATSNPKRVVIVGAGMAGLSAAY

>starPep_38528

RTCESQSNTFPGICITKPPCRKACISEKFTDGHCSKILRRCLCTKPC

>starPep_36370

RECKTESNTFPGICITKPPCRKACISEKFTDGHCSKILRRCFCTRPC

>starPep_36368

RECKTESNTFPGICITKPPCRKACISEKFSGGDCSKILRRCLCTKPC

>starPep_36369

RECKTESNTFPGICITKPPCRKACISEKFTDGHCRGFRRRCLCTKPC

>starPep_01166

RECKTESNTFPGICITKPPCRKACISEKFTDGHCSKILRRCLCTKPC

>starPep_36367

RECKTESHRFKGPCITKPPCRKACISEKFTDGHCSKILRRCLCTKPC

>starPep_36371

RECKTESNTFPGICITKPPCRKACLTEGFTDGHCSKILRRCLCTKPC

>starPep_02368

QICKAPSQTFPGLCFMDSSCRKYCIKEKFTGGHCSKLQRKCLCTKPC

>starPep_07197

PMPVSQECFETLRGHERILSILRHQNLLKELQDLALQGAKERAHQQ

>starPep_04335

KSCCPSTTGRNIYNTCRLTGSSRETCAKLSGCKIISASTCPSNYPK

>starPep_02293

KSCCPSTTARNIYNTCRLTGASRSVCASLSGCKIISGSTCDSGWNH

>starPep_00116

GIINTLQKYYCRVRGGRCAVLSCLPKEEQIGKCSTRGRKCCRRKK

>starPep_03055

FLPKTLRKFFCRIRGGRCAVLNCLGKEEQIGRCSNSGRKCCRKKK

>starPep_03814

CETPSKHFNGLCIRSSNCASVCHGEHFTDGRCQGVRRRCMCLKPC

>starPep_04761

SKWQHQQDSCRKQLQGVNLTPCEKHIMEKIQGRGDDDDDDDDD

>starPep_18164

DTAVTGLASPLSTGKILDQKAYSCANRLIVLCIENSFMTDARK

>starPep_26158

IYSFDGRDIMTDPSWPQKVIWHGSSPHGVRLVDNYCEAWRTA

>starPep_17915

DLIWKLLVKAQEKFGRGKPSKRVKKMRRQWQACKSSHHHHHH

>starPep_21918

GFRKRFNKLVKKVKHTIKETANVSKDVAIVAGSGVAVGAAM

>starPep_29632

LVPRGSRAGSPSGGPFCALARQPLTGARLMSGLFFALHET

>starPep_04528

MPKWKVFKKIEKVGRNIRNGIVKAGPAIAVLGEAKALG

>starPep_11260

LPKWKVFKKIEKVGRNIRNGIVKAGPAIAVLGEAKALG

>starPep_10231

HSDGIFTDSYSRYRKQMAVKKYLAAVLGRRYRQRFRNK

>starPep_00651

GFGCPNNYQCHRHCKSIPGRCGGYCGGWHRLRCTCYRC

>starPep_07812

TVVRRRGRSPRRRTPSPRRRRSQSPRRRRSQSRESQC

>starPep_38769

RWKIFKKIERVGQNVRDGIIKAGKAIQVLGTAKALGK

>starPep_38770

RWKIFKKIERVGQNVRDGIIKAGPAIQVLGTAKALGK

>starPep_02002

RWKIFKKIEKMGRNIRDGIVKAGPAIEVLGSAKAIGK

>starPep_30757

MFSPILSLEIILALATLQSVFAQPVICTTVGSAAEGS

>starPep_27495

KREDFLDQIIRDFRNFIYQKYRRLRDEFRKLRDILSG

>starPep_03179

GLLSVFKGVLKTAGKNVAKNVAGSLLDQLKCKISGGC

>starPep_21927

GFSSIFRGVAKFASKGLGKKLAKLGVKLVACKISKQC

>starPep_05487

GFGCPFNARRCHRHCRSIRRRAGYCAGRLRLTCTCVR

>starPep_08214

ADDKNPLEECFRETDYEEFLEIARNGLKATSNPKRVV

>starPep_07120

NLVSALIEGRKYLKNVLKKLNRLKEKNKAKNSKENN

>starPep_22064

GGLKKLGKKLEGAGKRVFNAAEKALPVVAGAKALRK

>starPep_21799

GFGCPLNQGACHRHCRSIRRRGGYCSGIIKQTCTCY

>starPep_00023

DHYNCVSSGGQCLYSACPIFTKIQGTCYRGKAKCCK

>starPep_00546

RWKIFKKIEKVGQNIRDGIVKAGPAVAVVGQAATI

>starPep_00361

KWKVFKKIEKMGRNIRNGIVKAGPAIAVLGEAKAL

>starPep_00146

KWKIFKKIEKVGRNIRNGIIKAGPAVAVLGEAKAL

>starPep_00062

GFGALFKFLAKKVAKTVAKQAAKQGAKYVVNKQME

>starPep_02982

CVLIGQRCDNDRGPRCCSGQGNCVPLPFLGGVCAV

>starPep_12735

RRRRRRRRGNLWAAQRYGRELRRMSDEFVDSFKK

>starPep_28062

KWKVFKKIEKMGRNIRNGIVKAGPKWKVFKKIEK

>starPep_00803

KRFKKFFKKLKNSVKKRAKKFFKKPKVIGVTFPF

>starPep_01120

ITSISLCTPGCKTGALMGCNMKTATCNCSIHVSK

>starPep_00511

ITSISLCTPGCKTGALMGCNMKTATCHCSIHVSK

>starPep_01059

GLLRKGGEKIGEKLKKIGQKIKNFFQKLVPQPEQ

>starPep_09784

GGVCPKILKKCRRDSDCPGACICRGNGYCGSGSD

>starPep_22121

GGVCPKILQRCRRDSDCPGACICRGNGYCGSGSD

>starPep_05117

CHTNGGYCVRAICPPSARRPGSCFPEKNPCCKYM

>starPep_00010

ALWKTMLKKLGTMALHAGKAALGAAADTISQGTQ

>starPep_15086

ANIKLSVQMKLFKRHLKWKIIVKLNDGRELSLDA

>starPep_34474

NPEKALEKLIAIQKAIKGMLNGWFTGVGFRRKR

>starPep_04324

KRMGIFHLFWAGLRKLGNLIKNKIQQGIENFLG

>starPep_00484

GLWSKIKEVGKEAAKAAAKAAGKAALGAVSEAV

>starPep_23444

GMWSKIKNAGKAAAKAAAKAAGKAALDAVSEAI

>starPep_00320

GLFDVVKGVLKGVGKNVAGSLLEQLKCKLSGGC

>starPep_01761

GGTIFDCGESCFLGTCYTKGCSCGEWKLCYGTN

>starPep_18008

DPFFKVPVNKLAAVSNFGYDLYRVRSSMSPTTN

>starPep_00249

ACGILHDNCVYVPAQNPCCRGLQCRYGKCLVQV

>starPep_08069

YHWYGYTPQNVIGGGKLLLKLLKKLLKLLKKK

>starPep_01212

VTCYCRRTRCGFRERLSGACGYRGRIYRLCCR

>starPep_04796

THRPPMWSPVWPGGGKLLLKLLKKLLKLLKKK

>starPep_32925

MPRRRRSSSRPVRRRRRPRVSRRRRRRGGRRR

>starPep_00483

GLWSKIKEAAKAAGKAALNAVTGLVNQGDQPS

>starPep_09663

GAFLKCGESCVYLPCLTTVVGCSCQNSVCYRD

>starPep_00089

SWLSKTAKKLENSAKKRISEGIAIAIQGGPR

>starPep_29515

LSSTCILVLVKDILVLVVKEILVLVVKDKPI

>starPep_28608

LHCPALVTYNTDTFESMPNPEGRYTFGASCV

>starPep_01484

KPWRFRRAIRRVRWRKVAPYIPFVVKTVGKK

>starPep_01824

GVIPCGESCVFIPCISSVLGCSCKNKVCYRD

>starPep_02654

GVPCAESCVWIPCTVTALLGCSCKDKVCYLN

>starPep_01091

GTFPCGESCVFIPCLTSAIGCSCKSKVCYKN

>starPep_10080

GTLPCGESCVWIPCISSVVGCACKSKVCYKD

>starPep_01818

GTLPCGESCVWIPCISSVVGCSCKSKVCYKD

>starPep_01427

GSIPCGESCVFIPCISSVIGCACKSKVCYKN

>starPep_01425

GSIPCGESCVFIPCISAIIGCSCSSKVCYKN

>starPep_10029

GSIPCGESCVFIPCISAIIGCSCSNKVCYKN

>starPep_01426

GSIPCGESCVFIPCISAVIGCSCSNKVCYKN

>starPep_02247

GSIPCEGSCVFIPCISAIIGCSCSNKVCYKN

>starPep_23443

GMWSKIKETAMAAAKEAAKAAGKTISDMIKQ

>starPep_01415

GLWDSIKNFGKTIALNVMDKIKCKIGGGCPP

>starPep_01058

GLLPCAESCVYIPCLTTVIGCSCKSKVCYKN

>starPep_02589

GIPCGESCVFIPCTVTALLGCSCKDKVCYKN

>starPep_22430

GIPCAESCVWIPCTITALXGCSCKNNVCYNN

>starPep_02595

GIPCGESCVYIPCTVTALLGCSCKDKVCYKN

>starPep_05617

GIPCAESCVWIPCTVTALLGCSCKDKVCYLD

>starPep_03993

GFLDIIKDTGKEFAVKILNNLKCKLAGGCPP

>starPep_09675

GAVPCGETCVYLPCITPDIGCSCQNKVCYRD

>starPep_16391

CGESCVFIPCISSVIGCACKSKVCYKNGSIP

>starPep_16389

CGESCVFIPCISAIIGCSCSSKVCYKNGSIP

>starPep_16390

CGESCVFIPCISAVIGCSCSNKVCYKNGSIP

>starPep_16336

CEGSCVFIPCISAIIGCSCSNKVCYKNGSIP

>starPep_08395

ALWKTMLKKLGTVALHAGKAALGAVADTISQ

>starPep_12141

PDEDAINDALNKVCSTGRRQRSICKQLLKK

>starPep_12142

PDEDAINNALNKVCSTGRRQRSICKQLLKK

>starPep_27320

KNECLWTDMLSNFGYPGYQSKHYACIRQKG

>starPep_10547

KFFKKLKNSVKKRAKKFFKKPRVIGVSIPF

>starPep_03315

KFFKRLLKSVRRAVKKFRKKPRLIGLSTLL

>starPep_00795

KFFRKLKKSVKKRAKEFFKKPRVIGVSIPF

>starPep_04109

GSVIKCGESCLLGKCYTPGCTCSRPICKKD

>starPep_01828

GVPVCGETCFGGTCNTPGCSCDPWPVCSRN

>starPep_10082

GTSCGETCVLLPCLSSVLGCTCQNKRCYKD

>starPep_09994

GQVWEATATVNAIRGSVTPAVSQFNARTAD

>starPep_23197

GLPVCGETCFGGTCNTPGCSCXTWPVCSRN

>starPep_05737

GLPCGESCVFIPCITTVVGCSCKNKVCYNN

>starPep_01802

GLPVCGETCFGGTCNTPGCSCDPWPMCSRN

>starPep_01800

GLPVCGETCFGGTCNTPGCACDPWPVCTRD

>starPep_01408

GLPVCGETCFGGTCNTPGCSCETWPVCSRN

>starPep_01409

GLPVCGETCFGGTCNTPGCTCDPWPVCTRN

>starPep_01370

GIPCGESCVWIPCLTSAIGCSCKSKVCYRN

>starPep_00307

GIPCGESCVWIPCISSAIGCSCKSKVCYRN

>starPep_02199

GIPCGESCVFIPCLTSAIDCSCKSKVCYRN

>starPep_09845

GIPCGESCVFIPCLTSAIGCSCKSKVCYRN

>starPep_01027

GIPCAESCVWIPCTVTALIGCGCSNKVCYN

>starPep_01030

GIPCGESCVFIPCITGAIGCSCKSKVCYRN

>starPep_01777

GIPCGESCVWIPCITSAIGCSCKSKVCYRN

>starPep_09846

GIPCGESCVWIPCISSAIGCSCXSXVCYRN

>starPep_01029

GIPCGESCVFIPCITAAIGCSCKSKVCYRN

>starPep_22472

GIPCGESCVWIPCISSAIGCSCXSXVCYXN

>starPep_04040

GIPCGXSCVWIPCISSAIGCSCKSKVCYRN

>starPep_22471

GIPCGESCVWIPCISSAIGCSCKSKVCYXN

>starPep_01776

GIPCGESCVFIPCISSVIGCSCSSKVCYRN

>starPep_22357

GIKHILFMAKTKLPRATCTAEIKENCDRKK

>starPep_21211

GETCFGGTCNTPGCTCDPWPVCTRNGLPVC

>starPep_01327

GEFLKCGESCVQGECYTPGCSCDWPICKKN

>starPep_18575

EDMNQKLFDLRGKFKRPPLRRVRMSADAML

>starPep_18616

EEEEEEEEEEKKRLKKIFKKPMVIGVTIPF

>starPep_08662

CGETCVGGTCNTPGCTCSWPVCTRNGLNPV

>starPep_16393

CGESCVFIPCLTSAIDCSCKSKVCYRNGIP

>starPep_05106

CGESCVWIPCISSAIGCSCKSKVCYRNGIP

>starPep_16392

CGESCVFIPCISSVIGCSCSSKVCYRNGIP

>starPep_00021

ACYCRIPACIAGERRYGTCIYQGRLWAFCC

>starPep_40803

TCTLGTCYTAGCSCSWPVCTRNGVPICGE

>starPep_12733

RRRRRRRRGEDIIRNIARHLAQVGDSMDR

>starPep_12732

RRRRRRRRGEDIIRNIARHAAQVGASMDR

>starPep_04632

QRSVSNAATRVSRTGRSRWRDVSRNFMRR

>starPep_01142

LKIPGFVKDTLKKVAKGIFSAVAGAMTPS

>starPep_03423

LKLSPKTKDTLKKVLKGAIKGAIAIASMA

>starPep_04319

KRIRFFERIRDRLRDLGNRIKNRIRDFFS

>starPep_25294

IKLSKETKKNLKKVLKGAIKGAIAVAKMV

>starPep_01112

IKLSPETKDNLKKVLKGAIKGAIAVAKMV

>starPep_25297

IKLSPKTKDNLKKVLKGAIKGAIAVAKMV

>starPep_25236

IKIPSFFRNILKKVGKKAVSLIAGALKQS

>starPep_25295

IKLSKKTKDNLKKVLKGAIKGAIAVAKMV

>starPep_25298

IKLSPKTKKNLKKVLKGAIKGAIAVAKMV

>starPep_25293

IKLSKETKDNLKKVLKGAIKGAIAVAKMV

>starPep_25296

IKLSPETKKNLKKVLKGAIKGAIAVAKMV

>starPep_03287

IKIPSFFRNILKKVGKEAVSLIAGALKQS

>starPep_24427

GYPICGESCVGGICNIPGCSCSWPVCTTN

>starPep_01827

GVPICGETCVGGTCNTPGCSCSWPVCTRN

>starPep_00210

GVPICGETCTLGTCYTAGCSCSWPVCTRN

>starPep_00728

GLPVCGETCVGGTCNTPGCSCSWPVCTRN

>starPep_09942

GLPTCGETCTLGKCNTPKCTCNWPICYKN

>starPep_01410

GLPVCGETCVGGTCNTPGCACSWPVCTRN

>starPep_00726

GLPTCGETCTLGTCYVPDCSCSWPICMKN

>starPep_04076

GLPLCGETCVGGTCNTPGCSCGWPVCVRN

>starPep_01404

GLPICGETCVGGSCNTPGCSCSWPVCTRN

>starPep_09943

GLPVCGETCFTGSCYTPGCSCNWPVCNRN

>starPep_01073

GLPVCGETCTLGTCYTQGCTCSWPICKRN

>starPep_09944

GLPVCGETCVGGTCNTPGCSCSWPVCFRN

>starPep_01071

GLPICGETCVGGTCNTPGCSCSWPVCTRN

>starPep_23271

GLPVCGETCVGGTCYTPGCTCSWPVCTRN

>starPep_23204

GLPVCGETCTLGTCSTQGCTCSWPICKRN

>starPep_09940

GLPTCGETCFKGKCYTPGCSCSYPICKKN

>starPep_23260

GLPVCGETCVGGTCNTPGCTCSYPVCTRN

>starPep_09939

GLPICGETCVGGTCNTPGCFCTWPVCTRN

>starPep_01406

GLPVCGETCAGGTCNTPGCSCSWPICTRN

>starPep_23210

GLPVCGETCVGGTCDTPGCTCSWPVCTRN

>starPep_00727

GLPVCGETCFGGTCNTPGCSCTWPICTRD

>starPep_23268

GLPVCGETCVGGTCNYPGCTCSWPVCTRN

>starPep_01411

GLPVCGETCVGGTCNTPGCGCSWPVCTRN

>starPep_09941

GLPTCGETCTLGKCNTPKCTCNWPICYKD

>starPep_23213

GLPVCGETCVGGTCNSPGCTCSWPVCTRN

>starPep_01407

GLPVCGETCFGGRCNTPGCTCSYPICTRN

>starPep_09871

GKPTCGETCFKGKCYTPGCTCSYPLCKKD

>starPep_09866

GKPICGETCFKGKCYTPGCTCSYPICKKN

>starPep_09878

GKYTCGETCFKGKCYTPGCTCSYPICKKD

>starPep_09865

GKPICGETCFKGKCYTPGCTCSYPICKKD

>starPep_01337

GFMDTAKNVAKNVAVTLIDKLRCKVTGGC

>starPep_01741

GEYCGESCYLIPCFTPGCYCVSRQCVNKN

>starPep_21210

GETCAGGTCNTPGCSCSWPICTRNGLPVC

>starPep_01739

GDACGETCFTGICFTAGCSCNPWPTCTRN

>starPep_01737

GASCGETCFTGICFTAGCSCNPWPTCTRN

>starPep_16413

CGETCVGGTCNTPGCGCSWPVCTRNGLPV

>starPep_16412

CGETCVGGTCNTPGCACSWPVCTRNGLPV

>starPep_08658

CGETCTLGTCYTAGCSCSWPVCTRNGVPI

>starPep_02483

CGETCVGGTCNTPGCTCSWPVCTRNGLPV

>starPep_01230

AIPCGESCVWIPCISTVIGCSCSNKVCYR

>starPep_43981

XLPICGETCVLGTCYTPGCRCQYPICVR

>starPep_43980

XLPICGETCVLGRCYTPNCRCQYPICVR

>starPep_43983

XLPICGETCVLGTCYTPGCSCAYPICVR

>starPep_43982

XLPICGETCVLGTCYTPGCSCAYPICAR

>starPep_07864

VLLVTLTRLHQRGVIYRKWRHFSGRKYR

>starPep_00537

RCLPAGKTCVRGPMRVPCCGSCSQNKCT

>starPep_11149

LGQSAASAHHAYIVLAIENSFMTASKKK

>starPep_03327

KKCKFFCKVKKKIKSIGFQIPIVSIPFK

>starPep_01470

IPCGESCVWIPCITAIAGCSCKNKVCYT

>starPep_25716

IPCGESCVWIPCISGXFGCSCKDKVCYS

>starPep_01118

IPCGESCVWIPCISGMFGCSCKDKVCYS

>starPep_01110

IKIPAVVKDTLKKVAKGVLSAVAGALTQ

>starPep_01109

IKIPAFVKDTLKKVAKGVISAVAGALTQ

>starPep_00720

GLMDTIKGVAKTVAASWLDKLKCKITGC

>starPep_05614

GIMDTIKGAAKDLAGQLLDKLKCKITKC

>starPep_09835

GIMDTVKNAAKNLAGQLLDKLKCSITAC

>starPep_01762

GIACGESCVFLGCFIPGCSCKSKVCYFN

>starPep_00297

GGLRSLGRKILRAWKKYGPIIVPIIRIG

>starPep_05481

GFFGKRKEYFKKFGASFKRRFANLKKRL

>starPep_00098

ALWKDILKNVGKAAGKAVLNTVTDMVNQ

>starPep_08391

ALWKDLLKNVGKAAGKAVLNKVTDMVNQ

>starPep_03758

ALWDTLLKKVLKAAAKAALDAVLVGANA

>starPep_03761

ALWKTLLKKVLKAAAKAALKAVLVGANA

>starPep_43662

XAFCGETCLLGKCYTPGCSCHTGICLK

>starPep_43664

XAFCGETCVLGTCYTPGCSCNFGICLK

>starPep_43663

XAFCGETCLLGTCYTPGCRCTAGICLK

>starPep_00855

SIGAKILGGVKTFFKGALKELASTYLQ

>starPep_07106

MWKWFHNVLSSWQLLADKRPARDYNRK

>starPep_07105

MWKWFHNVLSSGQLLADKWWAWWYNWW

>starPep_07107

MWKWFHNVLSWWWLLADKRPARDYNRK

>starPep_07104

MWKEFHNVLSSGQLLADKRWARWYNRW

>starPep_01521

MRKEFHNVLSSGQLLADKRPARDYNRK

>starPep_33117

MRKWFHNVLSSGQLLADKWPAWDYNWK

>starPep_11918

MRKWFHNVLSSGQLLADKWPAWDYNRK

>starPep_10596

KILRGVAKKILRTFLRRISKDILTGKK

>starPep_06206

KILRGVAKKIMRTFLRRISKDILTGKK

>starPep_26183

KAKAKAVSRSARAGLQFPVGRIHRHLK

>starPep_24842

HTHQDFQPVLHLVALNTPLSGGMRGIR

>starPep_00136

GRFKRFRKKFKKLFKKLSPVIPLLHLG

>starPep_23735

GRFKRFRKKLKRLWHKVGPFVGPILHY

>starPep_00675

GIGTKILGGVKTALKGALKELASTYAN

>starPep_01769

GIGGALLSAGKSALKGLAKGLAEHFAN

>starPep_00301

GIGGVLLSAGKAALKGLAKVLAEKYAN

>starPep_00674

GIGGKILSGLKTALKGAAKELASTYLH

>starPep_03125

GIFPIFAKLLGKVIKVASSLISKGRTE

>starPep_22216

GIFPIFAKLLGKVIKVASSLISKGRTK

>starPep_21198

GEILCNLCTGLINTLENLLTTKRKRQQ

>starPep_43791

XDCGETCVLGTCYTPGCSCSAYPLCV

>starPep_40276

SRTVRKTSRLWSSLSLNTCNNVHSKS

>starPep_34689

NVLLSPLSVATALSALSLGAEQRTES

>starPep_04353

KWKSFAKTFKSAKKTVLHTALKAISS

>starPep_03396

KWKSFLKTFKSAAKTVLHTALKAISS

>starPep_06489

KWKSFLKTFKSAKKTVAHTALKAISS

>starPep_01903

KWKSFLKTFKSAKKTVLHTALKAISS

>starPep_02715

KWKSFLKTFKSLKKTVLHTLLKLISS

>starPep_02306

KWKSFLKTFKSLKKTVLHTLLKAISS

>starPep_02304

KWKSFLKTFKSAKKTVLHTLLKAISS

>starPep_06488

KWKSFLKTFKSAKKTVAHTAAKAISS

>starPep_02714

KWKSFLKTFKSLKKTVLHTALKAISS

>starPep_06490

KWKSFLKTFKSALKTVLHTALKAISS

>starPep_28031

KWKSFLKTFKSAEKTVLHTALKAISS

>starPep_06468

KWKSFAKTFKSAKKTVAHTALKAISS

>starPep_28039

KWKSFLKTFKSAKKTVLHTAAKAISS

>starPep_00807

KSSAYSLQMGATAIKQVKKLFKKWGW

>starPep_25166

IIGAVLKVLTTGLPALISWIKRKRQQ

>starPep_24834

HTASDAAAAAALTAANAAAAAAASMA

>starPep_24256

GVWGIAKIAGKVLGNILPHVFSSNQS

>starPep_00205

GLLQTIKEKLESLESLAKGIVSGIQA

>starPep_05678

GLFGKLIKKKGRKAISYAVKKARGKH

>starPep_09889

GLFGKLIKKFARKAISYAVKKARGKH

>starPep_05679

GLFGKLQKKFGRKAISYAVKKARGKH

>starPep_05677

GLFGKLIKKFLRKAISYAVKKARGKH

>starPep_09823

GIGVLLSAGKAALKGLAKVLAEKYAN

>starPep_22239

GIGAVLKVLTTGLPALKSWIKRKRQQ

>starPep_00000

GIGAVLKVLTTGLPALISWIKRKRQQ

>starPep_00657

GFLGILFHGVHHGRKKALHMNSERRS

>starPep_05256

FAVGLRAIKRALKKLRRGVRKVAKDL

>starPep_00569

AGWGSIFKHIFKAGKFIHGAIQAHND

>starPep_41838

VIFEWTLLQVLSESDQDQSLEVFLT

>starPep_00545

RWGKWFKKATHVGKHVGKAALTAYL

>starPep_11901

MRGIRGADFQAFQQARAVGLAGTFR

>starPep_03342

KLKNFAKGVAQSLLNKASCKLSGQC

>starPep_06287

KLKNFAIGVAQSLLNKASCKLSGQC

>starPep_01874

KLCGETCFKFKCYTPGCSCSYPFCK

>starPep_02690

KKLALALAKKWLPLAKKLALALAKK

>starPep_02693

KKLALLALKKWLPALKKLALLALKK

>starPep_02692

KKLALLALKKWLLALKKLALLALKK

>starPep_02689

KKLALALAKKWLALAKKLALALAKK

>starPep_02691

KKLALHALKKWLHALKKLAHLALKK

>starPep_04261

KKALAKALKHWLPALHKLAKALAKK

>starPep_03325

KKALAHALKKWLPALKKLAHALAKK

>starPep_04262

KKALKHALAKWLPALKALAHKLAKK

>starPep_10235

HSHRDFQPVLHLVALNSPLSGGMRG

>starPep_05855

GWRKWIKKATHVGKHIGKAALDAYI

>starPep_01097

GWKKWFNRAKKVGKTVGGLAVDHYL

>starPep_00494

GWGSIFKHGRHAAKHIGHAAVNHYL

>starPep_05849

GWKDWFRKAKKVGKTVGGLALNHYL

>starPep_00008

GWGSFFKKAAHVGKHVGKAALTHYL

>starPep_01098

GWKSVFRKAKKVGKTVGGLALDHYL

>starPep_23911

GSGSGSGSLKKIFKKPMVIGVTIPF

>starPep_10026

GSEGPLKPGARIFSFDGKDVLRHPT

>starPep_01068

GLLSVLGSVAQHVLPHVVPVIAEHL

>starPep_00129

GLLSVLGSVAKHVLPHVVPVIAEKL

>starPep_00719

GLLSVLGSVVKHVIPHVVPVIAEHL

>starPep_00334

GLMSSIGKALGGLIVDVLKPKTPAS

>starPep_05697

GLKKWFKKAVHVGKKVGKVALNAYL

>starPep_05620

GIRKWFKKAAHVGKKVGKVALNAYL

>starPep_05619

GIRKWFKKAAHVGKEVGKVALNACL

>starPep_05588

GIGSAILSAGKSIIKGLAKGLAEHF

>starPep_05460

GCKKWFKKAAHVGKNVGKVALNAYL

>starPep_01716

FIHHIIGGLFSAGKAIHRLIRRRRR

>starPep_00051

FFGWLIKGAIHAGKAIHGLIHRRRH

>starPep_09303

ETWRTEAPSATGQASSLLGGRLLGQ

>starPep_08183

AAVPIVNLKDELLFPSWEALFSGSE

>starPep_14350

ADMDFTGIAESIIKKIKETNAKPPA

>starPep_41266

TRSRWRRFIRGAGRFARRYGWRIA

>starPep_41082

TMPFLFCNVNDCNFASRNDYSYWL

>starPep_13293

TRSSRAGLQWPVGRVHRLLRKGGC

>starPep_02849

RKGWFKAMKSIAKFIAKEKLKEHL

>starPep_33981

NGRKISLDLRAPLYKKIIKKLLES

>starPep_33980

NGRKACLNPASPIVKKIIEKMLNS

>starPep_33979

NGREACLDPEAPMVQKIVQKMLKG

>starPep_33964

NGKQVCLDPEAPFLKKVIQKILDS

>starPep_29425

LRSRGELVAKFLAGEQSPEDYVAE

>starPep_02289

KRKCPKTPFDNTPGAWFAHLILGC

>starPep_26099

IWLTALKFLGKNLGKLAKQQLAKL

>starPep_10222

HPTWPQKSVWHGSDPNGRRLTESY

>starPep_23769

GRKKRRQRRRGALWKSLLKNVGKA

>starPep_00469

GLFKVLGSVAKHLLPHVVPVIAEK

>starPep_00323

GLFKVLGSVAKHLLPHVAPVIAEK

>starPep_00686

GKGRWLERIGKAGGIIIGGALDHL

>starPep_02134

FLPLIAGVAANFLPKIFCLISKKC

>starPep_02141

FLPVIASVAAKVLPKVFCFITKKC

>starPep_00628

FLPLAVSLAANFLPKLFCKITKKC

>starPep_02135

FLPLIAGVAASILPKIFCFITKKC

>starPep_00634

FLPVIAGVAANFLPKLFCAISKKC

>starPep_00428

FLPIIAGAAAKVVQKIFCAISKKC

>starPep_00968

FLPIIAGVAAKVLPKIFCAISKKC

>starPep_20017

FLPILASLAAKFGPKLFSLVTKKS

>starPep_00429

FLPIIAGIAAKFLPKIFCTISKKC

>starPep_00619

FLPIIAGAAAKVVEKIFCAISKKC

>starPep_00420

FLGLLFHGVHHVGKWIHGLIHGHH

>starPep_20018

FLPILASLAAKFGPKLFXLVTKKX

>starPep_00055

FLPILASLAAKFGPKLFCLVTKKC

>starPep_00423

FLPAIVGAAAKFLPKIFCAISKKC

>starPep_03042

FLGALFKVASKVLPSVKCAITKKC

>starPep_00419

FLGALIKGAIHGGRFIHGMIQNHH

>starPep_02124

FLGAIAGVAAKFLPKVFCFITKKC

>starPep_19502

FFPGIIKVAGAILPTAICAITKRC

>starPep_19503

FFPGIIKVASAILPTAICAITKRC

>starPep_01265

DSMGAVKLAKLLIDKMKCEVTKAC

>starPep_14847

ALARQPLTGSPPNERAFFCSSLRR

>starPep_08389

ALSKALSKALSKALSKALSKALSK

>starPep_13351

VALALKALKKALKKLKKALKKAL

>starPep_13345

VAKKFAKKFKKFAKKFAKFAFAF

>starPep_36693

RIIDLLWRVWRPWKPKFVTVWVR

>starPep_36691

RIIDLLWRVRRPWWPKFVTVWVR

>starPep_00224

RIIDLLWRVRRPQKPKFVTVWVR

>starPep_36690

RIIDLLWRVRRPWKPKFVTVWVR

>starPep_36689

RIIDLLWRVRRPQWPKFVTVWVR

>starPep_36694

RIIDLLWRVWRPWWPKFVTVWVR

>starPep_36692

RIIDLLWRVWRPQKPKFVTVWVR

>starPep_33960

NGKEICLDPEAPFLKKVIQKILD

>starPep_11029

KWKLFKKKTKLFKKFAKKLAKKL

>starPep_10978

KTKLFKKFAKKLAKKLKKLAKKL

>starPep_10591

KIAKVALAKLGIGAVLKVLTTGL

>starPep_00760

GWRTLLKKAEVKTVGKLALKHYL

>starPep_02606

GLFVGLAKVAAHNNPAIAEHFQA

>starPep_00321

GLFGVLAKVASHVVPAIAEHFQA

>starPep_09816

GIGKFLHSAKKWGKAFVGQIMNC

>starPep_00001

GIGKFLHSAKKFGKAFVGEIMNS

>starPep_01013

GIGKFLHAAKKFAKAFVAEIMNS

>starPep_02191

GIGKFLHSAKKFAKAFVAEIMNS

>starPep_02121

FIHHIIGGLFSVGKHIHSLIHGH

>starPep_09353

FAKKLAKKLKKLAKKLIGAVLKV

>starPep_09401

FALAAKALKKLAKKLKKLAKKAL

>starPep_05250

FALALKALKKLLKKLKKLAKKAL

>starPep_02507

FAKKFAKKFKKFAKKFAKFAFAF

>starPep_17703

DGRELCLDPKENWVQRVVEKFLK

>starPep_00570

AIGSILGALAKGLPTLISWIKNR

>starPep_08303

AIGKFLHSAKKFGKAFVGEIMNS

>starPep_14990

ALWKDILKNLLKAALNEINQIVQ

>starPep_14994

ALWKKILKNAGKAALNKINQIVQ

>starPep_00577

ALWKDILKNAGKAALNEINQIVQ

>starPep_44212

XXLIXVWAXGFXXAXXLFXGIG

>starPep_43905

XIXIXIXRPVYXPRPRPPHPRL

>starPep_44200

XWXWXWXRPVYXPRPRPPHPRL

>starPep_41343

TTITGKKCQSWAAMFPHRHSKT

>starPep_13286

TRRKFWKKVLNGALKIAPFLLG

>starPep_13155

TAGIKLTVPIEKFPVTTQTFWG

>starPep_37973

RRPKGRGKRAAAKQRPSDKPRR

>starPep_37969

RRPAAAGKRRREKQRPSDKPRR

>starPep_12723

RRRQRRKKRGGGDTRLNTVWMW

>starPep_12534

RKAFRWAWRMLKKAAPSITCVR

>starPep_36311

RCPGRTRQIGTIFPGRIKCRSW

>starPep_35471

QEPHRHSIFTPQTNPRADLEKN

>starPep_33962

NGKKACLNPASPMVQKIIEKIL

>starPep_32937

MPTWAWWLFLVLLLALWAPARG

>starPep_01520

MQFITDLIKKAVDFFKGLFGNK

>starPep_32958

MQFITDLIKKAVDVFKGLFGNK

>starPep_32957

MQFITDLIKKAVDFFKGLFDNK

>starPep_11215

LLGAALSALSSVIPSVISWFQK

>starPep_26917

KKLIKVFAKGWKKAKKLFKGIG

>starPep_26916

KKLIKVFAKGFKKAKKLWKGIG

>starPep_26918

KKLIKVWAKGFKKAKKLFKGIG

>starPep_26717

KKAAKAWAKGAKKAKKLAKGAG

>starPep_10496

KAAKKAWKAWKKAAKAAWKKAA

>starPep_26494

KGIRGYKGGYCKGAFKQTCKCY

>starPep_24402

GYCSWYRGWAPPDKSIINATDP

>starPep_05790

GRKKRRQRRRGGWMWVTNLRTD

>starPep_02610

GLLRRLRDFLKKIGEKFKKIGY

>starPep_00066

GIGKFLKKAKKFGKAFVKILKK

>starPep_01019

GIKCRFCCGCCTPGICGVCCRF

>starPep_09817

GIGKFLKKAKKFAKAFVKIINN

>starPep_09765

GFXGXKKXGXFXGXGKKXKKKK

>starPep_02522

FIHHIIGWISHGVRAIHRAIHG

>starPep_02516

FFGRLKSVWSAVKHGWKAAKSR

>starPep_09349

FAKKLAKKLKKLAKKLAKKWKL

>starPep_00005

FFHHIFRGIVHVGKTIHRLVTG

>starPep_09335

FAKAIAKIAFGKGIGKVGKKLL

>starPep_18824

EKSSRPEFYKVILGAHEEYIRG

>starPep_18760

EIPSCESSASPDQSDSSVPPEE

>starPep_02101

DSIRDVSPTFNKIRRWFDGLFK

>starPep_17705

DGRKICLDPDAPRIKKIVQKKL

>starPep_41197

TQQAFQKFLAAVTSALGKQYH

>starPep_39822

SKRKSRPVSVKTFEDIPLEEP

>starPep_00381

RWCVYAYVRVRGVLVRYRRCW

>starPep_00846

RSTEDIIKSISGGGFLNAMNA

>starPep_37972

RRPKGRAMRREKQRPSDKPRR

>starPep_29189

LPGLTGSKGVRGISGLPGFSG

>starPep_04365

LALERRSGWLRLFGLKPRRKH

>starPep_11046

KWWKKAAKAAKKAAKAAKKWA

>starPep_10497

KAAKKAWKWAKKAAKWAKKAA

>starPep_10495

KAAKKAWKAAKKAWKAAKKAA

>starPep_10494

KAAKKAWKAAKKAAKWWKKAA

>starPep_10499

KAAKKWAKAAKKWAKAWKKAA

>starPep_10498

KAAKKWAKAAKKAAKAWKKAA

>starPep_10492

KAAKKAAKAAKKAAKAAKKAA

>starPep_10500

KAAKKWAKAWKKAAKAWKKAA

>starPep_10273

IELLQARGGCXGGRRRRRRRR

>starPep_00713

GLLGLLGSVVSHVLPAITQHL

>starPep_01056

GLLGLLGSVVSHVVPAIVGHF

>starPep_02568

GFIFHIIKGLFHAGKMIHGLV

>starPep_02535

FLHHIVGLIHHGLSLFGDRAD

>starPep_09404

FALAKLAKKAKAKLKKALKAL

>starPep_18829

EKYEGKISKTMSGLDCQAWDS

>starPep_16808

CNGRCGGKLAKLAKKLAKLAK

>starPep_08569

AWKKWAKAWKWAKAKWWAKAA

>starPep_08184

AAWKWAWAKKWAKAKKWAKAA

>starPep_44199

XWXWXRPVYXPRPRPPHPRL

>starPep_43906

XIXIXRPVYXPRPRPPHPRL

>starPep_44335

YCNINEVCHYARRNDKSYWL

>starPep_02429

VYINKLTPPCGTMYYACEAV

>starPep_40154

SPSTHPNEGLEENYCRNPDN

>starPep_40169

SPWSQCSVRCGRGQRSRQVR

>starPep_40165

SPWSKCSAACGQTGVQTRTR

>starPep_39111

RYRRKKKMKKALQYIKLLKE

>starPep_12454

RDVCRNFMRRYQSRVIQGLV

>starPep_01971

QSHLSLCRWCCNCCRSNKGC

>starPep_34876

PGLKGKRGDSGSPATWTTRG

>starPep_35021

PMLRVRLASHLRKLRKRLLR

>starPep_12079

NRFTARFRRTPWRLCLQFRQ

>starPep_12023

NHFTLKCPKTALTEPPTLAY

>starPep_04575

NFAEIFAAVNKLIKQGVVKG

>starPep_29236

LPRFSTMPFIYCNINEVCHY

>starPep_29405

LRRFSTMPFMFCNINNVCNF

>starPep_11176

LKCNKLVPLFYKTCPAGKNL

>starPep_28218

KYLNFAKWLKGANLAKYANA

>starPep_11016

KWKLFKKALKKLKKALKKAL

>starPep_11025

KWKLFKKIGIGKFLHSATTF

>starPep_11010

KWKKFLKIGIGKFLHLAKKF

>starPep_02299

KWKLFKKIGPGKFLHSAKKF

>starPep_11015

KWKLFAKIGIGKFLHLAKKF

>starPep_00517

KWKLFKKIGIGKFLHSAKKF

>starPep_11021

KWKLFKKIGIGAVLKVLKKG

>starPep_11019

KWKLFKKIGIGAFLHLAKKF

>starPep_11023

KWKLFKKIGIGKFLHLAKKF

>starPep_11020

KWKLFKKIGIGAFLHSAKKF

>starPep_01492

KTCENLADTFRGPCFATSNC

>starPep_27763

KSVRGKGKGQKRKRKKSRYK

>starPep_04318

KRIGLIRLIGKILRGLRRLG

>starPep_10629

KKKKGFXGXKXGXFXGXGKX

>starPep_01866

KIKIPWGKVKDFLVGGMKAV

>starPep_10463

ITCPQVTQSLAPCVPYLISG

>starPep_06127

IVPFLLGMVPKLVCLITKKC

>starPep_00353

ILGPVISTIGGVLGGLLKNL

>starPep_00775

ILGPVLGLVSDTLDDVLGIL

>starPep_10217

HLRRINKLLTRIGLYRHAFG

>starPep_10156

HARIKPTFRRLKWKYKGKFW

>starPep_24426

GYNYAKKLANLAKKFANALW

>starPep_02584

GILSKLGKALKKAAKHAAKA

>starPep_22358

GIKIAKKAITIAKKIAKIYW

>starPep_09767

GFXGXRXGXFXGXGRXRRRR

>starPep_09766

GFXGXKXGXFXGXGKXKKKK

>starPep_21045

GCRRLCYKQRCVTYCRGPPR

>starPep_20638

GAKALTKAATAFTKFYKTIW

>starPep_20666

GANAAKKFATIAKKFINYLW

>starPep_20707

GATYAKKIIKTITKIATTAW

>starPep_20667

GANAAKKLATFAKKIFTAYW

>starPep_20670

GANLAKKFYTYINKFINYAW

>starPep_00282

FLSLALAALPKFLCLVFKKC

>starPep_20041

FLPKLLAGLPSFLCLVFKKC

>starPep_00637

FLSLALAALPKLFCLIFKKC

>starPep_03060

FLPLLLSALPSFLCLVFKKC

>starPep_20074

FLPLLLAGLPSFLCLVFKKC

>starPep_20073

FLPLLLAGLPKFLCLVFKKC

>starPep_00614

FLGGLIKIVPAMICAVTKKC

>starPep_03897

FCTCNVKGFNAKNKRGIIYP

>starPep_05242

FAGLAANFLPTIICKISYKC

>starPep_18695

EGLPGPQGPKGFPGLPGLTG

>starPep_16614

CKGRGKRCREKQRPSDKPRR

>starPep_15451

ARPAKAAATQKKVERKAPDA

>starPep_08170

AAKKWAKAKWAKAKKWAKAA

>starPep_04919

ADDINPKEECFFEDDYYEFE

>starPep_04920

ADDKNPLEECFCEDDDYCEG

>starPep_43942

XKPRXYTPRPTSHPRPIRV

>starPep_41629

VDKPPYLPRPRPXRXIYNX

>starPep_41627

VDKPPYLPRPRPPRXIYNX

>starPep_41630

VDKPPYLPRPRPXRXXYNX

>starPep_41327

TSWSPCSASCGGGHYQRTR

>starPep_41628

VDKPPYLPRPRPPRXXYNX

>starPep_40852

TEWSVCNSRCGRGYQKRTR

>starPep_41002

TKPRKTKPRKTKPRKTKPR

>starPep_40850

TEWSACNVRCGRGWQKRSR

>starPep_41205

TQWTSCSKTCNSGTQSRHR

>starPep_40213

SQWSPCSRTCGGGVSFRER

>starPep_40166

SPWSPCSGNCSTGKQQRTR

>starPep_40164

SPWDICSVTCGGGVQKRSR

>starPep_40174

SPWTKCSATCGGGHYMRTR

>starPep_39849

SKWSECSRTCGGGVKFQER

>starPep_39209

SAWRACSVTCGKGIQKRSR

>starPep_39501

SEWSDCSVTCGKGMRTRQR

>starPep_39593

SGGYCGGWHRLRCTSYRSG

>starPep_39171

SAPFIECHGRGTCNYYANS

>starPep_36506

RGFTKMPHVQIHTEASESL

>starPep_07469

RILRGVSRRIMRRILTGRR

>starPep_35930

QPWSQCSATCGDGVRERRR

>starPep_35925

QPWGTCSESCGKGTQTRAR

>starPep_34952

PKILNKILGKILRLAAAFK

>starPep_06981

MPKEKVFLKIEKMGRNIRN

>starPep_29247

LPVFSTLPFAYCNIHQVCH

>starPep_11276

LPRRNRWSKIWKKVVTVFS

>starPep_11022

KWKLFKKIGIGKFKLAKKF

>starPep_26524

KGRGKRRRECQRPSCKPRR

>starPep_26495

KGIRGYKGGYKGAFKQTKY

>starPep_26523

KGRGKRRRCKQRPSDCPRR

>starPep_23644

GPWGDCSRTCGGGVQFSSR

>starPep_23645

GPWGPCSGSCGPGRRLRRR

>starPep_23643

GPWERCTAQCGGGIQARRR

>starPep_23641

GPWEDCSVSCGGGEQLRSR

>starPep_23642

GPWEPCSVTCSKGTRTRRR

>starPep_01055

GLKKLLGKLLKKLGKLLLK

>starPep_22682

GKPRXYSPRPTSHPRPIRV

>starPep_00124

GKPRPYSPRPTSHPRPIRV

>starPep_05498

GFKDLLKGAAKALVKAVLF

>starPep_05499

GFKDLLKGAAKALVKTVKF

>starPep_05497

GFKDLLKGAAKALKKTVLF

>starPep_05501

GFKDLLKKAAKALVKTVLF

>starPep_00185

GFKDLLKGAAKALVKTVLF

>starPep_05500

GFKDLLKGAKKALVKTVLF

>starPep_03114

GFKKLLKGAAKALVKTVLF

>starPep_20132

FLSMIPHIVSGVAALAKHL

>starPep_00640

FLSLIPHIVSGVASIAKHF

>starPep_20127

FLSLIPHIVSGVASLAKHF

>starPep_20129

FLSLIPKIAGGIAALAKHL

>starPep_09578

FLSLIPKIATGIAALAKHL

>starPep_20122

FLSLIPAAISAVSALANHF

>starPep_19813

FLFKLIPKVIKGLVKAIRK

>starPep_19811

FLFKLIPKAIKGLVKAIRK

>starPep_03040

FLFSLIPSAIAGLVSAIRN

>starPep_03041

FLFSLIPSVIAGLVSAIRN

>starPep_00605

FFRLLFHGVHHGGGYLNAA

>starPep_19552

FFSMIPKIAGGIASLVKNL

>starPep_00606

FFRLLFHGVHHVGKIKPRA

>starPep_09354

FAKKLAKKLKKLAKLALAK

>starPep_19340

FCNINNVCNFASRNDYSYW

>starPep_16455

CGGYCGGWHRLRCTSYRCG

>starPep_16457

CGGYSGGWHRLRSTSYRCG

>starPep_15907

AWYRGAAPPKQEFLDIEDP

>starPep_15619

ASWSACSVSCGGGARQRTR

>starPep_15695

ATPFIECSGARGTCHYFAN

>starPep_14190

AAPFLECQGRQGTCHFFAN

>starPep_03749

AKKVFKRLEKLFSKIWNDK

>starPep_03751

AKKVFKRLEKSFSKIQNDK

>starPep_03750

AKKVFKRLEKLFSKIWNWK

>starPep_03748

AKKVFKRLEKLFSKIQNWK

>starPep_03752

AKKVSKRLEKLFSKIQNDK

>starPep_03685

VDKPPYLPRPRPPRRIYNR

>starPep_04923

ADDRNPLEEFRENNYEEFL

>starPep_44028

XNNRPVYIPRPRPPHPRL

>starPep_03716

XCRRLCYKQRCVTYCRGR

>starPep_43000

WGRAFRRGVRRLARGGRR

>starPep_43219

WLRAFRRLVRRLARGLRR

>starPep_43003

WGRAFSAGVHRLARGGRG

>starPep_43001

WGRAFRRLVRRLARGLRR

>starPep_43004

WGRAFSRGVRRLARGGRG

>starPep_43220

WLRAFRRLVRRLARLLRR

>starPep_43002

WGRAFSAGVHRLANGGNG

>starPep_42983

WGEAFSAGVHRLANGGNG

>starPep_41328

TSWSQCSKTCGTGISTRV

>starPep_40853

TEWTACSKSCGMGFSTRV

>starPep_40851

TEWSACSKTCGMGISTRV

>starPep_41021

TKWTPCSRTCGMGISNRV

>starPep_40725

TAWGPCSTTCGLGMATRV

>starPep_00849

SAVGRHGRRFGLRKHRKH

>starPep_00151

RRWCFRVCYKGFCYRKCR

>starPep_00043

RRWCFRVCYRGFCYRKCR

>starPep_37859

RRGCFRVCYRGFCFQRCR

>starPep_00020

RGGRLCYCRRRFCVCVGR

>starPep_00372

QCRRLCYKQRCVTYCRGR

>starPep_12135

PAWRKAFRWAKRMLKKAA

>starPep_12133

PAWRKAFRKAWRMLKKAA

>starPep_34789

PAWRHAFHWAWHMLHKAA

>starPep_12129

PAWFKARRWAWRMKKLAA

>starPep_07169

PAWRKAFRWAWRMLKKAA

>starPep_12130

PAWFKARRWAWRMLKKAA

>starPep_12136

PAWRKAFRWAWRMKKLAA

>starPep_04599

PEWFKCRRWQWRMKKLGA

>starPep_12131

PAWRKAARWAWRMLKKAA

>starPep_12128

PAWAKAFRAAARMKLKAA

>starPep_12137

PAWRKARRWARRMKKLAA

>starPep_12132

PAWRKAFRAAWRMLKKAA

>starPep_34790

PAWRKAFRWAWHMLHHAA

>starPep_12134

PAWRKAFRWAARMLKKAA

>starPep_12138

PAWRKARRWAWRMKKLAA

>starPep_34788

PAWHHAFHWAWRMLKKAA

>starPep_12110

PAARKAARWAWRMLKKGA

>starPep_12111

PAARKAFRWAWRMLKKAA

>starPep_00524

LLGMIPLAISAISALSKL

>starPep_02300

KWKLFKKISKFLHLAKKF

>starPep_28181

KXRRYXYRQRXVTYXRGR

>starPep_06446

KWKKLLKKXLKLLKKLLK

>starPep_06448

KWKKLLXKLLXLLKKLLK

>starPep_06447

KWKKLLKKXLKLXKKLLK

>starPep_06440

KWKKLLKKLXKLLKKLLK

>starPep_06439

KWKKLLKKLLXLLKKLLK

>starPep_03371

KWKLFKKILKFLHLAKKF

>starPep_06433

KWKKLLKKLLKLLKKLLK

>starPep_06411

KVKVKVKVPPTKVKVKVK

>starPep_00360

KNLRRITRKIIHIIKKYG

>starPep_00359

KNLRRIIRKGIHIIKKYG

>starPep_00512

KNLRRIIRKIIHIIKKYG

>starPep_26299

KCRRYCYRQRCVTYCRGR

>starPep_26297

KCRRLCYRQRCVTYCRGR

>starPep_24385

GXRRLXYKQRXVTYXRGR

>starPep_00758

GWKKWFTKGERLSQRHFA

>starPep_23640

GPWAPCSASCGGGSQSRS

>starPep_00076

GLLGPLLKIAAKVGSNLL

>starPep_05631

GKKLKKIGQKIKNFFQKL

>starPep_01002

GICRCICGRGICRCICGR

>starPep_00115

GFVDFLKKVAGTIANVVT

>starPep_21047

GCRRLCYRQRCVTYCRGR

>starPep_21043

GCRRLCWRQRCVTWCRGR

>starPep_21044

GCRRLCYKQRCVTWCRGR

>starPep_05469

GEKLKKIGQKIKNFFKKL

>starPep_05468

GEKLKKIGQKIKKFFQKL

>starPep_21048

GCRRWCYKQRCVTYCRGR

>starPep_21042

GCRRLCWKQRCVTYCRGR

>starPep_21046

GCRRLCYKQRCVTYCRGR

>starPep_21038

GCRALCYKQRCVTYCRGA

>starPep_05467

GEKLKKIGKKIKNFFQKL

>starPep_03083

FPFSLIPHAIGGLISAIK

>starPep_02527

FLFSLIPKAIGGLISAFK

>starPep_01283

FLFSLIPHAIGGLISAFK

>starPep_00932

ECRRLCYKQRCVTYCRGR

>starPep_43478

WTRCSSSCGRGVSVRSR

>starPep_40170

SPWSQCTASCGGGVQTR

>starPep_40163

SPWDIASVTAGGVQKRS

>starPep_00543

RWCFRVCYRGICYRKCR

>starPep_36015

QRTESIIHRALYYDLIS

>starPep_12021

NGVQPKYKWWKWWKKWW

>starPep_32622

MLQNSAVLLLLVISASA

>starPep_00003

KWCFRVCYRGICYRRCR

>starPep_00145

KWCFRVCYRGICYRKCR

>starPep_27939

KWCRKWQWRGVKFIKCV

>starPep_10849

KLLKLLLKLYKKLLKLL

>starPep_10832

KLALKLALKALKAAKLA

>starPep_26478

KGCALVKVRGLTLKVCK

>starPep_02672

ILGAILPLVSGLLSNKL

>starPep_25171

IIGPVLGLIGKALGGLL

>starPep_05958

IIGPVLGLVGKALGGLL

>starPep_24627

HKCAKIKWRGVHVKYCA

>starPep_01434

GTGLPMSERRKIMLMMR

>starPep_22576

GKEFKRIVWLSKTAKKL

>starPep_22574

GKEFKRIVKWPWWPWRR

>starPep_22573

GKEFKRIVGRIYRLCCR

>starPep_22164

GHRATSDLASTGEESQD

>starPep_02569

GFKRIVQRIKDFLRNLV

>starPep_03963

FVKLKKIANIINSIFKK

>starPep_05425

FSPQMLQDIIEKKTKIL

>starPep_03961

FVDLKKIANIINSIFKK

>starPep_05424

FSPQMLQDIIEAATAIL

>starPep_03965

FVKLKKILNIINSIFKK

>starPep_03964

FVKLKKILNIILSIFKK

>starPep_01306

FLSLIPSLVGGSISAFK

>starPep_03955

FSGGNCRGFRRRCFCTK

>starPep_05405

FLSLIPKLVKKIIKAFK

>starPep_01288

FLGMIPGLIGGLISAFK

>starPep_00270

FLIGMTQGLICLITRKC

>starPep_01723

FLPAALAGIGGILGKLF

>starPep_05363

FLGMIPKLIKKLIKAFK

>starPep_09526

FKRLAKIKVLRLAKIKR

>starPep_03036

FKLFKKIPKFLHLAKKF

>starPep_03917

FKDLKKIANIINSIFKK

>starPep_03922

FKKLKKIANIINSIFKK

>starPep_03022

FFSLIPSLVGGLISAFK

>starPep_19490

FFKLIPKLVKGLISAFK

>starPep_01280

FFSLLPSLIGGLVSAIK

>starPep_19548

FFSLIPKLVKGLISAFK

>starPep_19158

ESLARPCAPGAPAEARL

>starPep_08575

AXQNMEILEXTPLTXVX

>starPep_16327

CDSDSDITWDQLWDLMK

>starPep_41784

VGTDFSGNDDISDVQK

>starPep_03684

VARGWKRKCPLFGKGG

>starPep_04732

RWFKIQMQIRRWKNKK

>starPep_04733

RWFRIQLQIRRWRNRR

>starPep_04731

RWFKIQLQIRRWKNKK

>starPep_04697

RQIKIWFQNRRLKWKK

>starPep_36668

RIFGESVSLRVQDWEW

>starPep_11361

LVPFIGRTLGGLLARF

>starPep_06547

LGGIVSAVKKIVDFLG

>starPep_04347

KWFKIQLQIKKWKNKK

>starPep_06266

KLAKKLAKLAKLAKAL

>starPep_26006

ITMQGIQGQKIRMIMF

>starPep_25660

INEFLERSGIPRQRNQ

>starPep_00772

ILGKLLSTAAGLLSNL

>starPep_05976

ILGKLLKTAAKLLSNL

>starPep_05984

ILGKLLSTWAGLLSNL

>starPep_05978

ILGKLLSTAAGLLSKL

>starPep_05983

ILGKLLSTAWKLLSNL

>starPep_05993

ILKKLLSTAAGLLSNL

>starPep_05985

ILGKLLSWAAGLLSNL

>starPep_05977

ILGKLLSTAAGLLKNL

>starPep_05981

ILGKLLSTAWGLLSKL

>starPep_05982

ILGKLLSTAWGLLSNL

>starPep_05980

ILGKLLSTAAKLLSNL

>starPep_05975

ILGKLLKTAAGLLSNL

>starPep_05979

ILGKLLSTAAKLLSKL

>starPep_25370

ILGKLLSTAWKLLSKL

>starPep_03195

GLVGTLLGHIGKAILG

>starPep_03196

GLVGTLLGHIGKAILS

>starPep_05682

GLFKVIKKVAKVIKKL

>starPep_00203

GLLDIVKKVVGAFGSL

>starPep_03162

GLFKVIKKVASVIGGL

>starPep_05675

GLFDVIKKVASVIKKL

>starPep_00313

GLFDIIKKVASVIGGL

>starPep_02601

GLFDVIAKVASVIKKL

>starPep_09886

GLFDVIKAVASVIGGL

>starPep_05666

GLFAVIKKVAKVIKKL

>starPep_00125

GLFDIIKKVASVVGGL

>starPep_05667

GLFAVIKKVASVIKGL

>starPep_02600

GLFAVIKKVASVIKKL

>starPep_03160

GLFAVIKKVASVIGGL

>starPep_05674

GLFDVIKKVAAVIGGL

>starPep_05664

GLFAVIKKVAAVIKKL

>starPep_22777

GLFAVIHKVASVIGGL

>starPep_00319

GLFDIVKKVVGALGSL

>starPep_22776

GLFAVIHHVASVIGGL

>starPep_22778

GLFAVIKHVASVIGGL

>starPep_05665

GLFAVIKKVAAVIRRL

>starPep_01374

GLFDIVKKVVGTLAGL

>starPep_09888

GLFDVIKKVASVIKGL

>starPep_00036

GLFDVIKKVASVIGGL

>starPep_00970

FLPIVGKLLSGLSGLL

>starPep_09539

FLGAIAQALTSLLGKL

>starPep_09413

FALALKKALKALKKAL

>starPep_09340

FAKIIAKIAKIAKKIL

>starPep_17954

DLWIRETLTSPKSLID

>starPep_17955

DLWIRETLTSPKSLTG

>starPep_16758

CLNLKALLAVAKKILC

>starPep_05058

ASVVNKLTGGVAGLLK

>starPep_44661

YLKNYRIATFKNWPF

>starPep_03704

WFKKIPKFLHLAKKF

>starPep_42935

WDLVVVSAGVAEVGV

>starPep_07937

VYWKKILGKIIKVVK

>starPep_00881

VNWKKILGKIIKVAK

>starPep_07885

VNXKKLLGKLLKVVK

>starPep_04828

VNWKKILAKIIKVVK

>starPep_07882

VNWKKXLGKXIKXVK

>starPep_07877

VNWKKILGKIKKVVK

>starPep_42175

VNWKKVLAKIIKVVK

>starPep_07881

VNWKKVLGKVVKVVK

>starPep_07879

VNWKKLLGKLLKVVK

>starPep_00882

VNWKKVLGKIIKVAK

>starPep_04829

VNWKKILKKIIKVVK

>starPep_07871

VNFKKLLGKLLKVVK

>starPep_07883

VNWRRILGRIIRVVR

>starPep_04830

VNWKKILPKIIKVVK

>starPep_07884

VNWXXILGXIIXVVX

>starPep_13347

VAKKLAKLAKKLLAL

>starPep_40544

SVSGGGHHHHHHGGG

>starPep_12814

RSMRLSFRARGYGFR

>starPep_35207

PTGERLRTCERLSYP

>starPep_34913

PIDERLRTCERLSYP

>starPep_04593

NVWKKILGKIIKVVK

>starPep_07118

NKWKKILGKIIKVVK

>starPep_04405

LLGDFKRIVQRIKDF

>starPep_02730

LKLLKKLLKKLLKLL

>starPep_06348

KNWKKXLKKXIKXVK

>starPep_06346

KNWKKILGKIIKVVK

>starPep_06347

KNWKKILKKIIKVVK

>starPep_10855

KLLRLLKKLLRLLLK

>starPep_06248

KKLLPIVANLLKSLL

>starPep_25665

INGSLDKRVQDCYHG

>starPep_03295

ILPIIGKILSTIFGK

>starPep_25664

INGSLDKRLLPDVET

>starPep_10092

GVGSPYVSRLLGICL

>starPep_05783

GQSQWRDVCRNFMRR

>starPep_03989

GFGSKPLDSFGLNFF

>starPep_03108

GFGSKPIDSFGLSWL

>starPep_20314

FRRPFKWFRRFFKFF

>starPep_20308

FRRFFKWFRRPFKFF

>starPep_20315

FRRPFKWPRRFFKFF

>starPep_20309

FRRFFKWPRRFFKFF

>starPep_20310

FRRFFKWPRRPFKFF

>starPep_20307

FRRFFKWFRRFFKFF

>starPep_19944

FLKDHRISTFKNWPF

>starPep_09361

FAKKLAKLAKKLLAL

>starPep_09362

FAKKLAKLALKLAKL

>starPep_09403

FALAKKALKKAKKAL

>starPep_09364

FAKKLKKLAKLAKKL

>starPep_09344

FAKKLAKKAKLAKKL

>starPep_09331

FAFGKGIGKIGKKGL

>starPep_09332

FAFGKGIGKVGKKLL

>starPep_09337

FAKALKALLKALKAL

>starPep_17443

DDDDDNDKIPDDRDN

>starPep_00911

ANDPQCLYGNVAAKF

>starPep_08248

ADPRNPLEECFRETD

>starPep_08073

YKQCHKKGGKKGSG

>starPep_43831

XFLGTLVNLAKKIL

>starPep_03707

WKKIPKFLHLAKKF

>starPep_03708

WKKIPKFLHLLKKF

>starPep_42725

VVGSPSAQDEASPL

>starPep_41295

TSLDASIIWAMMQN

>starPep_40840

TEENRELVSELKRP

>starPep_40547

SVSRAGSPSGGPFC

>starPep_13094

STRUCTUREGIVEN

>starPep_36941

RKLILKRKRILIKR

>starPep_37281

RLLRLLRRLLRLLR

>starPep_37280

RLLRLLRLRRLLRL

>starPep_35950

QQMNQKDFLSLIVS

>starPep_29516

LSSTCILVLVKSTY

>starPep_02736

LRLKSIVSYAKKVL

>starPep_29073

LLRISLLLIQSWLE

>starPep_28678

LILKRKRKRKRILI

>starPep_00365

LKLKSIVSWAKKVL

>starPep_27628

KRKLILKRILIKRK

>starPep_27129

KLILKRKRKRILIK

>starPep_27184

KLLKLLKKLLKLLK

>starPep_27185

KLLKLLKLKKLLKL

>starPep_26755

KKFFRAWWARRFLK

>starPep_26645

KIKSCYYLPCFVTS

>starPep_26756

KKFFRAWXAPRFLK

>starPep_26754

KKFFRAWWAPRFLK

>starPep_06191

KFLGTLVNLAKKIL

>starPep_10409

INLKILARLAKKIL

>starPep_25650

IMRIKQGQIGQMTI

>starPep_00080

IDWKKLLDAAKQIL

>starPep_24872

HXLGTLVNLAKKIL

>starPep_24550

HFLGXLVNLAKKIL

>starPep_24542

HFLGTLVNLAKKXL

>starPep_24549

HFLGTXVNLAKKIL

>starPep_24547

HFLGTLVXLAKKIL

>starPep_00498

HFLGTLVNLAKKIL

>starPep_24548

HFLGTLXNLAKKIL

>starPep_24540

HFLGTLVNLAKKIX

>starPep_24545

HFLGTLVNXAKKIL

>starPep_24553

HFXGTLVNLAKKIL

>starPep_24551

HFLXTLVNLAKKIL

>starPep_24544

HFLGTLVNLXKKIL

>starPep_01362

GILGKLWEGFKSIV

>starPep_02583

GILGKLWEGVKSIF

>starPep_21830

GFHDHGPCDPPSHK

>starPep_09374

FAKLLAKALKKFAL

>starPep_09345

FAKKLAKKLAKAAL

>starPep_18157

DSSPVSTEQLAPTA

>starPep_13859

YAKLLAKLAKKAL

>starPep_43878

XIIFPXPLPINAI

>starPep_43911

XIXVILPPLPIFG

>starPep_42658

VTCGGGVQKRSRL

>starPep_00158

VRRFPWWWPFLRR

>starPep_13348

VAKLLAKALKKLL

>starPep_13350

VAKLLAKLAKKVL

>starPep_13344

VAKFLAKFLKKAL

>starPep_13349

VAKLLAKLAKKLL

>starPep_40127

SPNITVTLKKFPL

>starPep_38107

RRRYIGRYVRFWK

>starPep_37637

RQVFQVAYIIIKA

>starPep_01565

RLFDKIRQVIRKF

>starPep_29631

LVPLPKIKNSTFT

>starPep_00527

LLPIVGNLLKSLL

>starPep_06541

LFGMALKLLKKVL

>starPep_03381

KWKLFKKIPLKKF

>starPep_06513

KYKKALKKLAKLL

>starPep_02297

KWFRVYRGIYRRR

>starPep_10586

KGVSGHGQHGVHG

>starPep_10502

KAKLAKKALAKLL

>starPep_25841

IRKLKSWKWLRWL

>starPep_25670

INLEACLGRTLMD

>starPep_25631

ILSLRWRWWKWKK

>starPep_25632

ILSLRWWRKWWKK

>starPep_10399

ILSLRWRWKWWKK

>starPep_01457

IFGAIWKGISSLL

>starPep_24709

HNRTPENFPCKNL

>starPep_09883

GLFDIWKKLRWRR

>starPep_00073

GLFDIIKKIAESF

>starPep_00312

GLFDIIKKIAESI

>starPep_09884

GLFDIWKKWRWRR

>starPep_22798

GLFDKWAWWRWRR

>starPep_09882

GLFDIWAWWRWRR

>starPep_09885

GLFDIWKWWRWRR

>starPep_05493

GFGMALXLLXXVL

>starPep_05491

GFGMALKXLKKVL

>starPep_05490

GFGMALKLXKKVL

>starPep_05492

GFGMALRLLRRVL

>starPep_00993

GFGMALKLLKKVL

>starPep_05494

GFGMAXKLLKKVL

>starPep_05503

GFKMALKLLKKVL

>starPep_20557

FXISVSSXVSVIX

>starPep_20556

FXISFSSXFSVIX

>starPep_00061

FVQWFSKFLGRIL

>starPep_20558

FXISWSSXWSVIX

>starPep_09580

FLYIVAKLLSGLL

>starPep_01308

FLSTIWNGIKSLL

>starPep_00435

FLSGIVGMLGKLF

>starPep_00629

FLPLLASLFSRLF

>starPep_00006

FLPLIGRVLSGIL

>starPep_20029

FLPIVGLLKSLLK

>starPep_19952

FLKGIVGMLGKLW

>starPep_19951

FLKGIVGMLGKLL

>starPep_03929

FLKLLKKLAAKLF

>starPep_19947

FLKGIKGMLGKLF

>starPep_02130

FLPFLKSILGKIL

>starPep_19949

FLKGIVGKLGKLF

>starPep_19948

FLKGIKGMLGKLL

>starPep_05375

FLKLLAGLLKNFA

>starPep_19950

FLKGIVGMLGKLF

>starPep_03927

FLGALFKALSKLL

>starPep_09518

FKLAFKLAKKAFL

>starPep_05335

FKRIVQRIKDFLX

>starPep_01286

FLGALWNVAKSVF

>starPep_19826

FLGALFHALSHLL

>starPep_05351

FLGALFKALSHLL

>starPep_05350

FLGALFHALSKLL

>starPep_09397

FAKLLKLAAKKLL

>starPep_09393

FAKLLAKLAKKVL

>starPep_09348

FAKKLAKKLAKLL

>starPep_09378

FAKLLAKFLKKAL

>starPep_09370

FAKLFAKAFKKAL

>starPep_09367

FAKLAKKALAKLL

>starPep_09381

FAKLLAKLAKAKA

>starPep_09375

FAKLLAKALKKFL

>starPep_19317

FASGIAGMAGKLF

>starPep_09373

FAKLLAKALKKAL

>starPep_09386

FAKLLAKLAKKAL

>starPep_09395

FAKLLALALKLKL

>starPep_09396

FAKLLFKALKKAL

>starPep_09377

FAKLLAKALKLKL

>starPep_09394

FAKLLAKLAKLKL

>starPep_09387

FAKLLAKLAKKEL

>starPep_09382

FAKLLAKLAKAKG

>starPep_09385

FAKLLAKLAKKAA

>starPep_09390

FAKLLAKLAKKIL

>starPep_09383

FAKLLAKLAKAKL

>starPep_09392

FAKLLAKLAKKSL

>starPep_09372

FAKLLAKAFKKAL

>starPep_09389

FAKLLAKLAKKGL

>starPep_09339

FAKGVGKVGKKAL

>starPep_09338

FAKFLAKFLKKAL

>starPep_09336

FAKALAKLAKKLL

>starPep_18108

DRSTREPIYMSTI

>starPep_08296

AGVSGHGQHGVHG

>starPep_04942

AFGMALKLLKKVL

>starPep_44821

YRIPIVRRLQRR

>starPep_43875

XHXLLPPVPLFG

>starPep_43904

XIXIILPPLPII

>starPep_43979

XLPFFPPVPIIG

>starPep_13836

XXEXXXFLIILG

>starPep_43649

WYTXXXTWXWXY

>starPep_41781

VGSGGCMFGNGK

>starPep_40291

SRXRTLXTTNGT

>starPep_03618

RRWRIVVIRVRR

>starPep_12734

RRRRRRRRGGCX

>starPep_37471

RPFVEMYSEIPE

>starPep_36313

RCRLAERRQIAK

>starPep_12252

PRFWEYWLRLAE

>starPep_12249

PRFWEYALRLME

>starPep_12253

PRFWEYWLRLME

>starPep_12248

PRFWEAWLRLME

>starPep_12250

PRFWEYWLALME

>starPep_12247

PRAWEYWLRLME

>starPep_12251

PRFWEYWLRAME

>starPep_34463

NOTAVAILABLE

>starPep_11348

LTFEHYWAQLTS

>starPep_29548

LTFSDWWKLLAE

>starPep_11344

LTAEHYAAQATS

>starPep_11013

KWKLAKKALALL

>starPep_06308

KMWSKILGHLIR

>starPep_06294

KLLLKLKLKLLK

>starPep_26794

KKKIIIIIIKKK

>starPep_26172

KAFDITYVRLKF

>starPep_26267

KCGHKHQCAVHN

>starPep_03297

ILPIRSLIKKLL

>starPep_25671

INLEACLKRGRT

>starPep_02266

ILRWPWWPWRRK

>starPep_05770

GMWSKILGHLIK

>starPep_05773

GMWSKILKHLIR

>starPep_05774

GMWSKLLGHLLR

>starPep_05769

GMWKKILGKLIR

>starPep_05772

GMWSKILGKLIR

>starPep_05768

GMWKKILGHLIR

>starPep_05771

GMWSKILGHLKR

>starPep_01421

GMWSKILGHLIR

>starPep_05657

GKWMSLLKKILK

>starPep_05656

GKWMSLLKHWLK

>starPep_05651

GKWKSLLKHILK

>starPep_05654

GKWMSFLKHILK

>starPep_05655

GKWMSLLKHIWK

>starPep_05660

GKWSKILGHLIR

>starPep_01373

GKWMSLLKHILK

>starPep_05661

GKWSKILGKLIR

>starPep_05647

GKWKKILGKLIR

>starPep_05653

GKWMKLLKHILK

>starPep_05658

GKWMSLWKHILK

>starPep_05659

GKWMTLLKHILK

>starPep_05646

GKWKKILGHLIR

>starPep_05652

GKWLSLLKHILK

>starPep_05627

GKFMSLLKHILK

>starPep_21144

GDVIDTDRDIDR

>starPep_03057

FLPLKKLRFGLL

>starPep_09399

FAKLWAKLAKKL

>starPep_09414

FALALKLAKKAL

>starPep_09420

FALLKALLKKAL

>starPep_09363

FAKKLKKLAKKL

>starPep_09365

FAKKLLAKALKL

>starPep_19185

ETFADWWKLLAE

>starPep_19146

ESFSDWWKLLAE

>starPep_19188

ETFSDWWKLLAE

>starPep_09342

FAKKALKALKKL

>starPep_17653

DFKLFAVYIKYR

>starPep_17652

DFKLFAVTIKYR

>starPep_16860

CQNHHAKHGKVC

>starPep_01648

AKRHHGYKRKFH

>starPep_44905

YTMNPRKLFDY

>starPep_44781

YPYDVPDYASL

>starPep_43857

XGLXGKLXGIX

>starPep_43938

XKLXGGLXGIX

>starPep_44085

XRLXGGLXGIX

>starPep_43984

XLPPPPLPFFF

>starPep_43858

XGLXKKLXGIX

>starPep_43895

XISQIISTAXI

>starPep_43854

XGLXGGLXKIX

>starPep_43851

XGLXGGLLGIX

>starPep_43855

XGLXGGLXRIX

>starPep_43852

XGLXGGLXGIX

>starPep_43845

XGLLGGLXGIX

>starPep_13691

WLWKAIWKLLT

>starPep_13690

WLWKAIWKLLK

>starPep_00875

TESYFVFSVGM

>starPep_40891

TGASSEEEDPF

>starPep_40889

TGALVEEEDPF

>starPep_40402

SSTSPHRPRFS

>starPep_39915

SLLSLIRLLIT

>starPep_39908

SLLPLIRKLIT

>starPep_00232

SLLSLIRKLIT

>starPep_39914

SLLSLIRKLLT

>starPep_39913

SLLSLIRKLIW

>starPep_39537

SFLSLIRKLIT

>starPep_03612

RRLFRRILRWL

>starPep_03613

RRLFRRILRYL

>starPep_38021

RRRLLLLLRRR

>starPep_38011

RRRIIIIIRRR

>starPep_37934

RRLFRRILRRL

>starPep_37292

RLLSLIRKLIT

>starPep_12182

PLLQATLGGGS

>starPep_30563

MEPECNLNCTD

>starPep_04394

LKLFKKILKFL

>starPep_04395

LKLFKKILKYL

>starPep_11180

LKKLAKLALAF

>starPep_03404

KWLRRVWRWWR

>starPep_06509

KWXWKXVKXAK

>starPep_06508

KWXWKXAKXAK

>starPep_27636

KRKSGSGSKRK

>starPep_27587

KRKILILILIL

>starPep_27580

KRKILILIGSG

>starPep_03351

KRLRRVWRRWR

>starPep_06353

KPLLKKLLKKL

>starPep_26871

KKLFKKILKHL

>starPep_26872

KKLFKKILKIL

>starPep_26865

KKLFKKGLKFL

>starPep_26869

KKLFKKILKEL

>starPep_26875

KKLFKKILKRL

>starPep_26870

KKLFKKILKGL

>starPep_26873

KKLFKKILKLL

>starPep_26876

KKLFKKILKTL

>starPep_04275

KKLFKKILKFL

>starPep_26867

KKLFKKILKAL

>starPep_26844

KKKLLLLLKKK

>starPep_26874

KKLFKKILKQL

>starPep_01870

KKLFKKILKYL

>starPep_26868

KKLFKKILKDL

>starPep_26854

KKKVVVVVKKK

>starPep_26795

KKKIIIIIKKK

>starPep_06210

KISKRILTGKK

>starPep_26791

KKKFFFFFKKK

>starPep_06147

KAXWKXVKXAK

>starPep_25419

ILILILILKRK

>starPep_10272

IELLQARGGCX

>starPep_24661

HKLINTEGHHS

>starPep_24572

HGSTTLRDITV

>starPep_24115

GVDITVIRPNH

>starPep_23905

GSGILILIKRK

>starPep_02544

FLWWLFKWAWK

>starPep_19930

FLGWLFKVASK

>starPep_02536

FLKWLFKWAKK

>starPep_02532

FLGWLFKWAKK

>starPep_02533

FLGWLFKWAWK

>starPep_19841

FLGALFKWASK

>starPep_09415

FALALKLAKKL

>starPep_16341

CELDENNTPMC

>starPep_00408

AVPDVAFNAYG

>starPep_45004

YTYGLCTSSR

>starPep_44137

XVPVFPPLFI

>starPep_43856

XGLXGKLXGI

>starPep_44138

XVPVFPPLPI

>starPep_43937

XKLXGGLXGI

>starPep_08015

XGLXGGLXGI

>starPep_02914

VXLFPVXLFP

>starPep_42545

VRSCMFGNGK

>starPep_40890

TGALVQQQDP

>starPep_01190

SNDIYFNFQR

>starPep_12742

RRRRRWCMNW

>starPep_38573

RTXCXFLRXC

>starPep_38552

RTRCRFLRRC

>starPep_38555

RTRCXFLRXC

>starPep_12730

RRRRRNWMWC

>starPep_30224

MARRASVGTD

>starPep_28995

LLKKKFLKKQ

>starPep_28994

LLKKKFKLKQ

>starPep_29021

LLLKKFKKKQ

>starPep_29022

LLLKKFKKLQ

>starPep_29005

LLKLKFKKKQ

>starPep_29006

LLKLKFKKLQ

>starPep_29027

LLLLKFKKLQ

>starPep_28993

LLKKKFKKLQ

>starPep_28997

LLKKLFKKKQ

>starPep_28843

LKLLKFKKLQ

>starPep_28833

LKLKKFLKKQ

>starPep_28794

LKKLLFKKKQ

>starPep_28831

LKLKKFKKLQ

>starPep_28784

LKKKLFKKLQ

>starPep_28785

LKKKLFKLKQ

>starPep_11162

LIAHNQVRQV

>starPep_28842

LKLLKFKKKQ

>starPep_28792

LKKLKFLKKQ

>starPep_28832

LKLKKFKLKQ

>starPep_28840

LKLKLFKKKQ

>starPep_28777

LKKKKFLKLQ

>starPep_28774

LKKKKFKKLQ

>starPep_28775

LKKKKFKLLQ

>starPep_28790

LKKLKFKKLQ

>starPep_28791

LKKLKFKLKQ

>starPep_28786

LKKKLFLKKQ

>starPep_28778

LKKKKFLLKQ

>starPep_00220

KTCENLADTY

>starPep_00808

KTCENLADDY

>starPep_01125

KQTENLADTY

>starPep_27194

KLLLKFKKKQ

>starPep_27151

KLKKLFKKLQ

>starPep_27140

KLKKKFKKLQ

>starPep_27144

KLKKKFLLKQ

>starPep_27179

KLLKKFKLKQ

>starPep_02281

KKVVFKVKFK

>starPep_27163

KLKLKFLKKQ

>starPep_27195

KLLLKFKKLQ

>starPep_06281

KLKLKFKLKQ

>starPep_27162

KLKLKFKKLQ

>starPep_27143

KLKKKFLKLQ

>starPep_27164

KLKLLFKKKQ

>starPep_27141

KLKKKFKLLQ

>starPep_27180

KLLKKFLKKQ

>starPep_06255

KKVVFWVKFK

>starPep_27152

KLKKLFKLKQ

>starPep_27153

KLKKLFLKKQ

>starPep_27183

KLLKLFKKKQ

>starPep_27178

KLLKKFKKLQ

>starPep_26822

KKKKLFLKLQ

>starPep_26940

KKLLKFLKKQ

>starPep_26804

KKKKKFLLLQ

>starPep_26939

KKLLKFKLKQ

>starPep_26823

KKKKLFLLKQ

>starPep_26923

KKLKKFLKLQ

>starPep_26943

KKLLLFKKKQ

>starPep_26840

KKKLLFKKLQ

>starPep_26821

KKKKLFKLLQ

>starPep_26938

KKLLKFKKLQ

>starPep_26842

KKKLLFLKKQ

>starPep_26837

KKKLKFLKLQ

>starPep_26838

KKKLKFLLKQ

>starPep_26920

KKLKKFKKLQ

>starPep_26835

KKKLKFKKLQ

>starPep_26803

KKKKKFKKLQ

>starPep_26933

KKLKLFLKKQ

>starPep_26836

KKKLKFKLLQ

>starPep_26932

KKLKLFKLKQ

>starPep_26921

KKLKKFKLLQ

>starPep_26841

KKKLLFKLKQ

>starPep_26924

KKLKKFLLKQ

>starPep_26931

KKLKLFKKLQ

>starPep_24926

IARALFEKKV

>starPep_04133

GXRKXHKXWA

>starPep_05524

GFRKFHKFWA

>starPep_20237

FPWWNQYVKL

>starPep_20194

FPFFNQYVXL

>starPep_20234

FPWFNQYVKL

>starPep_20193

FPFFNQYVKL

>starPep_20238

FPWWNQYVXL

>starPep_20235

FPWFNQYVXL

>starPep_05342

FKVKFKVKVK

>starPep_09418

FALKALKKAL

>starPep_09356

FAKKLAKKLL

>starPep_09406

FALALKAKKL

>starPep_09379

FAKLLAKKLL

>starPep_19208

EVHHQKLVFF

>starPep_09343

FAKKLAKALL

>starPep_09167

DYPKLTFTTS

>starPep_00929

DNGEAGRAAR

>starPep_17605

DETXTDEEFN

>starPep_00927

DDFLCAGGCL

>starPep_17101

CTTHWGFTLC

>starPep_43413

WRRRRRRRR

>starPep_00553

SVAGRAQGM

>starPep_38774

RWKKWWRWL

>starPep_38787

RWKRWWRWI

>starPep_38398

RRWXRRWWR

>starPep_37350

RLWKRWWIR

>starPep_07498

RLLLRIGRR

>starPep_04689

RLRLRIGRR

>starPep_36975

RKRWWWWFR

>starPep_07517

RLYLRIGRR

>starPep_07428

RGSALTHLP

>starPep_36787

RIWKIWWKR

>starPep_01956

PGMGIYLPM

>starPep_28141

KWWKIWRWR

>starPep_28151

KWWRWRRWW

>starPep_11011

KWKKLAKKW

>starPep_27572

KRKGSGKRK

>starPep_27732

KSGXKHKKK

>starPep_27114

KLGVPLARK

>starPep_27117

KLGVPLKRK

>starPep_27112

KLGVALKRK

>starPep_26982

KKRWWWWWR

>starPep_27051

KKWXKKWWK

>starPep_27052

KKWXKKWXK

>starPep_27115

KLGVPLKAK

>starPep_27116

KLGVPLKRA

>starPep_27047

KKWWKKXWK

>starPep_27046

KKWWKKWWK

>starPep_10592

KIFGSLAFL

>starPep_10553

KFKKLAKKW

>starPep_10552

KFKKLAKKF

>starPep_26218

KAQXQKQAW

>starPep_26094

IWKRWWWKR

>starPep_25337

IKWKRWWWR

>starPep_09368

FAKLAKKLL

>starPep_08905

CSSRTMHHC

>starPep_08692

CHHNATHAC

>starPep_08693

CHHNLAHAC

>starPep_08686

CHANLTHAC

>starPep_08695

CHHNLTHAC

>starPep_08694

CHHNLTAAC

>starPep_08691

CHHALTHAC

>starPep_08582

CAHNLTHAC

>starPep_15904

AWXLFDDGV

>starPep_02461

ALYLAIRRR

>starPep_14910

ALGVPLKRK

>starPep_45099

YYYYRRRR

>starPep_08001

WWWLRKIW

>starPep_43592

WWWLKRIW

>starPep_43830

XFFLSRIF

>starPep_43594

WWWLRRIW

>starPep_43591

WWWLKKIW

>starPep_38080

RRRRWWWW

>starPep_03603

RLGDGCTR

>starPep_29032

LLLLRRRR

>starPep_27466

KQRWLWLW

>starPep_27413

KPPPWVPV

>starPep_25176

IIIIRRRR

>starPep_20554

FXFLRRIF

>starPep_19674

FIRSLFFF

>starPep_19440

FFFFRRRR

>starPep_01275

FFFLSRIF

>starPep_19447

FFFLRRIF

>starPep_15908

AXEGSNXX

>starPep_14028

AAAARRRR

>starPep_44737

YPLPFIP

>starPep_44107

XRWRWRW

>starPep_43833

XFXPXLV

>starPep_42958

WEXWTIW

>starPep_38853

RWRWRWX

>starPep_35150

PRPRPRP

>starPep_28687

LIPFPFP

>starPep_04404

LLDVLLE

>starPep_28706

LIXFXPX

>starPep_27571

KRKGKRK

>starPep_26155

IYQAATX

>starPep_09658

FXYWKXT

>starPep_20401

FTVAXFI

>starPep_05215

ELLVDLL

>starPep_13906

YGGFMX

>starPep_13810

XLLVNX

>starPep_43859

XGNILL

>starPep_43611

WXLVNX

>starPep_04870

WRWFIH

>starPep_43205

WLLXNX

>starPep_13678

WLLVXX

>starPep_13672

WLLINX

>starPep_43254

WLXXNG

>starPep_13669

WLLANX

>starPep_13673

WLLISX

>starPep_13670

WLLIIX

>starPep_03710

WLLVNG

>starPep_43203

WLLVNX

>starPep_42997

WGNILL

>starPep_43236

WLVXNG

>starPep_43067

WILVNX

>starPep_43198

WLIVNX

>starPep_13666

WLAVNX

>starPep_43204

WLLXNG

>starPep_13677

WLLVAX

>starPep_13674

WLLIXX

>starPep_43252

WLXVNX

>starPep_13606

WALVNX

>starPep_03703

WAIVLL

>starPep_42915

WAXVLL

>starPep_04808

TWWRWW

>starPep_13266

TPFVXV

>starPep_12890

RYLGYL

>starPep_01955

PGLGFY

>starPep_34690

NVLLWG

>starPep_34693

NVLLWX

>starPep_34033

NILLWG

>starPep_34026

NIILWG

>starPep_29091

LLWVNX

>starPep_04363

KWRWIW

>starPep_03403

KWKWKW

>starPep_04364

KWWWRW

>starPep_27623

KRKKRK

>starPep_09421

FALLKL

>starPep_08378

ALLVNX

>starPep_14008

YXGFM

>starPep_13961

YPFPG

>starPep_44083

XRIRL

>starPep_13605

WALAL

>starPep_12224

PPPEE

>starPep_11969

MTLTG

>starPep_10503

KAKLF

>starPep_20603

FYPFG

>starPep_05152

DEDDD

>starPep_00908

AMVGT

>starPep_00250

ACSAG

>starPep_13990

YRXG

>starPep_13996

YSXG

>starPep_44722

YPFX

>starPep_43978

XLLL

>starPep_23957

GSPE

>starPep_09620

FSAR

>starPep_09289

ERRP

>starPep_05214

ELLL

>starPep_09033

DEVD

>starPep_13995

YSX

>starPep_43187

WKX

>starPep_13451

VKX

>starPep_12176

PKX

>starPep_08357

AKX

>starPep_14013

ZV

>starPep_13602

VZ
