# Supplementary material for: Automatic construction of molecular similarity networks for visual graph mining in chemical space of bioactive peptides: an unsupervised learning approach
Source: Sci Rep. 2020 Oct 22;10:18074. doi: 10.1038/s41598-020-75029-1 (PMC7583304; doi:10.1038/s41598-020-75029-1)
Supplement: Supplementary file 10 — Supplementary Information 9. [file 41598_2020_75029_MOESM10_ESM.zip › SI4_1-4_Anticancer_peptides/SI4-3_Anticancer_NR98_LeadingComm.fasta.docx]

**Supporting Information:**

Automatic construction of similarity networks for visual graph mining in chemical space of bioactive peptides: an unsupervised learning approach

Longendri Aguilera-Mendoza, Yovani Marrero-Ponce*, César R. García-Jacas, Edgar Chavez, Jesus A. Beltran, Hugo A. Guillen-Ramirez, Carlos A. Brizuela*.

Corresponding authors *: Y. Marrero-Ponce: ymarrero@usfq.edu.ec or ymarrero77@yahoo.es; Carlos A. Brizuela: cbrizuel@cicese.edu.mx

**Contents**: Leading communities (FASTA format) detected in the chemical space of anticancer peptides.

Contenido

[Community 1 1](#_Toc46225020)

[Community 2 11](#_Toc46225021)

[Community 3 18](#_Toc46225022)

[Community 4 23](#_Toc46225023)

[Community 5 26](#_Toc46225024)

[Community 6 29](#_Toc46225025)

[Community 7 32](#_Toc46225026)

[Community 8 33](#_Toc46225027)

# Community 1

>starPep_05013

ALWKTLLKKVLKAAAKAALNAVLVGANA

>starPep_02206

GLFDIVKKVVGTIAGL

>starPep_00182

GFFALIPKIISSPLFKTLLSAVGSALSSSGGQE

>starPep_01650

ALWKNMLKGIGKLAGQAALGAVKTLVGAE

>starPep_08393

ALWKSLLKNVGKAAGKAALNAVTDMVNQ

>starPep_14993

ALWKEVLKNAGKAALNEINNLVQ

>starPep_01646

AGYLLGKINLKALAALAKKIL

>starPep_00316

GLFDIVKKIAGHIVSSI

>starPep_00315

GLFDIVKKIAGHIASSI

>starPep_00314

GLFDIVKKIAGHIAGSI

>starPep_07415

RGDLLRHVVKILSKYL

>starPep_00311

GLFDIAKKVIGVIGSL

>starPep_01847

ILPILSLIGGLLGK

>starPep_00010

ALWKTMLKKLGTMALHAGKAALGAAADTISQGTQ

>starPep_00484

GLWSKIKEVGKEAAKAAAKAAGKAALGAVSEAV

>starPep_00483

GLWSKIKEAAKAAGKAALNAVTGLVNQGDQPS

>starPep_23443

GMWSKIKETAMAAAKEAAKAAGKTISDMIKQ

>starPep_08395

ALWKTMLKKLGTVALHAGKAALGAVADTISQ

>starPep_01142

LKIPGFVKDTLKKVAKGIFSAVAGAMTPS

>starPep_11149

LGQSAASAHHAYIVLAIENSFMTASKKK

>starPep_01109

IKIPAFVKDTLKKVAKGVISAVAGALTQ

>starPep_00098

ALWKDILKNVGKAAGKAVLNTVTDMVNQ

>starPep_08391

ALWKDLLKNVGKAAGKAVLNKVTDMVNQ

>starPep_03758

ALWDTLLKKVLKAAAKAALDAVLVGANA

>starPep_03761

ALWKTLLKKVLKAAAKAALKAVLVGANA

>starPep_00855

SIGAKILGGVKTFFKGALKELASTYLQ

>starPep_00675

GIGTKILGGVKTALKGALKELASTYAN

>starPep_01769

GIGGALLSAGKSALKGLAKGLAEHFAN

>starPep_00301

GIGGVLLSAGKAALKGLAKVLAEKYAN

>starPep_00674

GIGGKILSGLKTALKGAAKELASTYLH

>starPep_03125

GIFPIFAKLLGKVIKVASSLISKGRTE

>starPep_22216

GIFPIFAKLLGKVIKVASSLISKGRTK

>starPep_24256

GVWGIAKIAGKVLGNILPHVFSSNQS

>starPep_09823

GIGVLLSAGKAALKGLAKVLAEKYAN

>starPep_00569

AGWGSIFKHIFKAGKFIHGAIQAHND

>starPep_00545

RWGKWFKKATHVGKHVGKAALTAYL

>starPep_00494

GWGSIFKHGRHAAKHIGHAAVNHYL

>starPep_00008

GWGSFFKKAAHVGKHVGKAALTHYL

>starPep_02849

RKGWFKAMKSIAKFIAKEKLKEHL

>starPep_26099

IWLTALKFLGKNLGKLAKQQLAKL

>starPep_00323

GLFKVLGSVAKHLLPHVAPVIAEK

>starPep_00419

FLGALIKGAIHGGRFIHGMIQNHH

>starPep_02606

GLFVGLAKVAAHNNPAIAEHFQA

>starPep_00321

GLFGVLAKVASHVVPAIAEHFQA

>starPep_09816

GIGKFLHSAKKWGKAFVGQIMNC

>starPep_00001

GIGKFLHSAKKFGKAFVGEIMNS

>starPep_01013

GIGKFLHAAKKFAKAFVAEIMNS

>starPep_02191

GIGKFLHSAKKFAKAFVAEIMNS

>starPep_02121

FIHHIIGGLFSVGKHIHSLIHGH

>starPep_00570

AIGSILGALAKGLPTLISWIKNR

>starPep_08303

AIGKFLHSAKKFGKAFVGEIMNS

>starPep_14990

ALWKDILKNLLKAALNEINQIVQ

>starPep_14994

ALWKKILKNAGKAALNKINQIVQ

>starPep_00577

ALWKDILKNAGKAALNEINQIVQ

>starPep_32958

MQFITDLIKKAVDVFKGLFGNK

>starPep_00005

FFHHIFRGIVHVGKTIHRLVTG

>starPep_09335

FAKAIAKIAFGKGIGKVGKKLL

>starPep_41197

TQQAFQKFLAAVTSALGKQYH

>starPep_02568

GFIFHIIKGLFHAGKMIHGLV

>starPep_02535

FLHHIVGLIHHGLSLFGDRAD

>starPep_04575

NFAEIFAAVNKLIKQGVVKG

>starPep_02584

GILSKLGKALKKAAKHAAKA

>starPep_20670

GANLAKKFYTYINKFINYAW

>starPep_05498

GFKDLLKGAAKALVKAVLF

>starPep_05499

GFKDLLKGAAKALVKTVKF

>starPep_05497

GFKDLLKGAAKALKKTVLF

>starPep_05501

GFKDLLKKAAKALVKTVLF

>starPep_00185

GFKDLLKGAAKALVKTVLF

>starPep_05500

GFKDLLKGAKKALVKTVLF

>starPep_03114

GFKKLLKGAAKALVKTVLF

>starPep_20132

FLSMIPHIVSGVAALAKHL

>starPep_00640

FLSLIPHIVSGVASIAKHF

>starPep_20127

FLSLIPHIVSGVASLAKHF

>starPep_20129

FLSLIPKIAGGIAALAKHL

>starPep_09578

FLSLIPKIATGIAALAKHL

>starPep_03040

FLFSLIPSAIAGLVSAIRN

>starPep_03041

FLFSLIPSVIAGLVSAIRN

>starPep_19552

FFSMIPKIAGGIASLVKNL

>starPep_00524

LLGMIPLAISAISALSKL

>starPep_00115

GFVDFLKKVAGTIANVVT

>starPep_03083

FPFSLIPHAIGGLISAIK

>starPep_02527

FLFSLIPKAIGGLISAFK

>starPep_01283

FLFSLIPHAIGGLISAFK

>starPep_02672

ILGAILPLVSGLLSNKL

>starPep_25171

IIGPVLGLIGKALGGLL

>starPep_01306

FLSLIPSLVGGSISAFK

>starPep_01288

FLGMIPGLIGGLISAFK

>starPep_01723

FLPAALAGIGGILGKLF

>starPep_03022

FFSLIPSLVGGLISAFK

>starPep_01280

FFSLLPSLIGGLVSAIK

>starPep_19548

FFSLIPKLVKGLISAFK

>starPep_06547

LGGIVSAVKKIVDFLG

>starPep_00772

ILGKLLSTAAGLLSNL

>starPep_05976

ILGKLLKTAAKLLSNL

>starPep_05984

ILGKLLSTWAGLLSNL

>starPep_05978

ILGKLLSTAAGLLSKL

>starPep_05983

ILGKLLSTAWKLLSNL

>starPep_05993

ILKKLLSTAAGLLSNL

>starPep_05985

ILGKLLSWAAGLLSNL

>starPep_05977

ILGKLLSTAAGLLKNL

>starPep_05981

ILGKLLSTAWGLLSKL

>starPep_05982

ILGKLLSTAWGLLSNL

>starPep_05980

ILGKLLSTAAKLLSNL

>starPep_05975

ILGKLLKTAAGLLSNL

>starPep_05979

ILGKLLSTAAKLLSKL

>starPep_25370

ILGKLLSTAWKLLSKL

>starPep_03195

GLVGTLLGHIGKAILG

>starPep_03196

GLVGTLLGHIGKAILS

>starPep_00203

GLLDIVKKVVGAFGSL

>starPep_03162

GLFKVIKKVASVIGGL

>starPep_05675

GLFDVIKKVASVIKKL

>starPep_00313

GLFDIIKKVASVIGGL

>starPep_02601

GLFDVIAKVASVIKKL

>starPep_09886

GLFDVIKAVASVIGGL

>starPep_05666

GLFAVIKKVAKVIKKL

>starPep_00125

GLFDIIKKVASVVGGL

>starPep_05667

GLFAVIKKVASVIKGL

>starPep_02600

GLFAVIKKVASVIKKL

>starPep_03160

GLFAVIKKVASVIGGL

>starPep_05674

GLFDVIKKVAAVIGGL

>starPep_05664

GLFAVIKKVAAVIKKL

>starPep_22777

GLFAVIHKVASVIGGL

>starPep_00319

GLFDIVKKVVGALGSL

>starPep_22776

GLFAVIHHVASVIGGL

>starPep_22778

GLFAVIKHVASVIGGL

>starPep_05665

GLFAVIKKVAAVIRRL

>starPep_01374

GLFDIVKKVVGTLAGL

>starPep_09888

GLFDVIKKVASVIKGL

>starPep_00036

GLFDVIKKVASVIGGL

>starPep_00970

FLPIVGKLLSGLSGLL

>starPep_09539

FLGAIAQALTSLLGKL

>starPep_05058

ASVVNKLTGGVAGLLK

>starPep_06191

KFLGTLVNLAKKIL

>starPep_00498

HFLGTLVNLAKKIL

>starPep_01457

IFGAIWKGISSLL

>starPep_00073

GLFDIIKKIAESF

>starPep_00312

GLFDIIKKIAESI

>starPep_09580

FLYIVAKLLSGLL

>starPep_01308

FLSTIWNGIKSLL

>starPep_00435

FLSGIVGMLGKLF

>starPep_19952

FLKGIVGMLGKLW

>starPep_19951

FLKGIVGMLGKLL

>starPep_19947

FLKGIKGMLGKLF

>starPep_19949

FLKGIVGKLGKLF

>starPep_19948

FLKGIKGMLGKLL

>starPep_05375

FLKLLAGLLKNFA

>starPep_19950

FLKGIVGMLGKLF

>starPep_03927

FLGALFKALSKLL

>starPep_01286

FLGALWNVAKSVF

>starPep_19826

FLGALFHALSHLL

>starPep_05351

FLGALFKALSHLL

>starPep_05350

FLGALFHALSKLL

# Community 2

>starPep_06208

KILRGVSKKIMRTFLRRISKDILTGKK

>starPep_26684

KISKKIMRTFLRRISKDILTGKK

>starPep_03498

MNFNKLFVFVALVLAVCIGQSEAGWLKKIGKKIERVGQHTRDATIQTIGVAQQAANVAAT

LKG

>starPep_00663

GFSSIFRGVAKFASKGLGKDLAKLGVDLVACKISKQC

>starPep_10598

KILRGVAKKIMRTFLRRISKKILTGKK

>starPep_26658

KILRGVSKKIMRRILTGKK

>starPep_02776

MPRWRLFRRIDRVGKQIKQGILRAGPAIALVGDARAVG

>starPep_00791

ISRLAGLLRKGGEKIGEKLKKIGQKIKNFFQKLVPQPE

>starPep_00018

KWKLFKKIEKVGQNIRDGIIKAGPAVAVVGQATQIAK

>starPep_21926

GFSSIFRGVAKFASKGLGKDLAKLGVDLVASKISKQS

>starPep_18007

DPFFKVPVNKLAAAVSNFGYDLYRVRSSTSPTTN

>starPep_08109

YRWYGYTPQNVIGGGKLLLKLLKKLLKLLKKK

>starPep_25343

IKYLLVKLQGASQKTITLMLRRNNLYVMGYS

>starPep_22415

GIMDTVKNAAKNLAGQLLDKLKCKITAC

>starPep_00126

GLFGKLIKKFGRKAISYAVKKARGKH

>starPep_41038

TLPFAYCNIHQVCHYAQRNDRSYWL

>starPep_00759

GWKKWLRKGAKHLGQAAIKGLAS

>starPep_01133

KWKLFKKIGIGAVLKVLTTG

>starPep_16349

CETWRTETTGATGQASSLLSGRLLEQKAASCHNSYIVLCIENSFMTSFSK

>starPep_18164

DTAVTGLASPLSTGKILDQKAYSCANRLIVLCIENSFMTDARK

>starPep_26158

IYSFDGRDIMTDPSWPQKVIWHGSSPHGVRLVDNYCEAWRTA

>starPep_21918

GFRKRFNKLVKKVKHTIKETANVSKDVAIVAGSGVAVGAAM

>starPep_04528

MPKWKVFKKIEKVGRNIRNGIVKAGPAIAVLGEAKALG

>starPep_11260

LPKWKVFKKIEKVGRNIRNGIVKAGPAIAVLGEAKALG

>starPep_38769

RWKIFKKIERVGQNVRDGIIKAGKAIQVLGTAKALGK

>starPep_38770

RWKIFKKIERVGQNVRDGIIKAGPAIQVLGTAKALGK

>starPep_02002

RWKIFKKIEKMGRNIRDGIVKAGPAIEVLGSAKAIGK

>starPep_03179

GLLSVFKGVLKTAGKNVAKNVAGSLLDQLKCKISGGC

>starPep_21927

GFSSIFRGVAKFASKGLGKKLAKLGVKLVACKISKQC

>starPep_07120

NLVSALIEGRKYLKNVLKKLNRLKEKNKAKNSKENN

>starPep_22064

GGLKKLGKKLEGAGKRVFNAAEKALPVVAGAKALRK

>starPep_00546

RWKIFKKIEKVGQNIRDGIVKAGPAVAVVGQAATI

>starPep_00361

KWKVFKKIEKMGRNIRNGIVKAGPAIAVLGEAKAL

>starPep_00146

KWKIFKKIEKVGRNIRNGIIKAGPAVAVLGEAKAL

>starPep_28062

KWKVFKKIEKMGRNIRNGIVKAGPKWKVFKKIEK

>starPep_01059

GLLRKGGEKIGEKLKKIGQKIKNFFQKLVPQPEQ

>starPep_34474

NPEKALEKLIAIQKAIKGMLNGWFTGVGFRRKR

>starPep_00320

GLFDVVKGVLKGVGKNVAGSLLEQLKCKLSGGC

>starPep_18008

DPFFKVPVNKLAAVSNFGYDLYRVRSSMSPTTN

>starPep_08069

YHWYGYTPQNVIGGGKLLLKLLKKLLKLLKKK

>starPep_00089

SWLSKTAKKLENSAKKRISEGIAIAIQGGPR

>starPep_27320

KNECLWTDMLSNFGYPGYQSKHYACIRQKG

>starPep_03315

KFFKRLLKSVRRAVKKFRKKPRLIGLSTLL

>starPep_00795

KFFRKLKKSVKKRAKEFFKKPRVIGVSIPF

>starPep_03423

LKLSPKTKDTLKKVLKGAIKGAIAIASMA

>starPep_25294

IKLSKETKKNLKKVLKGAIKGAIAVAKMV

>starPep_01112

IKLSPETKDNLKKVLKGAIKGAIAVAKMV

>starPep_25297

IKLSPKTKDNLKKVLKGAIKGAIAVAKMV

>starPep_25236

IKIPSFFRNILKKVGKKAVSLIAGALKQS

>starPep_25295

IKLSKKTKDNLKKVLKGAIKGAIAVAKMV

>starPep_25298

IKLSPKTKKNLKKVLKGAIKGAIAVAKMV

>starPep_25293

IKLSKETKDNLKKVLKGAIKGAIAVAKMV

>starPep_25296

IKLSPETKKNLKKVLKGAIKGAIAVAKMV

>starPep_03287

IKIPSFFRNILKKVGKEAVSLIAGALKQS

>starPep_01337

GFMDTAKNVAKNVAVTLIDKLRCKVTGGC

>starPep_07864

VLLVTLTRLHQRGVIYRKWRHFSGRKYR

>starPep_01110

IKIPAVVKDTLKKVAKGVLSAVAGALTQ

>starPep_00720

GLMDTIKGVAKTVAASWLDKLKCKITGC

>starPep_05614

GIMDTIKGAAKDLAGQLLDKLKCKITKC

>starPep_09835

GIMDTVKNAAKNLAGQLLDKLKCSITAC

>starPep_11918

MRKWFHNVLSSGQLLADKWPAWDYNRK

>starPep_10596

KILRGVAKKILRTFLRRISKDILTGKK

>starPep_06206

KILRGVAKKIMRTFLRRISKDILTGKK

>starPep_24842

HTHQDFQPVLHLVALNTPLSGGMRGIR

>starPep_00807

KSSAYSLQMGATAIKQVKKLFKKWGW

>starPep_25166

IIGAVLKVLTTGLPALISWIKRKRQQ

>starPep_00205

GLLQTIKEKLESLESLAKGIVSGIQA

>starPep_05678

GLFGKLIKKKGRKAISYAVKKARGKH

>starPep_09889

GLFGKLIKKFARKAISYAVKKARGKH

>starPep_05679

GLFGKLQKKFGRKAISYAVKKARGKH

>starPep_05677

GLFGKLIKKFLRKAISYAVKKARGKH

>starPep_22239

GIGAVLKVLTTGLPALKSWIKRKRQQ

>starPep_00000

GIGAVLKVLTTGLPALISWIKRKRQQ

>starPep_00657

GFLGILFHGVHHGRKKALHMNSERRS

>starPep_03342

KLKNFAKGVAQSLLNKASCKLSGQC

>starPep_06287

KLKNFAIGVAQSLLNKASCKLSGQC

>starPep_10235

HSHRDFQPVLHLVALNSPLSGGMRG

>starPep_05855

GWRKWIKKATHVGKHIGKAALDAYI

>starPep_01097

GWKKWFNRAKKVGKTVGGLAVDHYL

>starPep_05849

GWKDWFRKAKKVGKTVGGLALNHYL

>starPep_01098

GWKSVFRKAKKVGKTVGGLALDHYL

>starPep_05697

GLKKWFKKAVHVGKKVGKVALNAYL

>starPep_05620

GIRKWFKKAAHVGKKVGKVALNAYL

>starPep_05619

GIRKWFKKAAHVGKEVGKVALNACL

>starPep_05460

GCKKWFKKAAHVGKNVGKVALNAYL

>starPep_00051

FFGWLIKGAIHAGKAIHGLIHRRRH

>starPep_29425

LRSRGELVAKFLAGEQSPEDYVAE

>starPep_01265

DSMGAVKLAKLLIDKMKCEVTKAC

>starPep_00760

GWRTLLKKAEVKTVGKLALKHYL

>starPep_26917

KKLIKVFAKGWKKAKKLFKGIG

>starPep_26916

KKLIKVFAKGFKKAKKLWKGIG

>starPep_26918

KKLIKVWAKGFKKAKKLFKGIG

>starPep_02610

GLLRRLRDFLKKIGEKFKKIGY

>starPep_29236

LPRFSTMPFIYCNINEVCHY

>starPep_11025

KWKLFKKIGIGKFLHSATTF

>starPep_11010

KWKKFLKIGIGKFLHLAKKF

>starPep_11015

KWKLFAKIGIGKFLHLAKKF

>starPep_00517

KWKLFKKIGIGKFLHSAKKF

>starPep_11021

KWKLFKKIGIGAVLKVLKKG

>starPep_11019

KWKLFKKIGIGAFLHLAKKF

>starPep_11023

KWKLFKKIGIGKFLHLAKKF

>starPep_11020

KWKLFKKIGIGAFLHSAKKF

>starPep_10217

HLRRINKLLTRIGLYRHAFG

>starPep_11022

KWKLFKKIGIGKFKLAKKF

>starPep_00606

FFRLLFHGVHHVGKIKPRA

>starPep_03752

AKKVSKRLEKLFSKIQNDK

# Community 3

>starPep_01271

FALALKALKKALKKLKKALKKAL

>starPep_09360

FAKKLAKLAKKLAKLALAL

>starPep_09411

FALALKALKKLAKKLKKLAKKAL

>starPep_09352

FAKKLAKKLKKLAKKLAKLALALKALALKAL

>starPep_09371

FAKLFAKLAKKFAL

>starPep_09388

FAKLLAKLAKKFAL

>starPep_13353

VALALKALKKLAKKLKKLAKKAL

>starPep_09350

FAKKLAKKLKKLAKKLAKLAKKL

>starPep_07818

VAKKLAKLAKKLAKLALAL

>starPep_05251

FALKALKKLKKALKKAL

>starPep_09359

FAKKLAKLAKKLAKAL

>starPep_05247

FAKLLAKLAKKLL

>starPep_05246

FAKLLAKALKKLL

>starPep_02691

KKLALHALKKWLHALKKLAHLALKK

>starPep_13351

VALALKALKKALKKLKKALKKAL

>starPep_09401

FALAAKALKKLAKKLKKLAKKAL

>starPep_05250

FALALKALKKLLKKLKKLAKKAL

>starPep_09404

FALAKLAKKAKAKLKKALKAL

>starPep_09354

FAKKLAKKLKKLAKLALAK

>starPep_06266

KLAKKLAKLAKLAKAL

>starPep_09413

FALALKKALKALKKAL

>starPep_13347

VAKKLAKLAKKLLAL

>starPep_09361

FAKKLAKLAKKLLAL

>starPep_09362

FAKKLAKLALKLAKL

>starPep_09403

FALAKKALKKAKKAL

>starPep_09364

FAKKLKKLAKLAKKL

>starPep_09344

FAKKLAKKAKLAKKL

>starPep_09337

FAKALKALLKALKAL

>starPep_09374

FAKLLAKALKKFAL

>starPep_09345

FAKKLAKKLAKAAL

>starPep_13859

YAKLLAKLAKKAL

>starPep_13348

VAKLLAKALKKLL

>starPep_13350

VAKLLAKLAKKVL

>starPep_13344

VAKFLAKFLKKAL

>starPep_13349

VAKLLAKLAKKLL

>starPep_10502

KAKLAKKALAKLL

>starPep_03929

FLKLLKKLAAKLF

>starPep_09397

FAKLLKLAAKKLL

>starPep_09393

FAKLLAKLAKKVL

>starPep_09348

FAKKLAKKLAKLL

>starPep_09378

FAKLLAKFLKKAL

>starPep_09370

FAKLFAKAFKKAL

>starPep_09367

FAKLAKKALAKLL

>starPep_09381

FAKLLAKLAKAKA

>starPep_09375

FAKLLAKALKKFL

>starPep_09373

FAKLLAKALKKAL

>starPep_09386

FAKLLAKLAKKAL

>starPep_09396

FAKLLFKALKKAL

>starPep_09377

FAKLLAKALKLKL

>starPep_09394

FAKLLAKLAKLKL

>starPep_09387

FAKLLAKLAKKEL

>starPep_09382

FAKLLAKLAKAKG

>starPep_09385

FAKLLAKLAKKAA

>starPep_09390

FAKLLAKLAKKIL

>starPep_09383

FAKLLAKLAKAKL

>starPep_09392

FAKLLAKLAKKSL

>starPep_09372

FAKLLAKAFKKAL

>starPep_09389

FAKLLAKLAKKGL

>starPep_09338

FAKFLAKFLKKAL

>starPep_09336

FAKALAKLAKKLL

>starPep_09414

FALALKLAKKAL

>starPep_09420

FALLKALLKKAL

>starPep_09363

FAKKLKKLAKKL

>starPep_09365

FAKKLLAKALKL

>starPep_09342

FAKKALKALKKL

>starPep_11180

LKKLAKLALAF

>starPep_09415

FALALKLAKKL

>starPep_09418

FALKALKKAL

>starPep_09356

FAKKLAKKLL

>starPep_09406

FALALKAKKL

>starPep_09379

FAKLLAKKLL

>starPep_09343

FAKKLAKALL

# Community 4

>starPep_02587

GIPCAESCVWIPPCTITALMGCSCKNNVCYNN

>starPep_00048

DCYCRIPACIAGERRYGTCIYQGRLWAFCC

>starPep_23182

GLPTCGETCFKGKCYTPGCSCSYPICKKD

>starPep_00023

DHYNCVSSGGQCLYSACPIFTKIQGTCYRGKAKCCK

>starPep_01761

GGTIFDCGESCFLGTCYTKGCSCGEWKLCYGTN

>starPep_00249

ACGILHDNCVYVPAQNPCCRGLQCRYGKCLVQV

>starPep_09663

GAFLKCGESCVYLPCLTTVVGCSCQNSVCYRD

>starPep_01824

GVIPCGESCVFIPCISSVLGCSCKNKVCYRD

>starPep_02654

GVPCAESCVWIPCTVTALLGCSCKDKVCYLN

>starPep_01091

GTFPCGESCVFIPCLTSAIGCSCKSKVCYKN

>starPep_10080

GTLPCGESCVWIPCISSVVGCACKSKVCYKD

>starPep_01818

GTLPCGESCVWIPCISSVVGCSCKSKVCYKD

>starPep_01427

GSIPCGESCVFIPCISSVIGCACKSKVCYKN

>starPep_01425

GSIPCGESCVFIPCISAIIGCSCSSKVCYKN

>starPep_10029

GSIPCGESCVFIPCISAIIGCSCSNKVCYKN

>starPep_01426

GSIPCGESCVFIPCISAVIGCSCSNKVCYKN

>starPep_02247

GSIPCEGSCVFIPCISAIIGCSCSNKVCYKN

>starPep_01058

GLLPCAESCVYIPCLTTVIGCSCKSKVCYKN

>starPep_02589

GIPCGESCVFIPCTVTALLGCSCKDKVCYKN

>starPep_02595

GIPCGESCVYIPCTVTALLGCSCKDKVCYKN

>starPep_05617

GIPCAESCVWIPCTVTALLGCSCKDKVCYLD

>starPep_09675

GAVPCGETCVYLPCITPDIGCSCQNKVCYRD

>starPep_16391

CGESCVFIPCISSVIGCACKSKVCYKNGSIP

>starPep_16389

CGESCVFIPCISAIIGCSCSSKVCYKNGSIP

>starPep_16390

CGESCVFIPCISAVIGCSCSNKVCYKNGSIP

>starPep_16336

CEGSCVFIPCISAIIGCSCSNKVCYKNGSIP

>starPep_04109

GSVIKCGESCLLGKCYTPGCTCSRPICKKD

>starPep_10082

GTSCGETCVLLPCLSSVLGCTCQNKRCYKD

>starPep_05737

GLPCGESCVFIPCITTVVGCSCKNKVCYNN

>starPep_01370

GIPCGESCVWIPCLTSAIGCSCKSKVCYRN

>starPep_00307

GIPCGESCVWIPCISSAIGCSCKSKVCYRN

>starPep_02199

GIPCGESCVFIPCLTSAIDCSCKSKVCYRN

>starPep_09845

GIPCGESCVFIPCLTSAIGCSCKSKVCYRN

>starPep_01027

GIPCAESCVWIPCTVTALIGCGCSNKVCYN

>starPep_01030

GIPCGESCVFIPCITGAIGCSCKSKVCYRN

>starPep_01777

GIPCGESCVWIPCITSAIGCSCKSKVCYRN

>starPep_01029

GIPCGESCVFIPCITAAIGCSCKSKVCYRN

>starPep_01776

GIPCGESCVFIPCISSVIGCSCSSKVCYRN

>starPep_01327

GEFLKCGESCVQGECYTPGCSCDWPICKKN

>starPep_16393

CGESCVFIPCLTSAIDCSCKSKVCYRNGIP

>starPep_05106

CGESCVWIPCISSAIGCSCKSKVCYRNGIP

>starPep_16392

CGESCVFIPCISSVIGCSCSSKVCYRNGIP

>starPep_00021

ACYCRIPACIAGERRYGTCIYQGRLWAFCC

>starPep_09940

GLPTCGETCFKGKCYTPGCSCSYPICKKN

>starPep_09866

GKPICGETCFKGKCYTPGCTCSYPICKKN

>starPep_09878

GKYTCGETCFKGKCYTPGCTCSYPICKKD

>starPep_09865

GKPICGETCFKGKCYTPGCTCSYPICKKD

>starPep_01741

GEYCGESCYLIPCFTPGCYCVSRQCVNKN

>starPep_01230

AIPCGESCVWIPCISTVIGCSCSNKVCYR

>starPep_01470

IPCGESCVWIPCITAIAGCSCKNKVCYT

>starPep_01118

IPCGESCVWIPCISGMFGCSCKDKVCYS

>starPep_01762

GIACGESCVFLGCFIPGCSCKSKVCYFN

# Community 5

>starPep_28995

LLKKKFLKKQ

>starPep_28994

LLKKKFKLKQ

>starPep_29021

LLLKKFKKKQ

>starPep_29022

LLLKKFKKLQ

>starPep_29005

LLKLKFKKKQ

>starPep_29006

LLKLKFKKLQ

>starPep_28993

LLKKKFKKLQ

>starPep_28997

LLKKLFKKKQ

>starPep_28833

LKLKKFLKKQ

>starPep_28794

LKKLLFKKKQ

>starPep_28831

LKLKKFKKLQ

>starPep_28785

LKKKLFKLKQ

>starPep_28842

LKLLKFKKKQ

>starPep_28792

LKKLKFLKKQ

>starPep_28840

LKLKLFKKKQ

>starPep_28777

LKKKKFLKLQ

>starPep_28775

LKKKKFKLLQ

>starPep_28790

LKKLKFKKLQ

>starPep_28786

LKKKLFLKKQ

>starPep_28778

LKKKKFLLKQ

>starPep_27194

KLLLKFKKKQ

>starPep_27151

KLKKLFKKLQ

>starPep_27144

KLKKKFLLKQ

>starPep_27179

KLLKKFKLKQ

>starPep_27143

KLKKKFLKLQ

>starPep_27164

KLKLLFKKKQ

>starPep_27141

KLKKKFKLLQ

>starPep_27180

KLLKKFLKKQ

>starPep_27152

KLKKLFKLKQ

>starPep_27153

KLKKLFLKKQ

>starPep_27183

KLLKLFKKKQ

>starPep_27178

KLLKKFKKLQ

>starPep_26940

KKLLKFLKKQ

>starPep_26939

KKLLKFKLKQ

>starPep_26823

KKKKLFLLKQ

>starPep_26923

KKLKKFLKLQ

>starPep_26943

KKLLLFKKKQ

>starPep_26840

KKKLLFKKLQ

>starPep_26821

KKKKLFKLLQ

>starPep_26938

KKLLKFKKLQ

>starPep_26842

KKKLLFLKKQ

>starPep_26837

KKKLKFLKLQ

>starPep_26838

KKKLKFLLKQ

>starPep_26933

KKLKLFLKKQ

>starPep_26836

KKKLKFKLLQ

>starPep_26932

KKLKLFKLKQ

>starPep_26921

KKLKKFKLLQ

>starPep_26841

KKKLLFKLKQ

>starPep_26924

KKLKKFLLKQ

>starPep_26931

KKLKLFKKLQ

# Community 6

>starPep_23258

GLPVCGETCVGGTCNTPGCTCSWWPVCTRN

>starPep_01828

GVPVCGETCFGGTCNTPGCSCDPWPVCSRN

>starPep_01802

GLPVCGETCFGGTCNTPGCSCDPWPMCSRN

>starPep_01800

GLPVCGETCFGGTCNTPGCACDPWPVCTRD

>starPep_01408

GLPVCGETCFGGTCNTPGCSCETWPVCSRN

>starPep_01409

GLPVCGETCFGGTCNTPGCTCDPWPVCTRN

>starPep_21211

GETCFGGTCNTPGCTCDPWPVCTRNGLPVC

>starPep_08662

CGETCVGGTCNTPGCTCSWPVCTRNGLNPV

>starPep_40803

TCTLGTCYTAGCSCSWPVCTRNGVPICGE

>starPep_24427

GYPICGESCVGGICNIPGCSCSWPVCTTN

>starPep_01827

GVPICGETCVGGTCNTPGCSCSWPVCTRN

>starPep_00210

GVPICGETCTLGTCYTAGCSCSWPVCTRN

>starPep_00728

GLPVCGETCVGGTCNTPGCSCSWPVCTRN

>starPep_09942

GLPTCGETCTLGKCNTPKCTCNWPICYKN

>starPep_01410

GLPVCGETCVGGTCNTPGCACSWPVCTRN

>starPep_00726

GLPTCGETCTLGTCYVPDCSCSWPICMKN

>starPep_04076

GLPLCGETCVGGTCNTPGCSCGWPVCVRN

>starPep_01404

GLPICGETCVGGSCNTPGCSCSWPVCTRN

>starPep_09943

GLPVCGETCFTGSCYTPGCSCNWPVCNRN

>starPep_01073

GLPVCGETCTLGTCYTQGCTCSWPICKRN

>starPep_09944

GLPVCGETCVGGTCNTPGCSCSWPVCFRN

>starPep_01071

GLPICGETCVGGTCNTPGCSCSWPVCTRN

>starPep_23271

GLPVCGETCVGGTCYTPGCTCSWPVCTRN

>starPep_23204

GLPVCGETCTLGTCSTQGCTCSWPICKRN

>starPep_23260

GLPVCGETCVGGTCNTPGCTCSYPVCTRN

>starPep_09939

GLPICGETCVGGTCNTPGCFCTWPVCTRN

>starPep_01406

GLPVCGETCAGGTCNTPGCSCSWPICTRN

>starPep_23210

GLPVCGETCVGGTCDTPGCTCSWPVCTRN

>starPep_00727

GLPVCGETCFGGTCNTPGCSCTWPICTRD

>starPep_23268

GLPVCGETCVGGTCNYPGCTCSWPVCTRN

>starPep_01411

GLPVCGETCVGGTCNTPGCGCSWPVCTRN

>starPep_09941

GLPTCGETCTLGKCNTPKCTCNWPICYKD

>starPep_23213

GLPVCGETCVGGTCNSPGCTCSWPVCTRN

>starPep_01407

GLPVCGETCFGGRCNTPGCTCSYPICTRN

>starPep_21210

GETCAGGTCNTPGCSCSWPICTRNGLPVC

>starPep_01739

GDACGETCFTGICFTAGCSCNPWPTCTRN

>starPep_01737

GASCGETCFTGICFTAGCSCNPWPTCTRN

>starPep_16413

CGETCVGGTCNTPGCGCSWPVCTRNGLPV

>starPep_16412

CGETCVGGTCNTPGCACSWPVCTRNGLPV

>starPep_08658

CGETCTLGTCYTAGCSCSWPVCTRNGVPI

>starPep_02483

CGETCVGGTCNTPGCTCSWPVCTRNGLPV

# Community 7

>starPep_00011

FLPLLAGLAANFLPTIICKISYKC

>starPep_02134

FLPLIAGVAANFLPKIFCLISKKC

>starPep_02141

FLPVIASVAAKVLPKVFCFITKKC

>starPep_00628

FLPLAVSLAANFLPKLFCKITKKC

>starPep_02135

FLPLIAGVAASILPKIFCFITKKC

>starPep_00634

FLPVIAGVAANFLPKLFCAISKKC

>starPep_00968

FLPIIAGVAAKVLPKIFCAISKKC

>starPep_00429

FLPIIAGIAAKFLPKIFCTISKKC

>starPep_00619

FLPIIAGAAAKVVEKIFCAISKKC

>starPep_00055

FLPILASLAAKFGPKLFCLVTKKC

>starPep_00423

FLPAIVGAAAKFLPKIFCAISKKC

>starPep_03042

FLGALFKVASKVLPSVKCAITKKC

>starPep_02124

FLGAIAGVAAKFLPKVFCFITKKC

>starPep_19502

FFPGIIKVAGAILPTAICAITKRC

>starPep_19503

FFPGIIKVASAILPTAICAITKRC

>starPep_00282

FLSLALAALPKFLCLVFKKC

>starPep_20041

FLPKLLAGLPSFLCLVFKKC

>starPep_00637

FLSLALAALPKLFCLIFKKC

>starPep_03060

FLPLLLSALPSFLCLVFKKC

>starPep_20074

FLPLLLAGLPSFLCLVFKKC

# Community 8

>starPep_38527

RTCESQSHRFKGPCARDSNCATVCLTEGFSGGDCRGFRRRCFCTRPC

>starPep_01487

KSCCPNTTGRNIYNTCRFAGGSRERCAKLSGCKIISASTCPSDYPK

>starPep_02292

KSCCPNTTGRNIYNTCRLTGSSRETCAKLSGCKIISASTCPSNYPK

>starPep_01128

KSCCPNTTGRNIYNACRLTGAPRPTCAKLSGCKIISGSTCPSDYPK

>starPep_36368

RECKTESNTFPGICITKPPCRKACISEKFSGGDCSKILRRCLCTKPC

>starPep_04335

KSCCPSTTGRNIYNTCRLTGSSRETCAKLSGCKIISASTCPSNYPK

>starPep_03814

CETPSKHFNGLCIRSSNCASVCHGEHFTDGRCQGVRRRCMCLKPC
