# Supplementary material for: Automatic construction of molecular similarity networks for visual graph mining in chemical space of bioactive peptides: an unsupervised learning approach
Source: Sci Rep. 2020 Oct 22;10:18074. doi: 10.1038/s41598-020-75029-1 (PMC7583304; doi:10.1038/s41598-020-75029-1)
Supplement: Supplementary file 10 — Supplementary Information 9. [file 41598_2020_75029_MOESM10_ESM.zip › SI4_1-4_Anticancer_peptides/SI4-4_Anticancer_Central_NR70.fasta.docx]

**Supporting Information:**

Automatic construction of similarity networks for visual graph mining in chemical space of bioactive peptides: an unsupervised learning approach

Longendri Aguilera-Mendoza, Yovani Marrero-Ponce*, César R. García-Jacas, Edgar Chavez, Jesus A. Beltran, Hugo A. Guillen-Ramirez, Carlos A. Brizuela*.

Corresponding authors *: Y. Marrero-Ponce: ymarrero@usfq.edu.ec or ymarrero77@yahoo.es; Carlos A. Brizuela: cbrizuel@cicese.edu.mx

**Contents**: Central and non-redundant peptide sequences (FASTA format) that were identified in this study.

>starPep_08391

ALWKDLLKNVGKAAGKAVLNKVTDMVNQ

>starPep_00185

GFKDLLKGAAKALVKTVLF

>starPep_05855

GWRKWIKKATHVGKHIGKAALDAYI

>starPep_08395

ALWKTMLKKLGTVALHAGKAALGAVADTISQ

>starPep_01013

GIGKFLHAAKKFAKAFVAEIMNS

>starPep_01109

IKIPAFVKDTLKKVAKGVISAVAGALTQ

>starPep_21927

GFSSIFRGVAKFASKGLGKKLAKLGVKLVACKISKQC

>starPep_00640

FLSLIPHIVSGVASIAKHF

>starPep_01337

GFMDTAKNVAKNVAVTLIDKLRCKVTGGC

>starPep_04575

NFAEIFAAVNKLIKQGVVKG

>starPep_02606

GLFVGLAKVAAHNNPAIAEHFQA

>starPep_00720

GLMDTIKGVAKTVAASWLDKLKCKITGC

>starPep_00115

GFVDFLKKVAGTIANVVT

>starPep_09835

GIMDTVKNAAKNLAGQLLDKLKCSITAC

>starPep_18164

DTAVTGLASPLSTGKILDQKAYSCANRLIVLCIENSFMTDARK

>starPep_00008

GWGSFFKKAAHVGKHVGKAALTHYL

>starPep_00483

GLWSKIKEAAKAAGKAALNAVTGLVNQGDQPS

>starPep_00855

SIGAKILGGVKTFFKGALKELASTYLQ

>starPep_14994

ALWKKILKNAGKAALNKINQIVQ

>starPep_05620

GIRKWFKKAAHVGKKVGKVALNAYL

>starPep_06287

KLKNFAIGVAQSLLNKASCKLSGQC

>starPep_03179

GLLSVFKGVLKTAGKNVAKNVAGSLLDQLKCKISGGC

>starPep_19552

FFSMIPKIAGGIASLVKNL

>starPep_25343

IKYLLVKLQGASQKTITLMLRRNNLYVMGYS

>starPep_00361

KWKVFKKIEKMGRNIRNGIVKAGPAIAVLGEAKAL

>starPep_24256

GVWGIAKIAGKVLGNILPHVFSSNQS

>starPep_00316

GLFDIVKKIAGHIVSSI

>starPep_11149

LGQSAASAHHAYIVLAIENSFMTASKKK

>starPep_00323

GLFKVLGSVAKHLLPHVAPVIAEK

>starPep_00126

GLFGKLIKKFGRKAISYAVKKARGKH

>starPep_16349

CETWRTETTGATGQASSLLSGRLLEQKAASCHNSYIVLCIENSFMTSFSK

>starPep_02535

FLHHIVGLIHHGLSLFGDRAD

>starPep_00301

GIGGVLLSAGKAALKGLAKVLAEKYAN

>starPep_32958

MQFITDLIKKAVDVFKGLFGNK

>starPep_00494

GWGSIFKHGRHAAKHIGHAAVNHYL

>starPep_41197

TQQAFQKFLAAVTSALGKQYH

>starPep_09888

GLFDVIKKVASVIKGL

>starPep_00760

GWRTLLKKAEVKTVGKLALKHYL

>starPep_26099

IWLTALKFLGKNLGKLAKQQLAKL

>starPep_00546

RWKIFKKIEKVGQNIRDGIVKAGPAVAVVGQAATI

>starPep_01283

FLFSLIPHAIGGLISAFK

>starPep_34474

NPEKALEKLIAIQKAIKGMLNGWFTGVGFRRKR

>starPep_07415

RGDLLRHVVKILSKYL

>starPep_38769

RWKIFKKIERVGQNVRDGIIKAGKAIQVLGTAKALGK

>starPep_00657

GFLGILFHGVHHGRKKALHMNSERRS

>starPep_02849

RKGWFKAMKSIAKFIAKEKLKEHL

>starPep_23443

GMWSKIKETAMAAAKEAAKAAGKTISDMIKQ

>starPep_00498

HFLGTLVNLAKKIL

>starPep_00807

KSSAYSLQMGATAIKQVKKLFKKWGW

>starPep_09578

FLSLIPKIATGIAALAKHL

>starPep_01650

ALWKNMLKGIGKLAGQAALGAVKTLVGAE

>starPep_00419

FLGALIKGAIHGGRFIHGMIQNHH

>starPep_00203

GLLDIVKKVVGAFGSL

>starPep_06206

KILRGVAKKIMRTFLRRISKDILTGKK

>starPep_00570

AIGSILGALAKGLPTLISWIKNR

>starPep_02121

FIHHIIGGLFSVGKHIHSLIHGH

>starPep_00569

AGWGSIFKHIFKAGKFIHGAIQAHND

>starPep_05979

ILGKLLSTAAKLLSKL

>starPep_01097

GWKKWFNRAKKVGKTVGGLAVDHYL

>starPep_24842

HTHQDFQPVLHLVALNTPLSGGMRGIR

>starPep_03758

ALWDTLLKKVLKAAAKAALDAVLVGANA

>starPep_22216

GIFPIFAKLLGKVIKVASSLISKGRTK

>starPep_02206

GLFDIVKKVVGTIAGL

>starPep_25236

IKIPSFFRNILKKVGKKAVSLIAGALKQS

>starPep_00089

SWLSKTAKKLENSAKKRISEGIAIAIQGGPR

>starPep_22239

GIGAVLKVLTTGLPALKSWIKRKRQQ

>starPep_00484

GLWSKIKEVGKEAAKAAAKAAGKAALGAVSEAV

>starPep_07864

VLLVTLTRLHQRGVIYRKWRHFSGRKYR

>starPep_01265

DSMGAVKLAKLLIDKMKCEVTKAC

>starPep_02568

GFIFHIIKGLFHAGKMIHGLV

>starPep_00005

FFHHIFRGIVHVGKTIHRLVTG

>starPep_20670

GANLAKKFYTYINKFINYAW

>starPep_18007

DPFFKVPVNKLAAAVSNFGYDLYRVRSSTSPTTN

>starPep_00759

GWKKWLRKGAKHLGQAAIKGLAS

>starPep_01280

FFSLLPSLIGGLVSAIK

>starPep_29425

LRSRGELVAKFLAGEQSPEDYVAE

>starPep_21918

GFRKRFNKLVKKVKHTIKETANVSKDVAIVAGSGVAVGAAM

>starPep_10217

HLRRINKLLTRIGLYRHAFG

>starPep_26158

IYSFDGRDIMTDPSWPQKVIWHGSSPHGVRLVDNYCEAWRTA

>starPep_11020

KWKLFKKIGIGAFLHSAKKF

>starPep_06547

LGGIVSAVKKIVDFLG

>starPep_00311

GLFDIAKKVIGVIGSL

>starPep_03498

MNFNKLFVFVALVLAVCIGQSEAGWLKKIGKKIERVGQHTRDATIQTIGVAQQAANVAAT

LKG

>starPep_00011

FLPLLAGLAANFLPTIICKISYKC

>starPep_01288

FLGMIPGLIGGLISAFK

>starPep_00051

FFGWLIKGAIHAGKAIHGLIHRRRH

>starPep_00606

FFRLLFHGVHHVGKIKPRA

>starPep_25297

IKLSPKTKDNLKKVLKGAIKGAIAVAKMV

>starPep_00970

FLPIVGKLLSGLSGLL

>starPep_26917

KKLIKVFAKGWKKAKKLFKGIG

>starPep_00182

GFFALIPKIISSPLFKTLLSAVGSALSSSGGQE

>starPep_00073

GLFDIIKKIAESF

>starPep_00429

FLPIIAGIAAKFLPKIFCTISKKC

>starPep_22064

GGLKKLGKKLEGAGKRVFNAAEKALPVVAGAKALRK

>starPep_01457

IFGAIWKGISSLL

>starPep_09539

FLGAIAQALTSLLGKL

>starPep_02776

MPRWRLFRRIDRVGKQIKQGILRAGPAIALVGDARAVG

>starPep_27320

KNECLWTDMLSNFGYPGYQSKHYACIRQKG

>starPep_07120

NLVSALIEGRKYLKNVLKKLNRLKEKNKAKNSKENN

>starPep_01286

FLGALWNVAKSVF

>starPep_03927

FLGALFKALSKLL

>starPep_01308

FLSTIWNGIKSLL

>starPep_01723

FLPAALAGIGGILGKLF

>starPep_00791

ISRLAGLLRKGGEKIGEKLKKIGQKIKNFFQKLVPQPE

>starPep_02584

GILSKLGKALKKAAKHAAKA

>starPep_11918

MRKWFHNVLSSGQLLADKWPAWDYNRK

>starPep_02610

GLLRRLRDFLKKIGEKFKKIGY

>starPep_01646

AGYLLGKINLKALAALAKKIL

>starPep_00524

LLGMIPLAISAISALSKL

>starPep_00628

FLPLAVSLAANFLPKLFCKITKKC

>starPep_03315

KFFKRLLKSVRRAVKKFRKKPRLIGLSTLL

>starPep_00795

KFFRKLKKSVKKRAKEFFKKPRVIGVSIPF

>starPep_00205

GLLQTIKEKLESLESLAKGIVSGIQA

>starPep_19949

FLKGIVGKLGKLF

>starPep_20041

FLPKLLAGLPSFLCLVFKKC

>starPep_00637

FLSLALAALPKLFCLIFKKC

>starPep_01133

KWKLFKKIGIGAVLKVLTTG

>starPep_01847

ILPILSLIGGLLGK

>starPep_02672

ILGAILPLVSGLLSNKL

>starPep_03196

GLVGTLLGHIGKAILS

>starPep_41038

TLPFAYCNIHQVCHYAQRNDRSYWL

>starPep_19503

FFPGIIKVASAILPTAICAITKRC

>starPep_09335

FAKAIAKIAFGKGIGKVGKKLL

>starPep_25171

IIGPVLGLIGKALGGLL

>starPep_05375

FLKLLAGLLKNFA

>starPep_08069

YHWYGYTPQNVIGGGKLLLKLLKKLLKLLKKK

>starPep_29236

LPRFSTMPFIYCNINEVCHY

>starPep_09845

GIPCGESCVFIPCLTSAIGCSCKSKVCYRN

>starPep_09675

GAVPCGETCVYLPCITPDIGCSCQNKVCYRD

>starPep_09663

GAFLKCGESCVYLPCLTTVVGCSCQNSVCYRD

>starPep_01741

GEYCGESCYLIPCFTPGCYCVSRQCVNKN

>starPep_10080

GTLPCGESCVWIPCISSVVGCACKSKVCYKD

>starPep_09940

GLPTCGETCFKGKCYTPGCSCSYPICKKN

>starPep_00023

DHYNCVSSGGQCLYSACPIFTKIQGTCYRGKAKCCK

>starPep_04109

GSVIKCGESCLLGKCYTPGCTCSRPICKKD

>starPep_10082

GTSCGETCVLLPCLSSVLGCTCQNKRCYKD

>starPep_01327

GEFLKCGESCVQGECYTPGCSCDWPICKKN

>starPep_00021

ACYCRIPACIAGERRYGTCIYQGRLWAFCC

>starPep_01128

KSCCPNTTGRNIYNACRLTGAPRPTCAKLSGCKIISGSTCPSDYPK

>starPep_16390

CGESCVFIPCISAVIGCSCSNKVCYKNGSIP

>starPep_00249

ACGILHDNCVYVPAQNPCCRGLQCRYGKCLVQV

>starPep_01739

GDACGETCFTGICFTAGCSCNPWPTCTRN

>starPep_01027

GIPCAESCVWIPCTVTALIGCGCSNKVCYN

>starPep_09365

FAKKLLAKALKL

>starPep_03814

CETPSKHFNGLCIRSSNCASVCHGEHFTDGRCQGVRRRCMCLKPC

>starPep_09361

FAKKLAKLAKKLLAL

>starPep_09367

FAKLAKKALAKLL

>starPep_11180

LKKLAKLALAF

>starPep_09372

FAKLLAKAFKKAL

>starPep_09397

FAKLLKLAAKKLL

>starPep_09941

GLPTCGETCTLGKCNTPKCTCNWPICYKD

>starPep_01761

GGTIFDCGESCFLGTCYTKGCSCGEWKLCYGTN

>starPep_38527

RTCESQSHRFKGPCARDSNCATVCLTEGFSGGDCRGFRRRCFCTRPC

>starPep_40803

TCTLGTCYTAGCSCSWPVCTRNGVPICGE

>starPep_09420

FALLKALLKKAL

>starPep_36368

RECKTESNTFPGICITKPPCRKACISEKFSGGDCSKILRRCLCTKPC

>starPep_01071

GLPICGETCVGGTCNTPGCSCSWPVCTRN

>starPep_09415

FALALKLAKKL

>starPep_05251

FALKALKKLKKALKKAL

>starPep_09381

FAKLLAKLAKAKA

>starPep_09403

FALAKKALKKAKKAL

>starPep_13344

VAKFLAKFLKKAL

>starPep_03929

FLKLLKKLAAKLF

>starPep_09371

FAKLFAKLAKKFAL

>starPep_28778

LKKKKFLLKQ

>starPep_26842

KKKLLFLKKQ

>starPep_28842

LKLLKFKKKQ

>starPep_26821

KKKKLFKLLQ

>starPep_27179

KLLKKFKLKQ

>starPep_05058

ASVVNKLTGGVAGLLK

>starPep_03752

AKKVSKRLEKLFSKIQNDK
